# Risk-of-bias assessment of vaccine effectiveness studies: a scoping review of systematic reviews

**DOI:** 10.1017/S0950268826101794

**Published:** 2026-06-19

**Authors:** Zahra Davoodi, Cassandra Laurie, Kylie Tingley, Becky Skidmore, Nicole Shaver, Maria E. Sundaram, Deshayne B. Fell, Melissa Brouwers, Giorgia Sulis

**Affiliations:** 1 School of Epidemiology and Public Health, University of Ottawa Faculty of Medicine, Canada; 2 Independent epidemiologist, Canada; 3 Independent information specialist, Canada; 4 Marshfield Clinic Research Institute, USA; 5 Children’s Hospital of Eastern Ontario Research Institute, Canada; 6 Methodological and Implementation Research Program, Ottawa Hospital Research Institute, Canada

**Keywords:** evidence synthesis methods, methodological assessment, observational studies, quality appraisal, study quality, vaccination

## Abstract

Observational vaccine effectiveness (VE) studies provide essential real-world evidence but are prone to bias. Valid synthesis relies on rigorous risk-of-bias (RoB) assessment in systematic reviews of VE studies. Following JBI guidance, we mapped and described RoB assessment methodologies in systematic reviews of VE studies. We searched MEDLINE, Embase, and Web of Science from 1 January 2013 to 17 May 2023 and the grey literature from 1 January 2018 to 15 August 2023. Of 367 identified reviews, 38 lacked any RoB assessment, yielding 203 systematic reviews. Of these, 190 used existing tools (NOS (85/190, 44.7%), ROBINS-I (46/190, 24.2%), and JBI (11/190, 5.8%)) and 13 used an author-developed tool (13/203, 6.4%). Tools were adapted in 16.7% (34/203) of reviews and 7.2% (14/203) used multiple tools. Reviews included 20 (±25.7) observational studies, commonly cohorts (175/203, 86.2%), with COVID-19 (66/203, 32.5%) and seasonal influenza (62/203, 30.5%) frequently studied. VE was reported descriptively in 25.1% (51/203) of reviews, while 74.9% (152/203) provided meta-analyzed estimates primarily based on laboratory-confirmed infection (137/203, 67.5%) and symptomatic disease (130/203, 64.0%). Our findings indicate heterogeneous RoB assessment, reflected by use of different/multiple tools, frequent adaptations, author-developed methods, and absence of RoB assessment, highlighting the need for clearer guidance or tailored tools.

## Introduction

Vaccines are a cornerstone of global public health, playing a crucial role in reducing the burden of infectious diseases worldwide [1]. While randomized controlled trials (RCTs) generate evidence of vaccine efficacy under ideal conditions, post-licensure observational studies provide essential real-world evidence on vaccine effectiveness (VE) and public health impact across diverse populations and settings [2]. Systematic reviews and meta-analyses (SR/MAs) synthesize findings from these studies to inform clinical practice, public health policies, and vaccination strategies. However, the reliability of SR/MA findings is contingent upon the quality of the included studies and their risk-of-bias (RoB) [3].

Generic RoB assessment tools, designed to evaluate a broad spectrum of studies, may be insufficient in the context of VE research. Specifically, observational VE studies are susceptible to some important and often unique challenges compared to observational studies in other clinical areas, such as healthy vaccinee bias, healthcare-seeking behavior bias, temporal bias, and biases related to prior infection or immunization schedules [4]. Furthermore, across observational study designs, the types of biases vary. For instance, cohort studies may be affected by differential loss to follow-up and difficulties in accounting for changes in vaccination status, while case-control studies face risks of recall bias, misclassification of vaccination history, and challenges in selecting appropriate control groups [3]. The increasingly popular case-control study using a test-negative design (TND) mitigates certain biases (e.g., healthcare-seeking behavior bias) by comparing symptomatic individuals who seek care and test positive for the target pathogen (cases) with those who test negative (controls) [5]; however, the TND design, when poorly designed or implemented, introduces its own challenges, including heterogeneity in testing procedures, case definitions, and diagnostic tools [4, 6].

As in other areas of research, SR/MAs of observational VE studies also face challenges due to more pronounced clinical heterogeneity compared to SR/MAs of RCTs, stemming from variations in participant characteristics, interventions, and outcomes, reflecting real-world practices and less stringent inclusion criteria [3]. Heterogeneity in exposure and outcome definitions and measurements across VE studies underscore the need for standardized reporting and rigorous RoB assessment to ensure the validity of pooled evidence from VE research.

To gain a more comprehensive understanding of current practices and explore the landscape of RoB assessment in VE research, we conducted a scoping review with the objective of identifying tools currently used to assess RoB in VE studies in published systematic reviews with particular attention to the use of current RoB standards, modifications to current standards, and the application of new original strategies employed by the authors.

## Methods

We prospectively registered our scoping review protocol on the Open Science Framework (https://osf.io/euz9b/) [7]. We followed the Joanna Briggs Institute (JBI) methodological framework for scoping reviews, initially developed by Arksey and O’Malley [8] and subsequently refined by Levac et al. [9]. Additionally, this review is reported in accordance with the Preferred Reporting Items for Systematic Reviews and Meta-Analyses extension for Scoping Reviews (PRISMA-ScR) checklist [10] and PRISMA 2020 statement [11]. A completed PRISMA-ScR checklist is provided in Supplementary Appendix A. While our protocol initially specified an English-only inclusion criterion, we ultimately did not impose language restrictions, allowing the inclusion of studies published in any language.

### Search strategy

A professional information specialist (BS), in consultation with the research team, developed the search strategy. The MEDLINE strategy was peer-reviewed by another senior information specialist using the Peer-Review of Electronic Search Strategies (PRESS) checklist [12] (Supplementary Appendix B). Using the multifile option and deduplication tool on the Ovid platform, we searched Ovid MEDLINE® ALL and Embase Classic+Embase. We also searched the core databases of Web of Science. The search combined controlled vocabulary (e.g. ‘Vaccine Efficacy’, ‘Vaccines’, ‘Systematic Review’) with relevant free text (e.g. ‘vaccine effectiveness’, ‘immunization’, ‘evidence-based review’). We executed all searches on 17 May 2023. There were no language restrictions. To ensure feasibility and budgetary parameters, we limited results to the publication years 2013–2023. We downloaded the records to EndNote 9.3.3 (Clarivate Analytics) and deduplicated them prior to uploading them to Covidence (Veritas Health Innovation Ltd). Details of the full search strategy are provided in Supplementary Appendix C. To identify the relevant grey literature and unpublished studies, we utilized the Grey Matters checklist developed by the Canadian Drug Agency (CAD-AMC) [13] to include materials published between 1 January 2018 and 15 August 2023 (Supplementary Appendix D). Our searches were not updated after these dates given the methodological focus of this scoping review and the achievement of saturation.

### Selection criteria

Eligibility criteria were defined using the Population-Concept-Context (PCC) framework [7]. We included systematic reviews of observational VE studies, with or without meta-analysis, that performed *any* form of RoB or quality assessment, whether using an established tool or the review authors’ own methodology. To maximize the capture of relevant data, we imposed no restrictions on population characteristics, vaccine types, or vaccine-preventable diseases. For the purpose of this review, we defined VE as any measure of the vaccine’s protective capacity against various outcomes under real-world conditions, evaluated through post-licensure (phase IV) studies [14]. Systematic reviews that solely included RCTs evaluating vaccine efficacy or studies focused solely on immunogenicity outcomes (e.g. antibody titres, markers of cell-mediated responses) were excluded.

### Review process

We imported the search results into Covidence (Veritas Health Innovation Ltd) for final deduplication and screening. The study selection process consisted of two stages. First, two independent reviewers screened all unique records by title and abstract, retaining any record selected by at least one reviewer. In the second stage, two reviewers independently conducted full-text screenings of all records selected in the previous stage, resolving any disagreements through team discussions until consensus was reached. To ensure consistency at each stage, all reviewers initially screened the same 10 publications. The results from this pilot phase informed refinements to the screening process. For non-English publications, we utilized our team’s language capabilities and employed ChatGPT (OpenAI) for translation when needed.

### Data extraction and synthesis

We developed and piloted a standardized data extraction form in Covidence to systematically collect relevant information from the included systematic reviews (Supplementary Appendix E). A single reviewer extracted data from the included full-text articles using the form, which was verified by another reviewer. The following elements were extracted: study design(s) included in the systematic review, number of observational studies included, vaccine(s) assessed, definition(s) of VE, outcomes measured, and details about whether and how the review authors conducted RoB assessment(s) of included studies. For the purposes of our review, we included GRADE [15] and CHERG [16] as RoB tools when no other designated RoB tool was used. Although technically GRADE is an evidence assessment framework, one of the key domains is study limitations/RoB, which is represented by five key criteria relevant to observational studies: ‘failure to develop and apply appropriate eligibility criteria, flawed measurement of both exposure and outcome, failure to adequately control confounding, and incomplete or inadequately short follow-up’ [17]. Similarly, CHERG was derived from the GRADE framework and adapts its principles for assessing the quality of evidence on intervention effects relevant to child health [16]. Separately, we also documented instances where GRADE or CHERG were used alongside a designated RoB tool. Discrepancies during data extraction were resolved through discussion to maintain consistency and accuracy. Descriptive statistics were used to summarize the data.

## Results

### Search results

**Figure 1.**
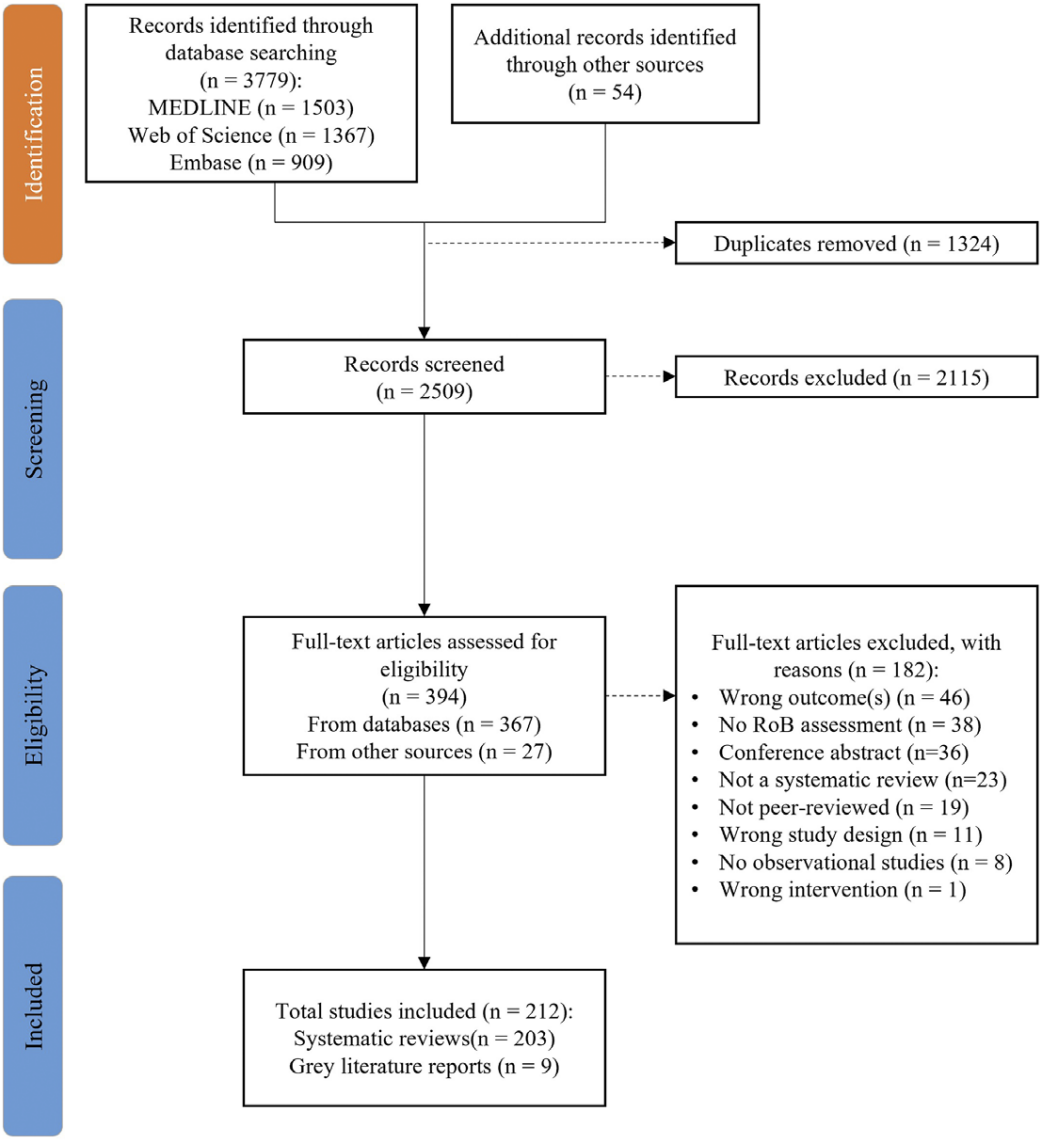
The PRISMA flow diagram of study selection [54]. Figure 1. long description.

Our searches identified 2,509 unique records from three databases and 54 reports from the grey literature sources, all of which underwent title and abstract screening (Figure 1). From the databases, 367 records were eligible for full-text assessment, including 8 (2.2%) published in languages other than English (Chinese, French, Portuguese, Korean, and Farsi). Of these, 203 systematic reviews (202 in English and one in Portuguese) met our inclusion criteria and were included in the data synthesis (Supplementary Table S1, Supplementary Appendix F). A list of excluded studies with reasons for exclusion is provided in Supplementary Appendix G. Notably, 38 systematic reviews were excluded because no RoB or quality assessment was reported, thus making them ineligible (Figure 1).

From the 54 records identified through the grey literature sources, 27 remained after duplicate removal and were screened, yielding 9 eligible reports. Although these were not systematic reviews and thus excluded from the descriptive analysis, we reviewed their RoB assessment approaches. We have summarized these reports in Supplementary Appendix F and Supplementary Table S5, highlighting their valuable methodologies for quality assessment, despite not being discussed further in the main text.

### Characteristics of the systematic reviews

Of the 203 systematic reviews included in our analysis, 112 (55.2%) included both RCTs and observational studies (Table 1). The systematic reviews included various observational study designs: cohort studies (175/203, 86.2%), case-control studies (131/203, 64.5%), and TND studies (45/203, 22.2%). On average, systematic reviews included 20 observational studies (range one to 290); 37.9% of reviews incorporated fewer than 10 primary studies and 19.2% more than 30.

**Table 1.** Characteristics of systematic review articles included in the scoping review (*N* = 203) Table 1. long description.

Characteristic	*N*	%
**RCTs included in the systematic review**		
Yes	112	55.2%
No	91	44.8%
**Number of observational studies included in the systematic review**		
Mean (SD)	19.8 (±25.7)	
Minimum, maximum	1290	
<10	77	37.9%
10–20	61	30.0%
21–30	26	12.8%
>30	39	19.2%
**Observational study designs included in reviews^a^**		
Cohort	175	86.2%
Case – control	131	64.5%
Test-negative	45	22.2%
Cross-sectional	31	15.3%
Screening method	13	6.4%
Other	31	15.3%
Not reported	6	3.0%
**Vaccine-preventable disease under investigation^a^**		
COVID–19	66	32.5%
Seasonal influenza	62	30.5%
Pneumococcal disease	19	9.4%
HPV	15	7.4%
Varicella, Herpes zoster (shingles)	11	5.4%
Measles, Mumps, Rubella (MMR)	10	4.9%
Pandemic influenza	10	4.9%
Pertussis	8	3.9%
Rotavirus disease	7	3.4%
Other	18	8.9%
**Definitions of VE**		
**Descriptive** (VE as reported in primary studies)	51	25.1%
**Meta-analyzed** (VE based on combined adjusted effect estimates) **^a^**	152	74.9%
Based on odds ratio: (1−OR) × 100 **^a^**	89	58.6%
Based on risk ratio: (1−RR) × 100 **^a^**	73	48.0%
Based on hazard ratio: (1−HR) × 100 **^a^**	19	12.5%
Based on incidence rate ratio: (1−IRR) × 100 **^a^**	15	9.9%
Based on diagnostic odds ratio: (1−DOR) × 100 **^a^**	4	2.6%
Based on alternative VE/effect estimate calculations(s)^a^^,^^b^	13	8.6%
Type of effect estimate(s) not specified	10	6.6%
**Outcomes assessed^a^**		
Laboratory-confirmed infection	137	67.5%
Symptomatic disease	130	64.0%
Hospitalization	88	43.3%
Death	70	34.5%
Contact with HCP **^c^**	15	7.4%
Disease severity	4	2.0%
Other	9	4.4%
**Risk-of-bias assessment performed using a RoB tool or quality assessment framework?**		
Yes	190	93.6%
No	13	6.4%

a Categories are not mutually exclusive, e.g., reviews could report more than one effect measure or outcome.

b VE = [1 − {(PCV × (1 − PPV)) / ((1 − PCV) × PPV)}], (PCV: proportion of cases vaccinated, PPV: proportion of population vaccinated); VE = [1 − {(c1 / N1) / ((c0 + 1) / (N0 + 1))}], where VE = [(1 − RR) × 100], RR = (c1 / N1) (risk in the vaccinated group) and the risk in the unvaccinated group is adjusted with a continuity correction (c0 + 1) / (N0 + 1); VE as a dichotomous outcome (e.g., incidence: yes or no), RR = {OR / [(1 − P₀) + (P₀ × OR)]}, (P₀: event incidence in control group, OR: odds ratio); VE(t) = A × e^(−w × t)^, (A: initial effectiveness, w: waning rate, t: time); VE = [1 − (ARV/ARU) × 100], (ARV, ARU: attack rates in vaccinated and unvaccinated); VE = [(pooled HR − 1) / HR] or VE = [(pooled OR − 1) / OR]; Broome method; Pooled relative VE (rVE); Risk reduction.

c Examples include emergency department, urgent care or clinic/office visits.

HCP, healthcare professional; HPV, human papillomavirus; SD, standard deviation; RCT, randomized controlled trial; RoB, risk of bias; VE, vaccine effectiveness.

COVID-19 and seasonal influenza vaccines were the most frequently studied across included systematic reviews (66/203 (32.5%) and 62/203 (30.5%), respectively). Others commonly assessed included vaccines against pneumococcal disease (19/203, 9.4%) and human papillomavirus (HPV; 15/203, 7.4%). Outcomes evaluated included laboratory-confirmed infections (137/203, 67.5%), symptomatic disease (130/203, 64.0%), and hospitalization (88/203, 43.3%). The details are provided in Supplementary Table S1 (Supplementary Appendix F).

The included systematic reviews revealed diverse approaches to defining and calculating VE. Notably, 152/203 (74.9%) reviews reported meta-analysed VE estimates based on various adjusted effect measures, while 51/203 (25.1%) reviews reported VE descriptively as presented in the primary studies, without pooling. Among these 152 reviews, the most used effect measure was the odds ratio (OR), applied in 58.6% (89/152) of the reviews, using the formula VE = (1 − OR) × 100. The risk ratio (RR) was the second-most used measure, employed in 48.0% (73/152) of reviews, utilizing the formula VE = (1 − RR) × 100. Less commonly, hazard ratios were used in 19/152 reviews (12.5%), incidence rate ratios in 15/152 reviews (9.9%), and diagnostic odds ratios in 4/152 reviews (2.6%). Further details are provided in Table 1.

### RoB assessment methods

Most of the systematic reviews (190/203, 93.6%) reported using one (176/203, 86.7%) or multiple (14/203, 7.2%) existing RoB and/or quality-of-evidence assessment tools or checklists. As shown in Table 2, the Newcastle-Ottawa Scale (NOS) [18] was the most employed (85/190, 44.7%), followed by Risk of Bias in Non-randomized Studies of Interventions (ROBINS-I) (46/190, 24.2%) [19], and JBI tools (11/190, 5.8%) [20]. Ad-hoc author-developed tools were used in 13/203 (6.4%) reviews. Two quality assessment frameworks, GRADE and CHERG, were used to assess RoB or quality in 6 (3.2%) and 3 (1.6%) reviews, respectively. GRADE and CHERG were used alongside designated RoB tools in 37 and 1 review, respectively. Along with occasional instances of these certainty assessments (e.g. GRADE and CHERG) being used to assess RoB/quality, we identified other checklists and tools being used to assess RoB, such as reporting guidelines (e.g. STROBE) [21], tools designed for assessment of randomized trials (that can be used to assess prospective cohort studies) being applied to other study designs, such as case-control [22], and tools designed for animal studies (e.g. SYRCLE’s RoB tool) [23].

**Table 2.** Risk-of-bias assessment tools/checklists utilized in included systematic reviews (*n* = 190) Table 2. long description.

RoB tool/checklist^a^	N	%
Newcastle-Ottawa Scale (NOS)	85	44.7%
Risk of Bias in Non-randomized Studies of Interventions (ROBINS-I)	46	24.2%
Joanna Briggs Institute (JBI)	11	5.8%
Downs and Black Checklist	7	3.7%
Grading of Recommendations, Assessment, Development, and Evaluations (GRADE)^b^	6	3.2%
National Heart, Lung and Blood Institute (NHLBI) Study Quality Assessment Tool	6	3.2%
National Institutes of Health (NIH) quality assessment tool	6	3.2%
Cochrane RoB tool	5	2.6%
Checklist recommended by Agency for Healthcare Research and Quality (AHRQ) for cross-sectional/prevalence study quality	4	2.1%
Child Health Epidemiology Reference Group (CHERG)^c^	3	1.6%
Scottish Intercollegiate Guidelines Network (SIGN)	3	1.6%
Critical Appraisal Skills Programme (CASP)	3	1.6%
Other	20	10.5%

a Categories are not mutually exclusive as more than one tool was utilized in some systematic reviews.

b GRADE was reported to be used to assess quality and/or risk-of-bias. While technically GRADE is a quality assessment framework, one of the key domains is risk-of-bias. 37 of the systematic reviews used GRADE alongside a designated RoB tool.

c CHERG was reported to be used to assess quality and/or risk-of-bias. While technically CHERG is a quality assessment framework, one of the key domains is study quality. One of the of systematic reviews used CHERG alongside a designated RoB tool [55].

Among the 190 systematic reviews that utilized a published RoB assessment tool, 34 (17.9%) reported adapting the tool(s) to better align with the reviews’ goals. These adaptations were made to align with characteristics of included primary studies, such as specific study designs, vaccine types, or other relevant features. Detailed descriptions of these modifications and their implications are presented in Supplementary Tables S2 and S3 (Supplementary Appendix F). Common adjustments included modifications to published scoring systems, refinements to specific assessment criteria, or inclusion/exclusion checklist items. For instance, the authors of some reviews adapted the NOS by converting its star ratings into categorical risk levels (e.g. three or four categories from low to high RoB based on whether a certain number of items were missing or inadequate) [24–27], while others applied scoring cut-offs (e.g. categorizing a study into low/moderate/high quality based on the number of points/stars assigned to each item) to interpret the results [28–31]. Two systematic reviews customized the NOS by integrating items from other checklists [30, 31]. For example, Gupta et al. (2022) [30] modified the NOS by incorporating the three domains of selection, comparability, and outcome/exposure from the Agency for Healthcare Research and Quality (AHRQ) [32] framework and converting NOS scores to align with the AHRQ quality assessment thresholds. Other reviews made what could be perceived as more substantial modifications, such as removing non-relevant criteria or adapting the assessment criteria of the selected RoB tool to better align with the specific vaccines, populations, or outcomes under evaluation [33–37]. For instance, Hughes et al. [33] added context-specific assessment criteria to their RoB assessment, for example, studies were classified as being at a high risk of bias if they reported the following issues: cold chain issues, age groups spanning 10 years, and unclear or missing age at initial dose. Similarly, Markowitz et al. [37] adapted questions to the specific context, in this case, making them specific to reduced dose VE studies, e.g., assessing whether there was sufficient interval between the first and second vaccine dose and whether there was a sufficient buffer time between vaccination and case counting to avoid incorrectly counting prevalent infections.

Thirteen systematic reviews (6.4%) did not assess RoB through a commonly used tool (Supplementary Table S4 and Supplementary Appendix F). One review by Kandeil et al. (2020) [38] reported considering the GRADE framework to assess quality but ultimately did not use it, citing the diverse study outcomes and extensive data recalculations required. Instead, the quality of studies was assessed using a rubric designed by the review authors. Similarly, the remaining 12 reviews employed custom methods to assess biases and study validity. These custom methods often involved creating a checklist, scale, or resource that captured RoB and/or quality concepts the authors found relevant. In cases where no applicable tool was available for a given study design, the authors provided a narrative description of their quality assessment, commenting on quality characteristics often discussed in the literature [39–49]. For example, Darvishian et al (2014) [41] created a checklist and elicitation scale to evaluate internal and external biases focusing on their potential impact on VE estimates. Similarly, Andani et al. (2022) [46] developed an evaluation strategy targeting three key quality features related to case detection, data collection methods, and quantitative methods. This tool classified studies as weak, moderate, strong, or having a fatal error in each domain. Studies receiving a fatal error rating in any domain were excluded from further analysis. Similarly, in the absence of an RoB tool tailored to TND studies, review authors often rely on methodological features that are applicable across designs. For example, Okoli et al. (2021) [47] examined studies on three quality features that could introduce bias: participant enrollment methods, methods for verifying vaccination status, and statistical adjustments for age and comorbidities.

## Discussion

This scoping review identified 203 systematic reviews of observational VE studies, most of which assessed RoB using a validated risk-of-bias assessment tool. The systematic application of standardized tools and reporting guidelines is essential to promote research reproducibility and enhance the credibility of scientific findings used to inform clinical and policy decisions.

Importantly, our objective was not to evaluate the performance of individual RoB tools in detecting specific biases or in discriminating between low- and high-quality VE studies, as such an assessment would require reappraisal of primary studies. Instead, we aimed to document how systematic review authors apply RoB tools in practice and how they respond to perceived limitations. Because the included reviews did not usually report explicit rationales for modifying RoB tools, our findings should be interpreted as describing patterns of use and adaptation, rather than authors’ motivations.

Notwithstanding the widespread usage of RoB tools, and their frequent modification, such as the addition of criteria or alternative scoring approaches, our scoping review suggests significant heterogeneity of practices concerning the quality and risk-of-bias assessment of VE studies, reflecting limitations in how existing tools are operationalized within VE systematic reviews. It is noteworthy that in our initial screening 38 systematic reviews were excluded from our review due to the absence of *any* risk-of-bias or quality assessment.

Our findings also show that systematic reviewers often address the lack of tailored assessment tools for VE studies by modifying or adapting an existing tool, or in 13 cases, even developing their own methods. For example, Okoli et al. (2021) [47] created their own approach to overcome the lack of a validated assessment tool for TND studies, despite TND being a common design in VE research. TND studies are increasingly employed over traditional case-control studies because they help mitigate confounding from healthcare-seeking behavior by selecting cases and controls among those who seek care at the same facilities and present with similar symptoms and offer greater efficiency than cohort studies [50, 51].

The authors employed a diverse array of RoB or quality assessment tools, with NOS [18], ROBINS-I [19], and JBI [20] being the most frequently used. Notably, adaptations of these tools, particularly of NOS [18] and ROBINS-I [19], along with the Downs and Black checklist [52], were implemented in 34 (16.7%) included reviews and 14 (7.2%) resorted to using multiple RoB tools to evaluate the methodological quality of included studies, reflecting the limitations of individual tools in capturing the variability inherent in observational studies specifically aimed at VE estimation. Each design presents distinct challenges that need to be properly accounted for in risk-of-bias and quality assessment. While some of these methodological challenges may also arise in other areas of observational research, their recurrence within VE systematic reviews underscores persistent difficulty in applying existing RoB tools to assess biases central to VE estimation. Cross-sectional studies cannot establish temporal relationships and may overestimate VE when conducted during periods of peak effectiveness. TND studies, while reducing confounding from healthcare-seeking behavior, remain sensitive to differences in testing practices [51]. Screening method studies rely heavily on accurate estimates of population vaccination coverage, but they are prone to overestimating VE due to respondent bias and limited ability to control for confounding [50]. Cohort studies face issues such as differential loss to follow-up, immortal time bias [42] whereas case-control studies are vulnerable to recall bias and require careful selection of controls to ensure valid estimation of VE [53]. These biases are highlighted in the context of VE research in part because of the frequent reliance on observational designs and their importance for interpreting VE estimates. The complexity of VE estimation and the resulting difficulty of critically appraising VE studies in a standardized manner highlights the urgent need for a comprehensive, reliable, and user-friendly RoB tool tailored specifically to VE research, capable of addressing the unique challenges and biases associated with diverse observational study designs.

While certainty assessment frameworks such as GRADE [15] and CHERG [16] contain domains or criteria that include items related to RoB and/or quality, they are not technically RoB tools themselves. In addition, use of checklists, such as reporting guidelines, and application of tools intended for other designs or populations suggest a need for clarity. The occasional use of frameworks, checklists or other tools being used to assess RoB, the complete absence of RoB assessment, the frequent adaptations to tools, the use of multiple tools, and the use of ad hoc approaches together indicate a need for clearer guidance or a tailored framework for assessing RoB in VE systematic reviews.

Of the 203 systematic reviews, 51 (25.1%) reported VE descriptively, while 10 (4.9%) pooled data without specifying the effect estimates used to calculate VE, likely aiming to maximize the inclusion of highly diverse primary studies. From a methodological standpoint, failing to specify the type of effect estimate used for VE calculation in systematic reviews can complicate interpretation and synthesis. Different effect measures (e.g. odds ratios, risk ratios, hazard ratios) are not interchangeable, and pooling them without definition may lead to misleading or non-comparable results. This lack of clarity can hinder readers’ ability to assess the validity and applicability of pooled estimates and underscores the importance of transparent reporting of VE definitions and calculation methods in evidence syntheses.

In line with the descriptive nature of our review, we do not make recommendations about use of a single optimal RoB tool for VE systematic reviews. Instead, the observed patterns of tool adaptation, such as converting star ratings into categorical judgments or adding criteria to better reflect VE-specific concerns, ad hoc approaches, and use of multiple tools suggest that existing instruments may not fully meet the needs of VE evidence synthesis, highlighting a potential gap and the need for either the development of a VE-specific RoB tool or clearer, design-specific guidance on how existing tools should be applied and adapted across different observational VE study designs.

Our review has several strengths, including adherence to rigorous scoping review methods, independent duplicate screening and data extraction processes, and a comprehensive literature search that achieved saturation. However, heterogenous and suboptimal reporting practices across studies pose challenges in fully ascertaining risk of bias assessment methodologies, making it difficult to disentangle limitations of the RoB tools themselves from limitations in how they were applied, and therefore our findings reflect observed practices rather than the intrinsic adequacy of the tools. The quality of our review is inherently tied to the reporting quality of the included systematic reviews, underscoring once again the need for standardized and transparent reporting practices.

## Summary

In summary, the identification and evaluation of important sources of bias in observational VE studies is highly dependent on the context and specific characteristics of each study, including its design. Future research should focus on systematically evaluating the main types of bias to be considered, mitigated, and accounted for in VE estimation through various types of VE studies. This would lay the foundation for developing and validating a new RoB tool, or modifying existing ones, to address the features commonly encountered in VE research (e.g. diverse observational designs, time-varying exposure definitions, and the frequent need to adapt RoB tools to vaccination-specific contexts). Standardized reporting practices are equally critical to ensure that findings are robust, comparable, and capable of informing evidence-based clinical and policy decisions.

## Supporting information

10.1017/S0950268826101794.sm001 Davoodi et al. supplementary material Davoodi et al. supplementary material

## Data Availability

The authors confirm that the data supporting the findings of this study are available within the article and its supplementary materials.

## Abbreviations

VE: Vaccine effectiveness
RoB: Risk-of-bias

## Supplementary material

The supplementary material for this article can be found at http://doi.org/10.1017/S0950268826101794.

## Long descriptions

### Figure 1 Long description

The flowchart is organized into four vertical phases labeled on the left.

1. Identification phase. Two top boxes show records identified through database searching n = 3779 and other sources n = 54. Databases include MEDLINE n = 1503, Web of Science n = 1367, and Embase n = 909. A horizontal dashed arrow points to a box for Duplicates removed n = 1324.

2. Screening phase. A central box for Records screened n = 2509 leads to a horizontal dashed arrow pointing to Records excluded n = 2115.

3. Eligibility phase. A central box for Full-text articles assessed for eligibility n = 394 includes 367 from databases and 27 from other sources. A horizontal dashed arrow points to a box for Full-text articles excluded, with reasons n = 182. Reasons include: Wrong outcome(s) n = 46, No RoB assessment n = 38, Conference abstract n = 36, Not a systematic review n = 23, Not peer-reviewed n = 19, Wrong study design n = 11, No observational studies n = 8, and Wrong intervention n = 1.

4. Included phase. The final central box shows Total studies included n = 212, consisting of Systematic reviews n = 203 and Grey literature reports n = 9.

Navigate back to Figure 1.

### Table 1 Long description

The table is organized into seven main categories with corresponding counts (N) and percentages (%).

* RCTs included in the systematic review: Yes (112, 55.2%), No (91, 44.8%).

* Number of observational studies included: Mean (S D) is 19.8 (plus or minus 25.7); Minimum to maximum range includes a range from 1 to 1290. Distribution: less than 10 (77, 37.9%), 10 to 20 (61, 30.0%), 21 to 30 (26, 12.8%), and greater than 30 (39, 19.2%).

* Observational study designs: Cohort (175, 86.2%), Case-control (131, 64.5%), Test-negative (45, 22.2%), Cross-sectional (31, 15.3%), Screening method (13, 6.4%), Other (31, 15.3%), and Not reported (6, 3.0%).

* Vaccine-preventable disease under investigation: COVID19 (66, 32.5%), Seasonal influenza (62, 30.5%), Pneumococcal disease (19, 9.4%), HPV (15, 7.4%), Varicella or Herpes zoster (11, 5.4%), MMR (10, 4.9%), Pandemic influenza (10, 4.9%), Pertussis (8, 3.9%), Rotavirus (7, 3.4%), and Other (18, 8.9%).

* Definitions of VE: Descriptive (51, 25.1%) and Meta-analyzed (152, 74.9%). Meta-analysis was based on Odds Ratio (89, 58.6%), Risk Ratio (73, 48.0%), Hazard Ratio (19, 12.5%), Incidence Rate Ratio (15, 9.9%), Diagnostic Odds Ratio (4, 2.6%), Alternative calculations (13, 8.6%), and Not specified (10, 6.6%).

* Outcomes assessed: Laboratory-confirmed infection (137, 67.5%), Symptomatic disease (130, 64.0%), Hospitalization (88, 43.3%), Death (70, 34.5%), Contact with HCP (15, 7.4%), Disease severity (4, 2.0%), and Other (9, 4.4%).

* Risk-of-bias assessment performed: Yes (190, 93.6%), No (13, 6.4%).

Navigate back to Table 1.

### Table 2 Long description

The table consists of three columns: RoB tool/checklist, N, and percentage. The data is as follows:

* Newcastle-Ottawa Scale (N OS): 85, 44.7 percent.

* Risk of Bias in Non-randomized Studies of Interventions (R OBINS-I): 46, 24.2 percent.

* Joanna Briggs Institute (J BI): 11, 5.8 percent.

* Downs and Black Checklist: 7, 3.7 percent.

* Grading of Recommendations, Assessment, Development, and Evaluations (GRADE): 6, 3.2 percent.

* National Heart, Lung and Blood Institute (N HLBI) Study Quality Assessment Tool: 6, 3.2 percent.

* National Institutes of Health (N IH) quality assessment tool: 6, 3.2 percent.

* Cochrane RoB tool: 5, 2.6 percent.

* Checklist recommended by Agency for Healthcare Research and Quality (A HRQ) for cross-sectional/prevalence study quality: 4, 2.1 percent.

* Child Health Epidemiology Reference Group (C HERG): 3, 1.6 percent.

* Scottish Intercollegiate Guidelines Network (S IG N): 3, 1.6 percent.

* Critical Appraisal Skills Programme (C ASP): 3, 1.6 percent.

* Other: 20, 10.5 percent.

Navigate back to Table 2.

## References

[1] World Health Organization (2026) *Vaccines and immunization.* https://www.who.int/health-topics/vaccines-and-immunization#tab=tab_1 (accessed 1 January 2026).

[2] Lopalco PL and DeStefano F (2015) The complementary roles of phase 3 trials and post-licensure surveillance in the evaluation of new vaccines. Vaccine 33(13), 1541–1548. 10.1016/j.vaccine.2014.10.047 25444788 PMC4596394

[3] Metelli S and Chaimani A (2020) Challenges in meta-analyses with observational studies. Evidence Based Mental Health 23(2), 83–87. 10.1136/ebmental-2019-300129 32139442 PMC10231593

[4] Leis AM, et al. (2024) Evaluation of test-negative design estimates of influenza vaccine effectiveness in the context of multiple, co-circulating, vaccine preventable respiratory viruses. Vaccine 42(26), 126493. 10.1016/j.vaccine.2024.126493 39476473 PMC12291198

[5] Stuurman AL, et al. (2023) Investigating confounding in network‐based test‐negative design influenza vaccine effectiveness studies—Experience from the DRIVE project. Influenza and Other Respiratory Viruses 17(1), e13087. 10.1111/irv.13087 36550627 PMC9835455

[6] Amin AB, et al. (2024) Severity-dependent test-seeking behaviors and test-negative designs: Impact on estimated vaccine effectiveness and utility of analytic and design choices. American Journal of Epidemiology 194(7), 1855–1862. 10.1093/aje/kwae303 39191656 PMC12234220

[7] Davoodi, Z., et al. (2026) *Risk of Bias Tools for Vaccine Effectiveness Studies: A protocol for a scoping review.* https://osf.io/euz9b/overview (accessed 1 January 2026).

[8] Arksey H and O’Malley L (2005) Scoping studies: Towards a methodological framework. International Journal of Social Research Methodology 8, 19–32. 10.1080/1364557032000119616

[9] Levac D, Colquhoun H and O’Brien KK (2010) Scoping studies: Advancing the methodology. Implementation Science 5, 69. 10.1186/1748-5908-5-69 20854677 PMC2954944

[10] Tricco AC, et al. (2018) PRISMA extension for scoping reviews (PRISMA-ScR): Checklist and explanation. Annals of Internal Medicine 169(7), 467–473. 10.7326/M18-0850 30178033

[11] Page MJ, et al. (2021) The PRISMA 2020 statement: An updated guideline for reporting systematic reviews. BMJ 372, n71. 10.1136/bmj.n71 33782057 PMC8005924

[12] McGowan J, et al. (2016) PRESS peer review of electronic search strategies: 2015 guideline statement. Journal of Clinical Epidemiology 75, 40–46. 10.1016/j.jclinepi.2016.01.021 27005575

[13] Canada’s Drug Agency (2026) *Grey matters.* https://greymatters.cda-amc.ca/ (accessed 1 January 2026).

[14] Sullivan SG and Cowling BJ. (2015) “Crude vaccine effectiveness” is a misleading term in test-negative studies of influenza vaccine effectiveness. Epidemiology 26(5), e60. 10.1097/EDE.0000000000000343 26133018 PMC4522193

[15] Guyatt GH, et al. (2008) GRADE: An emerging consensus on rating quality of evidence and strength of recommendations. BMJ 336(7650), 924–926. 10.1136/bmj.39489.470347.AD 18436948 PMC2335261

[16] Walker N, et al. (2010) Standards for CHERG reviews of intervention effects on child survival. International Journal of Epidemiology 39(Suppl 1), i21–i31. 10.1093/ije/dyq036 20348122 PMC2845875

[17] Schünemann H, et al. (2013) *GRADE handbook for grading quality of evidence and strength of recommendations. The GRADE Working Group.* https://gdt.gradepro.org/app/handbook/handbook.html (accessed 5 August 2025).

[18] Wells GA, et al. (2026) *The Newcastle-Ottawa Scale (NOS) for assessing the quality of nonrandomised studies in meta-analyses.* https://ohri.ca/en/who-we-are/core-facilities-and-platforms/ottawa-methods-centre/newcastle-ottawa-scale (accessed 1 January 2026).

[19] Sterne JA, et al. (2016) ROBINS-I: A tool for assessing risk of bias in non-randomised studies of interventions. BMJ 355, 4919. 10.1136/bmj.i4919 PMC5062054 27733354

[20] Joanna Briggs Institute.(2026) *Critical appraisal tools.* https://jbi.global/critical-appraisal-tools (accessed 1 January 2026).

[21] Von Elm E, et al. (2008) The strengthening the reporting of observational studies in epidemiology (STROBE) statement: Guidelines for reporting observational studies. Journal of Clinical Epidemiology 61(4), 344–349. 10.1016/j.jclinepi.2007.11.008 18313558

[22] Higgins JPT and Green S (2011) *Cochrane handbook for systematic reviews of interventions.* Version 5.1.0. The Cochrane Collaboration. http://handbook-5-1.cochrane.org (accessed 1 January 2026).

[23] Hooijmans CR, et al. (2014) SYRCLE’S risk of bias tool for animal studies. BMC Medical Research Methodology 14(1), 43. 10.1186/1471-2288-14-43 24667063 PMC4230647

[24] Sun ZW, et al. (2021) Association of rotavirus vaccines with reduction in rotavirus gastroenteritis in children younger than 5 years: A systematic review and meta-analysis of randomized clinical trials and observational studies. JAMA Pediatrics 175(7), e210347. 10.1001/jamapediatrics.2021.0347 33970192 PMC8111566

[25] Ramsay LC, et al. (2019) The impact of repeated vaccination on influenza vaccine effectiveness: A systematic review and meta-analysis. BMC Medicine 17(1), 9. 10.1186/s12916-018-1239-8 30626399 PMC6327561

[26] Eliakim-Raz N, et al. (2013) Influenza vaccines in immunosuppressed adults with cancer. The Cochrane Database of Systematic Reviews 2013, CD008983. 10.1002/14651858.CD008983.pub2 24166741 PMC6457732

[27] Meggiolaro A, et al. (2022) Effectiveness of vaccination against SARS-CoV-2 infection in the pre-delta era: A systematic review and meta-analysis. Vaccine 10(2), 157. 10.3390/vaccines10020157 PMC8879968 35214615

[28] Zhu Y, Liu S and Zhang D (2022) Effectiveness of COVID-19 vaccine booster shot compared with non-booster: A meta-analysis. Vaccine 10(9), 1396. 10.3390/vaccines10091396 PMC9504142 36146474

[29] Zhang Z, et al. (2021) Two-dose varicella vaccine effectiveness in China: A meta-analysis and evidence quality assessment. BMC Infectious Diseases 21(1), 543. 10.1186/s12879-021-06217-1 34107891 PMC8188742

[30] Gupta C, et al. (2022) Effectiveness of the influenza vaccine at reducing adverse events in patients with heart failure: A systematic review and meta-analysis. Vaccine 40(25), 3433–3443. 10.1016/j.vaccine.2022.04.039 35562195

[31] Berild JD, et al. (2020) A systematic review of studies published between 2016 and 2019 on the effectiveness and efficacy of pneumococcal vaccination on pneumonia and invasive pneumococcal disease in an elderly population. Pathogens 9(4), 259. 10.3390/pathogens9040259 32260132 PMC7238108

[32] Viswanathan M, et al. (2012) Assessing the Risk of Bias of Individual Studies in Systematic Reviews of Health Care Interventions. Methods Guide for Effectiveness and Comparative Effectiveness Reviews [Internet]. Rockville (MD): Agency for Healthcare Research and Quality (US). 22479713

[33] Hughes SL, et al. (2020) The effect of time since measles vaccination and age at first dose on measles vaccine effectiveness - a systematic review. Vaccine 38(3), 460–469. 10.1016/j.vaccine.2019.10.090 31732326 PMC6970218

[34] Wang K, et al. (2022) Real-word effectiveness of global COVID-19 vaccines against SARS-CoV-2 variants: A systematic review and meta-analysis. Frontiers in Medicine 9. 10.3389/fmed.2022.820544 PMC9160927 35665358

[35] Wu N, et al. (2023) Long-term effectiveness of COVID-19 vaccines against infections, hospitalisations, and mortality in adults: Findings from a rapid living systematic evidence synthesis and meta-analysis up to December, 2022. The Lancet. Respiratory Medicine 11(5), 439–452. 10.1016/S2213-2600(23)00015-2 36780914 PMC9917454

[36] Bitterman R, et al. (2018) Influenza vaccines in immunosuppressed adults with cancer. The Cochrane Database of Systematic Reviews, CD008983. 10.1002/14651858.CD008983.pub3 29388675 PMC6491273

[37] Markowitz LE, et al. (2022) Human papillomavirus vaccine effectiveness by number of doses: Updated systematic review of data from national immunization programs. Vaccine 40(37), 5413–5432. 10.1016/j.vaccine.2022.06.065 35965239 PMC9768820

[38] Kandeil W, et al. (2020) A systematic review of the burden of pertussis disease in infants and the effectiveness of maternal immunization against pertussis. Expert Review of Vaccines 19(7), 621–638. 10.1080/14760584.2020.1791092 32772755

[39] Jackson C, et al. (2013) Effectiveness of Haemophilus influenzae type b vaccines administered according to various schedules: Systematic review and meta-analysis of observational data. The Pediatric Infectious Disease Journal 32(11), 1261–1269. 10.1097/INF.0b013e3182a14e57 23811746

[40] Abubakar I, et al. (2013) Systematic review and meta-analysis of the current evidence on the duration of protection by bacillus Calmette–Guérin vaccination against tuberculosis. Health Technology Assessment 17(37), 1–372. 10.3310/hta17370 PMC4781620 24021245

[41] Darvishian M, et al. (2014) After adjusting for bias in meta-analysis seasonal influenza vaccine remains effective in community-dwelling elderly. Journal of Clinical Epidemiology 67(7), 734–744. 10.1016/j.jclinepi.2014.02.009 24768004

[42] Remschmidt C, Wichmann O and Harder T (2015) Frequency and impact of confounding by indication and healthy vaccinee bias in observational studies assessing influenza vaccine effectiveness: A systematic review. BMC Infectious Diseases 15(1), 429. 10.1186/s12879-015-1154-y 26474974 PMC4609091

[43] Garland SM, et al. (2016) Impact and effectiveness of the quadrivalent human papillomavirus vaccine: A systematic review of 10 years of real-world experience. Clinical Infectious Diseases 63(4), 519–527. 10.1093/cid/ciw354 27230391 PMC4967609

[44] Caspard H, et al. (2017) Live-attenuated influenza vaccine effectiveness in children from 2009 to 2015-2016: A systematic review and meta-analysis. Open Forum Infectious Diseases 4(3), ofx111. 10.1093/ofid/ofx111 28852675 PMC5569992

[45] Drolet M, et al. (2019) Population-level impact and herd effects following the introduction of human papillomavirus vaccination programmes: Updated systematic review and meta-analysis. Lancet 394(10197), 497–509. 10.1016/S0140-6736(19)30298-3 31255301 PMC7316527

[46] Andani A, et al. (2022) One or two doses of hepatitis a vaccine in universal vaccination programs in children in 2020: A systematic review. Vaccine 40(2), 196–205. 10.1016/j.vaccine.2021.01.038 33526283

[47] Okoli GN, et al. (2021) Decline in seasonal influenza vaccine effectiveness with vaccination program maturation: A systematic review and meta-analysis. Open Forum Infectious Diseases 8(3), ofab069. 10.1093/ofid/ofab069 33738320 PMC7953658

[48] Luna EJDA, Gattás VL, Campos SRDSLDC (2014) Efetividade da estratégia Brasileira de vacinação contra influenza: Uma revisão sistemática. Epidemiologia e Serviços de Saúde 23, 559–576

[49] Markowitz LE, et al. (2018) Human papillomavirus vaccine effectiveness by number of doses: Systematic review of data from national immunization programs. Vaccine 36(32), 4806–4815. 10.1016/j.vaccine.2018.01.057 29802000

[50] Teerawattananon Y, et al. (2022) A systematic review of methodological approaches for evaluating real-world effectiveness of COVID-19 vaccines: Advising resource-constrained settings. PLoS One 17(1), e0261930. 10.1371/journal.pone.0261930 35015761 PMC8752025

[51] Mésidor M, et al. (2024) Test negative design for vaccine effectiveness estimation in the context of the COVID-19 pandemic: A systematic methodology review. Vaccine 42(5), 995–1003. 10.1016/j.vaccine.2023.12.013 38072756

[52] Downs SH and Black N (1998) The feasibility of creating a checklist for the assessment of the methodological quality both of randomised and non-randomised studies of health care interventions. Journal of Epidemiology and Community Health 52(6), 377–384. 10.1136/jech.52.6.377 9764259 PMC1756728

[53] Lu CY (2009) Observational studies: A review of study designs, challenges and strategies to reduce confounding. International Journal of Clinical Practice 63(5), 691–697. 10.1111/j.1742-1241.2009.02056.x 19392919

[54] Haddaway NR, et al. (2022) *PRISMA2020* : An R package and shiny app for producing PRISMA 2020‐compliant flow diagrams, with interactivity for optimised digital transparency and open synthesis. Campbell Systematic Reviews 18(2), e1230. 10.1002/cl2.1230 36911350 PMC8958186

[55] Fulton TR, et al. (2016) Protective effect of contemporary pertussis vaccines: A systematic review and meta-analysis. Clinical Infectious Diseases 62(9), 1100–1110. 10.1093/cid/ciw051 26908803 PMC4826451

