# Supplementary material for: Risk-of-bias assessment of vaccine effectiveness studies: a scoping review of systematic reviews
Source: Epidemiol Infect. 2026 Jun 19;154:e95. doi: 10.1017/S0950268826101794 (PMC13366358; doi:10.1017/S0950268826101794)
Supplement: Davoodi et al. supplementary material [file S0950268826101794sup001.zip › S0950268826101794sup002.pdf]

APPENDIX B: PRESS GUIDELINE 2015— SEARCH SUBMISSION & PEER REVIEW ASSESSMENT

Reference: McGowan J, Sampson M, Salzwedel DM, Cogo E, Foerster V, Lefebvre C. PRESS Peer Review of Electronic Search Strategies: 2015 guideline statement. *J Clin Epidemiol* 2016;75:40-6. Available: http://www.jclinepi.com/article/S0895-4356(16)00058-5/pdf.

Search submission: This section to be filled in by the searcher

Searcher: Becky Skidmore Email: becky.skidmore.rls@gmail.com

Date submitted: 2023 May 11 Date requested by: 2023 May 13 PM

1. Systematic Review Title

Risk of Bias Tools for Vaccine Effectiveness Studies: A protocol for a scoping review

2. This search strategy is …

X	My PRIMARY (core) database strategy — First time submitting a strategy for search question and database
	My PRIMARY (core) strategy — Follow-up review NOT the first time submitting a strategy for search question and database. If this is a response to peer review, itemize the changes made to the review suggestions
	SECONDARY search strategy— First time submitting a strategy for search question and database
	SECONDARY search strategy — NOT the first time submitting a strategy for search question and database. If this is a response to peer review, itemize the changes made to the review suggestions

3. Database (e.g., MEDLINE, CINAHL)

MEDLINE

4. Interface (e.g., Ovid, EbscoHost…)

Ovid

5. Research Question (Describe the purpose of the search) *[mandatory]*

KQ1. What are the existing tools used to assess risk of bias in vaccine effectiveness studies?

KQ2. What risk of bias concepts or sources are considered to be of particular concern for vaccine effectiveness studies?

6. PICO Format Outline the PICOs for your question — i.e., Patient, Intervention, Comparison, Outcome, and Study Design — as applicable

**P**	Vaccine Effectiveness Studies
**I / Exposure**	
**C**	
**O**	
**S**	Systematic Reviews

7. Inclusion Criteria (List criteria such as age groups, study designs, etc., to be included) *[optional]*

Date limits 2013-present

9. Exclusion Criteria (List criteria such as study designs, date limits, etc., to be excluded)

10. Was a search filter applied? Yes

If YES, which one(s) (e.g., Cochrane RCT filter, PubMed Clinical Queries filter)? Provide the source if this is a published filter. *[mandatory if YES to previous question — textbox]*

SR filter derived from CADTH's

11. Notes or comments you feel would be useful for the peer reviewer *[optional]*

Not interested in efficacy. Interest is in RoB considerations/concepts/tools as would be found in context of relevant study designs, e.g., test-negative, Phase IV, real world, observational studies.

Not interested in specific vaccines so language is intentionally generic.

Want to stay away from including concepts of "immunogenicity" - the effectiveness of vaccines in producing an immune response.

This project is being done in multiple parts.

1 – Vaccine effectiveness studies reporting use of an RoB tool/concept (systematic reviews of observational studies)
2 – Methodological papers reporting the development or use of an RoB tool/concept (any study design)
3 – Articles that identify RoB concepts in vaccine effectiveness studies (e.g., guidance for evaluating RoB in vaccine effectiveness studies, articles or reporting guidance that identify sources of bias in vaccine effectiveness studies)

12. Please copy and paste your search strategy here, exactly as run, including the number of hits per line. [mandatory]

Database: Ovid MEDLINE(R) ALL <1946 to May 09, 2023>
Search Strategy:
----
1 Vaccine Efficacy/ [VACCINE EFFECTIVENESS PT 1 - NEW MESH 2022] (732)
2 exp Vaccines/ (275350)

3 exp Vaccination/ (108352)
4 vaccin*.ti,kw,kf. (242408)
5 (immunis* or immuniz*).ti,kw,kf. (48043)
6 (vaccin* or immunis* or immuniz*).ab. /freq=2 (253574)
7 or/2-6 [VACCINES/VACCINATION] (428686)
8 (vaccin* adj5 effective*).tw,kw,kf. (41952)
9 7 and 8 [VACCINE EFFECTIVENESS PT 2] (36759)
10 1 or 9 [VACCINE EFFECTIVENESS PTs 1 or 2] (37039)
11 Systematic Review.pt. (228025)
12 exp Systematic Reviews as Topic/ (10481)
13 Meta Analysis.pt. (180624)
14 exp Meta-Analysis as Topic/ (26934)
15 (meta-analy* or metanaly* or metaanaly* or met analy* or integrative research or integrative review* or integrative overview* or research integration or research overview* or collaborative review*).tw,kw,kf. (276187)
16 (systematic review* or systematic overview* or evidence-based review* or evidence-based overview* or (evidence adj3 (review* or overview*)) or evidence map* or meta-review* or meta-overview* or meta-synthes* or mapping review? or rapid review* or "review of reviews" or scoping review? or umbrella review? or technology assessment* or HTA or HTAs).tw,kw,kf. (365957)
17 exp Technology Assessment, Biomedical/ (12121)
18 (cochrane or health technology assessment or evidence report or systematic reviews).jw. (22499)
19 Network Meta-Analysis/ (4843)
20 (network adj (MA or MAs)).tw,kw,kf. (17)
21 (NMA or NMAs or MTC or MTCs or MAIC or MAICs).tw,kw,kf. (9352)
22 indirect* compar*.tw,kw,kf. (2772)
23 (indirect treatment* adj1 compar*).tw,kw,kf. (463)
24 (mixed treatment* adj1 compar*).tw,kw,kf. (524)
25 (multiple treatment* adj1 compar*).tw,kw,kf. (225)
26 (multi-treatment* adj1 compar*).tw,kw,kf. (3)
27 simultaneous* compar*.tw,kw,kf. (1299)
28 mixed comparison?.tw,kw,kf. (44)
29 or/11-28 [SR FILTER] (554924)
30 10 and 29 (1389)
31 limit 30 to yr="2013-current" (1108)

****

Peer review assessment: this section to be filled in by the reviewer

Reviewer: Kaitryn Campbell	Email:campbell.information.consulting@gmail.com	Date completed: 13 May 2023

Do you wish to be acknowledged? (If yes, the review team will be advised to add an acknowledgement to any publications related to this work). Yes please.

The suggested acknowledgement is "We thank Kaitryn Campbell, MLIS, MSc for peer review of the Medline search strategy."

1. TRANSLATION

A ---No revisions	X
B --- Revision(s) suggested	
C --- Revision(s) required	

If "B" or "C," please provide an explanation or example:

2. BOOLEAN AND PROXIMITY OPERATORS

A ---No revisions	X
B --- Revision(s) suggested	
C --- Revision(s) required	

If “B” or “C,” please provide an explanation or example:

3. SUBJECT HEADINGS

A ---No revisions	X
B --- Revision(s) suggested	
C --- Revision(s) required	

If “B” or “C,” please provide an explanation or example:

4. TEXT WORD SEARCHING

A ---No revisions	
B --- Revision(s)suggested	X
C --- Revision(s) required	

If “B” or “C,” please provide an explanation or example:

Line 8, suggest consider adding: efficacies OR efficacious* OR efficacy

5. SPELLING, SYNTAX, AND LINE NUMBERS

A ---No revisions	X
B --- Revision(s)suggested	
C --- Revision(s) required	

If “B” or “C,” please provide an explanation or example:

6. LIMITS AND FILTERS

A ---No revisions	X
B --- Revision(s) suggested	
C --- Revision(s) required	

If “B” or “C,” please provide an explanation or example:

OVERALL EVALUATION (Note: If one or more "revision required" is noted above, the response below must be "revisions required".)

A ---No revisions	
B --- Revision(s) suggested	X
C --- Revision(s) required	

Additional comments:

Nicely done. No errors or omissions detected. I've made a few keyword suggestions.
