# Supplementary material for: Risk-of-bias assessment of vaccine effectiveness studies: a scoping review of systematic reviews
Source: Epidemiol Infect. 2026 Jun 19;154:e95. doi: 10.1017/S0950268826101794 (PMC13366358; doi:10.1017/S0950268826101794)
Supplement: Davoodi et al. supplementary material [file S0950268826101794sup001.zip › S0950268826101794sup003.pdf]

APPENDIX C. THE SEARCH STRATEGY

Embase Classic+Embase <1947 to 2023 May 16>, Ovid MEDLINE(R) ALL <1946 to May 16, 2023>

1 Vaccine Efficacy/ [VACCINE EFFECTIVENESS PT 1 - NEW MESH 2022] (84708)

2 exp Vaccines/ (727189)

3 exp Vaccination/ (368775)

4 vaccin*.ti,kw,kf. (551347)

5 (immunis* or immuniz*).ti,kw,kf. (108023)

6 (vaccin* or immunis* or immuniz*).ab. /freq=2 (583496)

7 or/2-6 [VACCINES/VACCINATION] (1071734)

8 (vaccin* adj5 (effective* or efficacies or efficacious* or efficacy)).tw,kw,kf. (141852)

9 7 and 8 [VACCINE EFFECTIVENESS PT 2] (129315)

10 1 or 9 [VACCINE EFFECTIVENESS PTs 1 or 2] (201211)

11 Systematic Review.pt. (228631)

12 exp Systematic Reviews as Topic/ (42064)

13 Meta Analysis.pt. (181011)

14 exp Meta-Analysis as Topic/ (79750)

15 (meta-analy* or metanaly* or metaanaly* or met analy* or integrative research or integrative review* or integrative overview* or research integration or research overview* or collaborative review*).tw,kw,kf. (643952)

16 (systematic review* or systematic overview* or evidence-based review* or evidence-based overview* or (evidence adj3 (review* or overview*)) or evidence map* or meta-review* or meta-overview* or meta-synthes* or mapping review? or rapid review* or "review of reviews" or scoping review? or umbrella review? or technology assessment* or HTA or HTAs).tw,kw,kf. (826777)

17 exp Technology Assessment, Biomedical/ (29227)

18 (cochrane or health technology assessment or evidence report or systematic reviews).jw. (56589)

19 Network Meta-Analysis/ (12647)

20 (network adj (MA or MAs)).tw,kw,kf. (47)

21 (NMA or NMAs or MTC or MTCs or MAIC or MAICs).tw,kw,kf. (24419)

22 indirect* compar*.tw,kw,kf. (8041)

23 (indirect treatment* adj1 compar*).tw,kw,kf. (1645)

24 (mixed treatment* adj1 compar*).tw,kw,kf. (1533)

25 (multiple treatment* adj1 compar*).tw,kw,kf. (524)

26 (multi-treatment* adj1 compar*).tw,kw,kf. (16)

27 simultaneous* compar*.tw,kw,kf. (2923)

28 mixed comparison?.tw,kw,kf. (105)

29 or/11-28 [SR FILTER] (1285272)

30 10 and 29 (5411)

31 limit 30 to yr="2013-current" (4304)

32 31 use medall [MEDLINE RECORDS] (1529)

33 exp vaccine/ (727189)

34 exp vaccination/ (368775)

35 vaccin*.ti,kw,kf. (551347)

36 (immunis* or immuniz*).ti,kw,kf. (108023)

37 (vaccin* or immunis* or immuniz*).ab. /freq=2 (583496)

38 or/33-37 [VACCINES/VACCINATION] (1071734)

39 (vaccin* adj5 (effective* or efficacies or efficacious* or efficacy)).tw,kw,kf. (141852)

40 38 and 39 [VACCINE EFFECTIVENESS PT 2] (129315)

41 "systematic review"/ (663727)

42 "systematic review (topic)"/ (31533)

43 meta analysis/ (473497)

44 "meta analysis (topic)"/ (52790)

45 (meta-analy* or metanaly* or metaanaly* or met analy* or integrative research or integrative review* or integrative overview* or research integration or research overview* or collaborative review*).tw,kw,kf. (643952)

46 (systematic review* or systematic overview* or evidence-based review* or evidence-based overview* or (evidence adj3 (review* or overview*)) or evidence map* or meta-review* or meta-overview* or meta-synthes* or mapping review? or rapid review* or "review of reviews" or scoping review? or umbrella review? or technology assessment* or HTA or HTAs).tw,kw,kf. (826777)

47 biomedical technology assessment/ (28055)

48 (cochrane or health technology assessment or evidence report or systematic reviews).jw. (56589)

49 network meta-analysis/ (12647)

50 (network adj (MA or MAs)).tw,kw,kf. (47)

51 (NMA or NMAs or MTC or MTCs or MAIC or MAICs).tw,kw,kf. (24419)

52 indirect* compar*.tw,kw,kf. (8041)

53 (indirect treatment* adj1 compar*).tw,kw,kf. (1645)

54 (mixed treatment* adj1 compar*).tw,kw,kf. (1533)

55 (multiple treatment* adj1 compar*).tw,kw,kf. (524)

56 (multi-treatment* adj1 compar*).tw,kw,kf. (16)

57 simultaneous* compar*.tw,kw,kf. (2923)

58 mixed comparison?.tw,kw,kf. (105)

59 or/41-58 [SR FILTER] (1401254)

60 40 and 59 (4938)

61 limit 60 to yr="2013-current" (3913)

62 61 use emczd [EMBASE RECORDS] (2388)

63 32 or 62 [BOTH DATABASES] (3917)

64 remove duplicates from 63 (2412) [TOTAL UNIQUE RECORDS]

65 64 use medall [MEDLINE UNIQUE RECORDS] (1503)

66 64 use emczd [EMBASE UNIQUE RECORDS] (909)

Web of Science

Set #	Search Query	Results
1	TI=(vaccin*) OR AK=(vaccin*)	281551
2	TI=(immunis* or immuniz*) OR AK=(immunis* or immuniz*)	55425
3	#2 OR #1	317969
4	vaccin* NEAR/5 (effective* or efficacies or efficacious* or efficacy) (Title) OR vaccin* NEAR/5 (effective* or efficacies or efficacious* or efficacy) (Abstract) OR vaccin* NEAR/5 (effective* or efficacies or efficacious* or efficacy) (Author Keywords)	64674
5	#3 AND #4	49181
6	"meta-analysis" or "meta-analyses" or "meta-analytic" or "meta-analytical" or metanaly* or metaanaly* or "met analysis" or "met analyses" or "met analytic" or "met analytical" or "integrative research" or "integrative review" or "integrative reviews" or "integrative overview" or "integrative overviews" or "research integration" or "research overview" or "research overviews" or "collaborative review" or "collaborative reviews" (Title) OR "meta-analysis" or "meta-analyses" or "meta-analytic" or "meta-analytical" or metanaly* or metaanaly* or "met analysis" or "met analyses" or "met analytic" or "met analytical" or "integrative research" or "integrative review" or "integrative reviews" or "integrative overview" or "integrative overviews" or "research integration" or "research overview" or "research overviews" or "collaborative review" or "collaborative reviews" (Abstract) OR	324863

	"meta-analysis" or "meta-analyses" or "meta-analytic" or "meta-analytical" or metanaly* or metaanaly* or "met analysis" or "met analyses" or "met analytic" or "met analytical" or "integrative research" or "integrative review" or "integrative reviews" or "integrative overview" or "integrative overviews" or "research integration" or "research overview" or "research overviews" or "collaborative review" or "collaborative reviews" (Author Keywords)	
7	"systematic review" or "systematic reviews" or "systematic overview" or "systematic overviews" or "evidence-based review" or "evidence-based reviews" or "evidence-based overview" or "evidence-based overviews" or (evidence NEAR/3 (review* or overview*)) or "evidence map" or "evidence maps" or "evidence mapping" or "meta-review" or "meta-reviews" or "meta-overview" or "meta-overviews" or "meta-synthesis" or "meta-syntheses" or "mapping review" or "mapping reviews" or "rapid review" or "rapid reviews" or "review of reviews" or "scoping review" or "scoping reviews" or "umbrella review" or "umbrella reviews" or "technology assessment" or "technology assessments" or HTA or HTAs (Title) OR "systematic review" or "systematic reviews" or "systematic overview" or "systematic overviews" or "evidence-based review" or "evidence-based reviews" or "evidence-based overview" or "evidence-based overviews" or (evidence NEAR/3 (review* or overview*)) or "evidence map" or "evidence maps" or "evidence mapping" or "meta-review" or "meta-reviews" or "meta-overview" or "meta-overviews" or "meta-synthesis" or "meta-syntheses" or "mapping review" or "mapping reviews" or "rapid review" or "rapid reviews" or "review of reviews" or "scoping review" or "scoping reviews" or "umbrella review" or "umbrella reviews" or "technology assessment" or "technology assessments" or HTA or HTAs (Abstract) OR "systematic review" or "systematic reviews" or "systematic overview" or "systematic overviews" or "evidence-based review" or "evidence-based reviews" or "evidence-based overview" or "evidence-based overviews" or (evidence NEAR/3 (review* or overview*)) or "evidence map" or "evidence maps" or "evidence mapping" or "meta-review" or "meta-reviews" or "meta-overview" or "meta-overviews" or "meta-synthesis" or "meta-syntheses" or "mapping review" or "mapping reviews" or "rapid review" or "rapid reviews" or "review of reviews" or "scoping review" or "scoping reviews" or "umbrella review" or "umbrella reviews" or "technology assessment" or "technology assessments" or HTA or HTAs (Author Keywords)	434585
8	network NEAR/0 (MA or MAs) (Title) OR network NEAR/0 (MA or MAs) (Abstract) OR network NEAR/0 (MA or MAs) (Author Keywords)	158
9	NMA or NMAs or MTC or MTCs or MAIC or MAICs (Title) OR NMA or NMAs or MTC or MTCs or MAIC or MAICs (Abstract) OR NMA or NMAs or MTC or MTCs or MAIC or MAICs (Author Keywords)	13463
10	indirect* NEAR/0 compar* (Title) OR indirect* NEAR/0 compar* (Abstract) OR indirect* NEAR/0 compar* (Author Keywords)	3533
11	("indirect treatment" or "indirect treatments") NEAR/1 compar* (Title) OR ("indirect treatment" or "indirect treatments") NEAR/1 compar* (Abstract) OR ("indirect treatment" or "indirect treatments") NEAR/1 compar* (Author Keywords)	640

12	("mixed treatment" or "indirect treatments") NEAR/1 compar* (Title) OR ("mixed treatment" or "indirect treatments") NEAR/1 compar* (Abstract) OR ("mixed treatment" or "indirect treatments") NEAR/1 compar* (Author Keywords)	660
13	("multiple treatment" or "indirect treatments") NEAR/1 compar* (Title) OR ("multiple treatment" or "indirect treatments") NEAR/1 compar* (Abstract) OR ("multiple treatment" or "indirect treatments") NEAR/1 compar* (Author Keywords)	199
14	("multi-treatment" or "indirect treatments") NEAR/1 compar* (Title) OR ("multi-treatment" or "indirect treatments") NEAR/1 compar* (Abstract) OR ("multi-treatment" or "indirect treatments") NEAR/1 compar* (Author Keywords)	8
15	simultaneous* NEAR/0 compar* (Title) OR simultaneous* NEAR/0 compar* (Abstract) OR simultaneous* NEAR/0 compar* (Author Keywords)	3181
16	"mixed comparison" or "mixed comparisons" (Title) OR "mixed comparison" or "mixed comparisons" (Abstract) OR "mixed comparison" or "mixed comparisons" (Author Keywords)	52
17	#16 OR #15 OR #14 OR #13 OR #12 OR #11 OR #10 OR #9 OR #8 OR #7 OR #6	619924
18	#17 AND #5	1637
19	#17 AND #5 and 2023 or 2022 or 2021 or 2020 or 2019 or 2018 or 2017 or 2016 or 2015 or 2014 or 2013 (Publication Years)	1367
