# Supplementary material for: Risk-of-bias assessment of vaccine effectiveness studies: a scoping review of systematic reviews
Source: Epidemiol Infect. 2026 Jun 19;154:e95. doi: 10.1017/S0950268826101794 (PMC13366358; doi:10.1017/S0950268826101794)
Supplement: Davoodi et al. supplementary material [file S0950268826101794sup001.zip › S0950268826101794sup004.pdf]

APPENDIX D. GREY LITERATURE SEARCH STRATEGY

Suggested search terms: vaccine effectiveness; vaccine effectiveness risk of bias; vaccine risk of bias

Organization	Website Link
**Relevant websites suggested by co-authors**	
Coalition for Epidemic Preparedness Innovations (CEPI)	https://cepi.net/
NACI	https://www.canada.ca/en/public-health/services/immunization/national-advisory-committee-on-immunization-naci.html
View Hub	https://view-hub.org/
Vaccine Alliance	https://www.gavi.org/
**Selected relevant CADTH sources**	
**COVID-19 Grey Literature Sources**	
Joanna Briggs Institute (JBI) - COVID-19 Special Collection	https://joannabriggs.org/
ECRI-COVID-19 Resource Center	https://www.ecri.org/coronavirus-covid-19-outbreak-preparedness-center/
Centre for Evidence-Based Medicine — Oxford COVID-19 Evidence Service	https://www.cebm.net/oxford-covid-19-evidence-service/
National Institute for Health and Care Excellence - Coronavirus (COVID-19)	https://www.nice.org.uk/guidance/conditions-and-diseases/respiratory-conditions/covid19
Institut national d'excellence en santé et en services sociaux - COVID-19	https://www.inesss.qc.ca/covid-19.html
CADTH - CADTH and COVID-19	https://covid.cadth.ca/
McMaster University — COVID-19 Rapid Evidence Reviews	https://www.nccmt.ca/covid-19/covid-19-evidence-reviews

Health Technology Wales — Coronavirus (COVID-19)	https://www.healthtechnology.wales/covid-19/
Penn Medicine — Penn Medicine COVID-19 Guidance Summaries	http://www.uphs.upenn.edu/cep/COVID/indexCOVID.html
National COVID-19 Clinical Evidence Taskforce — Living Guidelines	https://covid19evidence.net.au/
Public Health Agency of Canada - Coronavirus disease (COVID-19)	https://www.canada.ca/en/public-health/services/diseases/coronavirus-disease-covid-19.html
Canadian Medical Protective Association - COVID-19 Hub	https://www.cmpa-acpm.ca/en/covid19
Canadian Medical Association - CMA Update: COVID-19	https://www.cma.ca/cma-update-coronavirus
CSA Group-Canadian Standards Association - COVID-19 Response Standards & Handbooks	https://www.csagroup.org/news/covid-19-response-standards-handbooks/
BC Centre for Disease Control - COVID-19 Care	http://www.bccdc.ca/health-professionals/clinical-resources/covid-19-care
Shared Health Manitoba - Provincial COVID-19 resources for health-care providers and staff	https://sharedhealthmb.ca/covid19/providers/
Government of Newfoundland and Labrador - COVID-19 Information for Health Professionals	https://www.gov.nl.ca/covid-19/for-health-professionals-2/
Public Health Ontario - Coronavirus Disease 2019 (COVID-19)	https://www.publichealthontario.ca/en/diseases-and-conditions/infectious-diseases/respiratory-diseases/novel-coronavirus
Ontario Ministry of Health and Ministry of Long-Term Care — COVID-19 Guidance for the Health Sector	http://www.health.gov.on.ca/en/pro/programs/publichealth/coronavirus/2019_guidance.aspx

Medical Society of Prince Edward Island - Health PEI Resources	https://mspei.org/covid/clinical-resources/
Collège des médecins du Québec — Suivez le fil d'actualité du Collège: COVID-19	http://www.cmq.org/page/fr/covid-19-suivez-le-fil-de-l-actualite-du-college.aspx
Saskatchewan Medical Association - COVID-19: Information and resources	https://www.sma.sk.ca/resources/70/covid-19-info-for-physicians.html
Centre d'expertise et de référence en santé publique — Publications	https://www.inspq.qc.ca/publications/notice.asp?E=p&NumPublication=653
Alberta Health Services — Scientific Advisory Group COVID-19 Recommendations	https://www.albertahealthservices.ca/topics/Page17074.aspx
Centers for Disease Control and Prevention — Healthcare Workers: Information on COVID-19	https://www.cdc.gov/coronavirus/2019-nCoV/hcp/index.html
ISDA–Infection Diseases Society of America — COVID-19 Real-Time Learning Network	https://www.idsociety.org/public-health/COVID-19-Resource-Center/
CIDRAP–Center for Infectious Disease Research and Policy — COVID-19 Resource Center	http://www.cidrap.umn.edu/infectious-disease-topics/covid-19
U.S. Food & Drug Administration — Coronavirus Disease 2019 (COVID-19)	https://www.fda.gov/emergency-preparedness-and-response/counterterrorism-and-emerging-threats/coronavirus-disease-2019-covid-19
U.S. Food & Drug Administration — COVID-19-Related Guidance Documents for Industry, FDA Staff, and Other Stakeholders	https://www.fda.gov/emergency-preparedness-and-response/coronavirus-disease-2019-covid-19/covid-19-related-guidance-documents-industry-fda-staff-and-other-stakeholders
APIC–Association for Professionals in Infection Control and Epidemiology — COVID-19	https://apic.org/covid19/
United States Department of Labor — Occupational Safety and Health Administration: COVID-19	https://www.osha.gov/SLTC/covid-19/standards.html

Government UK - Coronavirus (COVID-19): guidance	https://www.gov.uk/government/collections/coronavirus-covid-19-list-of-guidance
Public Health England - Finding the evidence: Coronavirus	https://phelibrary.koha-ptfs.co.uk/coronavirusinformation/
NHS England and NHS Improvement — Coronavirus guidance for clinicians and NHS managers	https://www.england.nhs.uk/coronavirus/
MRC Centre for Global Infectious Disease Analysis — COVID-19 reports	https://www.imperial.ac.uk/mrc-global-infectious-disease-analysis/covid-19/
Government of South Australia — Coronavirus Disease 2019 (COVID-19): Information for health professionals	https://www.sahealth.sa.gov.au/wps/wcm/connect/public+content/sa+health+internet/clinical+resources/clinical+programs+and+practice+guidelines/infectious+disease+control/coronavirus+disease+2019+information+for+health+professionals/novel+coronavirus+%282019-ncov%29+infection+information+for+health+professionals
Government of Western Australia Department of Health — COVID-19 information for health professionals	https://ww2.health.wa.gov.au/Articles/A_E/Coronavirus/COVID19-information-for-health-professionals
ANZICS–Australian and New Zealand Intensive Care Society — ANZICS COVID-19 Guidelines	https://www.anzics.com.au/coronavirus-guidelines/
Ministry of Health – Manatū Hauora — COVID-19: Information for health professionals	https://www.health.govt.nz/our-work/diseases-and-conditions/covid-19-novel-coronavirus/covid-19-novel-coronavirus-information-specific-audiences/covid-19-novel-coronavirus-resources-health-professionals
NSW–Government — COVID-19 (Coronavirus)	https://www.health.nsw.gov.au/Infectious/diseases/Pages/coronavirus-professionals.aspx
World Health Organization — Country & Technical Guidance - Coronavirus disease (COVID-19)	https://www.who.int/emergencies/diseases/novel-coronavirus-2019/technical-guidance-publications
European Centre for Disease Prevention and Control. COVID-19	https://www.ecdc.europa.eu/en/covid-19
Evidence Aid — Coronavirus (COVID-19): Information portal	https://www.evidenceaid.org/coronavirus-resources/
World Health Organization — Coronavirus disease (COVID-2019) situation reports	https://www.who.int/emergencies/diseases/novel-coronavirus-2019/situation-reports/

COVID-19 Canada Open Data Working Group — COVID-19 in Canada	https://art-bd.shinyapps.io/covid19canada/
Center for Systems Science and Engineering (CSSE) at Johns Hopkins University — COVID-19 Dashboard for cases globally	https://gisanddata.maps.arcgis.com/apps/opsdashboard/index.html#/bda7594740fd40299423467b48e9ecf6
Cochrane Library - Coronavirus (COVID-19)	https://www.cochranelibrary.com/covid-19
National Center for Biotechnology Information, U.S. National Library of Medicine — LitCOVID	https://www.ncbi.nlm.nih.gov/research/coronavirus/
U.S. National Library of Medicine – National Institutes of Health — Public Health Emergency COVID-19 Initiative	https://www.ncbi.nlm.nih.gov/pmc/about/covid-19/
Epistemonikos (L-OVE Summary) — COVID-19	https://app.iloveevidence.com/loves/5e6fdb9669c00e4ac072701d
World Health Organization. Global research on coronavirus disease (COVID-19)	https://www.who.int/emergencies/diseases/novel-coronavirus-2019/global-research-on-novel-coronavirus-2019-ncov
U.S. Department of Veteran Affairs Evidence Synthesis Program — COVID-19 Evidence Reviews	http://covid19reviews.org/index.cfm
medRxiv — COVID-19 SARS-CoV-2 preprints from medRxiv and bioRxiv	https://connect.medrxiv.org/relate/content/181
MLA–Medical Library Association — COVID-19 Resources for Medical Librarians & Other Health Information Professionals	https://www.mlanet.org/p/cm/ld/fid=1712
University of British Columbia — Coronavirus Disease 2019 (COVID-19)— for Beginners to Experts	https://wiki.ubc.ca/Coronavirus_Disease_2019_(COVID-19)%E2%80%94For_Beginners_to_Experts
EPPI-Centre — COVID-19: a living systematic map of the evidence	http://eppi.ioe.ac.uk/cms/Default.aspx?tabid=3765
McMaster University — COVID-End: COVID-19 Evidence Network to support Decision-making	https://www.mcmasterforum.org/networks/covid-end/

**Canadian health technology and assessment agencies**	
Alberta College of Family Physicians (ACFP)	https://acfp.ca/
Alberta Health and Wellness	http://www.health.alberta.ca/initiatives/AHTDP-reviews.html
CADTH	https://www.cadth.ca/search?keywords
Drug Safety and Effectiveness Network (DSEN)	http://www.cihr-irsc.gc.ca/e/49006.html
Health Quality Council of Alberta (HQCA)	http://hqca.ca/studies-and-reviews/completed-reviews/
Health Quality Ontario (HQO)	http://www.hqontario.ca/Evidence-to-Improve-Care/Health-Technology-Assessment
The Hospital for Sick Children (SickKids)	http://lab.research.sickkids.ca/task/reports-theses/
Institut national d'excellence en santé et en services sociaux (INESSS)	http://www.inesss.qc.ca/en/publications/publications.html
Institute of Health Economics (IHE)	http://www.ihe.ca/index.php?/publications
Manitoba Centre for Health Policy (MCHP)	http://mchp-appserv.cpe.umanitoba.ca/deliverablesList.html
McGill University Health Centre (MUHC)	https://muhc.ca/tau/page/tau-reports
NLCAHR : Newfoundland and Labrador Centre for Applied Health Research. Contextualized Health Research Synthesis Program (CHRSP)	http://www.nlcahr.mun.ca/CHRSP/CompletedCHRSP.php
Ottawa Hospital Research Institute (OHRI)	http://www.ohri.ca/ksgroup/Publications.aspx
Therapeutics Initiative	http://www.ti.ubc.ca/TherapeuticsLetter
Programs for Assessment of Technology in Health (Canada)	https://www.path-hta.ca/research
UBC Centre for Health Services and Policy Research	https://chspr.ubc.ca/publications/
**International health technology and assessment agencies**	
INAHTA	https://www.inahta.org/publications/

WHO Health Evidence Network (HEN)	https://www.euro.who.int/en/data-and-evidence/evidence-informed-policy-making/publications/by-keyword
Australia and New Zealand Horizon Scanning Network (ANZHSN)	http://www.horizonscanning.gov.au/internet/horizon/publishing.nsf/Content/technologies-assessed-lp-2
Australian Government Department of Health and Ageing. Medical Services Advisory Committee (MSAC)	http://www.msac.gov.au/internet/msac/publishing.nsf/Content/completed-assessments
Joanna Briggs Institute (JBI)	http://connect.jbiconnectplus.org/Search.aspx
Monash Health. Centre for Clinical Effectiveness (CCE).	http://monashhealth.org/health-professionals/cce/cce-publications/
National Prescribing Service. NPS RADAR	https://www.nps.org.au/radar
COAG Health Council	https://www.coaghealthcouncil.gov.au/AHMAC/Health-Technology-Reference-Group/Reports-and-Briefs
Institute of Technology Assessment (ITA)	http://www.oeaw.ac.at/ita/en/projects
Ludwig Boltzmann Institute of Health Technology Assessment (LBI)	http://eprints.hta.lbg.ac.at/
Belgian Health Care Knowledge Centre (KCE)	https://kce.fgov.be/en/all-reports
CEDIT Recommendations and Reports	http://cedit.aphp.fr/cedit-hta-agency/recommendations-reports/
French National Authority for Health (HAS)	http://www.has-sante.fr/jcms/c_946986/en/english-toutes-nos-publications-ligne-principale?portal=r_1457306
German Institute of Medical Documentation and Information (DIMDI)	https://www.dimdi.de/dynamic/en/homepage/
Health Information and Quality Authority	https://www.hiqa.ie/reports-and-publications/health-technology-assessments
Health Service Executive (Irish Health Repository)	https://www.lenus.ie/hse/
Health Council of the Netherlands	https://www.gezondheidsraad.nl/
National Health Care Institute Netherlands	https://english.zorginstituutnederland.nl/publications
Folkehelseinstituttet. Norwegian Institute of Public Health	https://www.fhi.no/en/publ/
Institute of Health Carlos III	https://publicaciones.isciii.es/
Healthcare Improvement Scotland	http://www.healthcareimprovementscotland.org/

NICE (NHS National Institute for Health and Care Excellence)	https://www.nice.org.uk/
NICE (Advice List)	https://www.nice.org.uk/guidance/published
NIHR	http://www.io.nihr.ac.uk/
NETSCC	https://www.journalslibrary.nihr.ac.uk/programmes/
National Health Service UK (NHS) England	https://www.england.nhs.uk/
AHRQ	https://www.ahrq.gov/cpi/about/index.html
Centers for Medicare and Medicaid Services (CMS)	https://www.cms.gov/About-CMS/About-CMS
Washington State Health Care Authority (HCA)	https://www.hca.wa.gov/about-hca
**Clinical Practice Guidelines**	
Alberta Medical Association	https://actt.albertadoctors.org/Pages/default.aspx
British Columbia Ministry of Health	https://www2.gov.bc.ca/gov/content/health/practitioner-professional-resources/bc-guidelines
Canadian Medical Association (CMA)	https://joulecma.ca/cpg/homepage
Canadian Partnership Against Cancer	https://www.partnershipagainstcancer.ca/tools/cancer-guidelines-database/
Canadian Standards Association (CSA)	https://store.csagroup.org/?cclcl=en_US
The College of Physicians and Surgeons of Ontario (CPSO)	https://www.cpso.on.ca/
Ontario Association of Medical Laboratories (OAML)	https://oaml.com/guidelines/
Public Health Agency of Canada (PHAC)	https://www.canada.ca/en/public-health/services/reports-publications/disease-prevention-control-guidelines.html
Registered Nurses' Association of Ontario (RNAO)	https://rnao.ca/bpg
Winnipeg Regional Health Authority (WRHA)	https://professionals.wrha.mb.ca/old/extranet/eipt/
American Association for Clinical Chemistry (AACC)	https://www.aacc.org/science-and-research/practice-guidelines
Centers for Disease Control and Prevention (CDC)	https://phgkb.cdc.gov/PHGKB/phgHome.action?action=home
The Regulation and Quality Improvement Authority (RQIA)	https://rqia.org.uk/what-we-do/rqia-s-funding-programme/guidelines/
Haute Autorité de santé/ French National Authority for Health (HAS)	https://www.has-sante.fr/jcms/c_6056/en/recherche-avancee?portlet=c_39085&search_antidot=&lang=en&typesf=guidelines

Institute for Clinical Systems Improvement (ICSI)	https://www.icsi.org/guidelines/
National Health and Medical Research Council (NHMRC)	https://www.clinicalguidelines.gov.au/
National Institute for Health and Care Excellence (NICE)	https://www.nice.org.uk/guidance/published
Scottish Intercollegiate Guidelines Network (SIGN)	https://www.sign.ac.uk/
**Databases (free)**	
Bandolier	http://www.bandolier.org.uk/booth/booths/smoking.html
LILACS	https://lilacs.bvsalud.org/en/
McMaster University, McMaster Health Forum	https://www.healthsystemsevidence.org/
NCBI	https://www.ncbi.nlm.nih.gov/books
TRIP Database	https://www.tripdatabase.com/
University of York (CRD)	https://www.crd.york.ac.uk/CRDWeb/
University of York. PROSPERO	https://www.crd.york.ac.uk/prospero/
US National Library of Medicine (NLM)	http://www.ncbi.nlm.nih.gov/pubmed
US National Library of Medicine & National Institutes of Health (NIH)	http://www.ncbi.nlm.nih.gov/pmc/
**Internet Search**	
Google (first 5 pages)	http://www.google.com
Google Scholar (first 5 pages)	https://scholar.google.com/
CMA	https://www.cma.ca/about-cma
