# Supplementary material for: Risk-of-bias assessment of vaccine effectiveness studies: a scoping review of systematic reviews
Source: Epidemiol Infect. 2026 Jun 19;154:e95. doi: 10.1017/S0950268826101794 (PMC13366358; doi:10.1017/S0950268826101794)
Supplement: Davoodi et al. supplementary material [file S0950268826101794sup001.zip › S0950268826101794sup005.pdf]

APPENDIX E. DATA EXTRACTION FORM

Study ID (Covidence ID)

Title

Lead author last name

Year of publication

Country in which the study conducted

- ☐ United States
- ☐ UK
- ☐ Canada
- ☐ Australia
- ☐ Other

Were there any conflicts of interest declared?

- ☐ Yes
- ☐ No
- ☐ Not reported

If yes, please specify the conflicts of interest:

Was the study funded?

- ☐ Yes
- ☐ No
- ☐ Not reported

If yes, please specify the funding source:

Notes

Additional general information regarding the study

Study methods

Aim of study

Authors' description of their study objective/research question

Years included in literature search

Study design(s) included in systematic review

Which study designs were included in the systematic review?

- ☐ Cohort design
- ☐ Cross-sectional design
- ☐ Case-control design
- ☐ Test-negative design
- ☐ Not reported

- ☐ Other

Number of observational studies included

Were RCTs also included in the systematic review?

- ☐ Yes
- ☐ No
- ☐ Unclear

Were there exclusions based on language?

- ☐ Yes
- ☐ No
- ☐ Unclear

If yes, please describe:

List additional exclusions

Study participants

What was the population of interest?

- ☐ Check all that apply
- ☐ General population
- ☐ Pediatric population
- ☐ Elderly population
- ☐ People with underlying chronic condition
- ☐ People with cancer
- ☐ Pregnant and/or lactating women
- ☐ Healthcare workers
- ☐ Occupational group other than HCWs
- ☐ Unclear
- ☐ Other

Interventions

Which vaccine(s) were assessed in the systematic review? Check all that apply

- ☐ COVID-19
- ☐ Seasonal influenza
- ☐ Pandemic influenza
- ☐ Pneumococcal disease
- ☐ Meningococcal disease
- ☐ Pertussis
- ☐ Rotavirus

- ☐ HPV
- ☐ Herpes zoster (Shingles)
- ☐ Varicella

Were RCTs also included in the systematic review?

- ☐ Yes
- ☐ No
- ☐ Unclear

Were there exclusions based on language?

- ☐ Yes
- ☐ No
- ☐ Unclear

If yes, please describe:

List additional exclusions

Study participants

What was the population of interest? Check all that apply

- ☐ General population
- ☐ Pediatric population
- ☐ Elderly population
- ☐ People with underlying chronic condition
- ☐ People with cancer
- ☐ Pregnant and/or lactating women
- ☐ Healthcare workers
- ☐ Occupational group other than HCWs
- ☐ Unclear
- ☐ Other

Interventions

Which vaccine(s) were assessed in the systematic review? Check all that apply

- ☐ COVID-19
- ☐ Seasonal influenza
- ☐ Pandemic influenza
- ☐ Pneumococcal disease
- ☐ Meningococcal disease
- ☐ Pertussis
- ☐ Rotavirus

- ☐ HPV
- ☐ Herpes zoster (Shingles)
- ☐ Varicella
- ☐ Measles/mumps/rubella (MMR)

Comparators

Which vaccine(s) was used as the comparator in the systematic review

- ☐ Unvaccinated
- ☐ COVID-19
- ☐ Seasonal influenza
- ☐ Pandemic influenza
- ☐ Pneumococcal disease
- ☐ Meningococcal disease
- ☐ Pertussis
- ☐ HPV
- ☐ Herpes zoster (shingles)
- ☐ Varicella
- ☐ Measles/mumps/rubella (MMR)
- ☐ Cholera
- ☐ Not reported
- ☐ Not applicable
- ☐ Other

Additional details regarding included vaccine(s)

(e.g., tetravalent, live-attenuated, specific SARS-CoV-2 vaccine)

Outcomes

Did the authors formally define vaccine effectiveness?

- ☐ Yes
- ☐ No

Definition of VE from a methodological standpoint Check all that apply

- ☐ (1-OR)*100
- ☐ (1-RR)*100
- ☐ (1-HR)*100
- ☐ Not reported
- ☐ Not applicable
- ☐ Other

Which outcomes were used to define vaccine effectiveness? Check all that apply

- ☐ Laboratory-confirmed infection
- ☐ Symptomatic disease
- ☐ Mortality
- ☐ Hospitalization
- ☐ Unclear
- ☐ Not reported
- ☐ Not applicable
- ☐ Other

Additional details regarding outcomes (e.g., all-cause mortality)

Were outcomes other than VE considered (e.g., adverse events)?

- ☐ Yes
- ☐ No
- ☐ Unclear

Risk of bias assessment

Did the authors use a previously developed RoB tool?

- ☐ Yes
- ☐ No

If yes, which RoB tool(s) was applied to observational studies in the review?

- ☐ Newcastle-Ottawa Scale
- ☐ ROBINS-I
- ☐ Downs and Black checklist
- ☐ GRADE
- ☐ JBI
- ☐ Not applicable
- ☐ Other

Did the authors make study-specific modifications to the RoB tool?

- ☐ Yes
- ☐ No
- ☐ Unclear
- ☐ Not applicable

If yes, which modifications were made? Check all that apply

- ☐ Dropping items
- ☐ Adding items
- ☐ Not applicable

- ☐ Alternative definitions for some items
- ☐ Not applicable
- ☐ Other

Additional details about how the RoB tool was applied

Reference provided for RoB tool

If the authors used a study-specific method to assess RoB, please describe the elements that were included (e.g., types of bias reviewed)

Additional notes
