# Supplementary material for: Risk-of-bias assessment of vaccine effectiveness studies: a scoping review of systematic reviews
Source: Epidemiol Infect. 2026 Jun 19;154:e95. doi: 10.1017/S0950268826101794 (PMC13366358; doi:10.1017/S0950268826101794)
Supplement: Davoodi et al. supplementary material [file S0950268826101794sup001.zip › S0950268826101794sup006.pdf]

APPENDIX F: SUPPLEMENTARY TABLES

Table S1. Summary of Characteristics of Included Studies (N=203)

Author (year of publication), Country	Included RCTs (yes/no)	Observational study designs studied, (n)	Population of Interest	Vaccine(s) assessed (Comparator)	Definitions of VE, effect estimate (if applicable)	VE-specific outcomes	Used a RoB tool (yes/no). If yes, name of RoB tool	Modified RoB tool (yes/no/unclear/ not applicable) If yes, see Table S3.
**Feikin (2022), Switzerland** [1]	Yes	Cohort design; Test-negative case-control, (n=15)	General population; Elderly population	COVID-19 (unvaccinated)	Narrative description	Laboratory-confirmed infection; Symptomatic disease; Hospitalization	Yes, ROBINS-I	No
**Sun (2021), China** [2]	Yes	Cohort design; Case-control design, (n=64)	Pediatric population	Rotavirus (unvaccinated)	(1-OR)*100; (1-RR)*100	Laboratory-confirmed infection; Hospitalization	Yes, NOS	Yes
**Ramsay (2019), Canada** [3]	No	Cohort design; Case-control design; Test-negative case-control; Surveillance; Secondary analysis, (n=20)	General population	Seasonal influenza (unvaccinated)	(1-OR)*100	Laboratory-confirmed infection	Yes, NOS	Yes
**Wang (2014), China** [4]	No	Cohort design; Case-control design, (n=32)	Pediatric population	Mumps (not reported)	(1-OR)*100; (1-RR)*100	Symptomatic disease	Yes, NOS	No
**Roy (2014), UK** [5]	No	Cohort design, (n=14)	Pediatric population	BCG (unvaccinated)	(1-RR)*100	Laboratory-confirmed infection	Yes, NOS	No
**Pal (2016), New Zealand** [6]	Yes	Matched case-control design, (n=1)	Pediatric population	Pertussis (unvaccinated)	Narrative description	Laboratory-confirmed infection	Yes, Other (PRISMA-guided checklist)	No
**McMillan (2014), Australia** [7]	Yes	Cohort design; Cross-sectional design; Case-control design, (n=13)	Pediatric population; Other (Pregnant and/or lactating women and infants)	Seasonal influenza; Pandemic influenza (unvaccinated)	Narrative description	Laboratory-confirmed infection; Symptomatic disease; Hospitalization	Yes, JBI	No
**Alqahtani (2015), Australia** [8]	No	Cohort design; Cross-sectional design; Case-control design, (n=17)	Other (Hajj pilgrims)	Any vaccine that reduces RTIs (influenza, pneumococcal, pertussis, diphtheria, Bacillus Calmette–Guérin (BCG), measles, mumps and rubella vaccine) (unvaccinated)	(1-RR)*100	Laboratory-confirmed infection; Symptomatic disease	Yes, Other (OCEBM tool)	Unclear
**Tormen (2022), Italy** [9]	No	Cohort design; Case-control design, (n=14)	Other (Pregnant and/or lactating women)	COVID-19 (unvaccinated)	(1-OR)*100	Laboratory-confirmed infection; Hospitalization	Yes, ROBINS-I; GRADE	No

Author (year of publication), Country	Included RCTs (yes/no)	Observational study designs studied, (n)	Population of Interest	Vaccine(s) assessed (Comparator)	Definitions of VE, effect estimate (if applicable)	VE-specific outcomes	Used a RoB tool (yes/no). If yes, name of RoB tool	Modified RoB tool (yes/no/unclear/ not applicable) If yes, see Table S3.
**Goodman (2022), USA** [10]	No	Not reported, (n=25)	Other (Recurrent respiratory papillomatosis patients, women with HPV-related anogenital disease, MSM, immunocompromised individuals, transgender and non-binary people, sex workers)	HPV (unvaccinated)	Narrative description	Symptomatic disease	Yes, NOS; GRADE	No
**Andersohn (2014), Germany** [11]	Yes	Cohort design; Case-control design; Prevalence studies; Ecological studies; Self-controlled designs, (n=8)	Pediatric population	Seasonal influenza (unvaccinated)	Narrative description	Symptomatic disease; Death; Hospitalization; Contact with HCP; Quality of Life	Yes, Other (GSWGTAHC checklist); SIGN	Unclear
**Moberley (2013), Australia** [12]	Yes	Cohort design; Case-control design, (n=7)	Elderly population; Other (those 18 years and older at higher risk of pneumococcal disease)	Pneumococcal disease (unvaccinated)	(1-OR)*100	Laboratory-confirmed infection	Yes, Cochrane RoB tool	Unclear
**Lukšić (2013), Croatia** [13]	Yes	Cohort design; Case-control design, (n=11)	Pediatric population	Seasonal influenza (unvaccinated)	(1-OR)*100; (1-RR)*100	Symptomatic disease	Yes, GRADE (as RoB assessment tool)	No
**Jackson (2013), UK** [14]	No	Cohort design; Case-control design; Screening method studies, (n=30)	General population	Haemophilus influenzae type B (unvaccinated; number of doses received)	(1-OR)*100; Other (VE= 1 - [(PCV* (1 - PPV)) / ((1 - PCV)*PPV)]) PCV = Proportion of Cases Vaccinated PPV = Proportion of Population Vaccinated	Death; Symptomatic disease	No (narrative description/self-developed methods)	N/A
**Breteler (2013), Switzerland** [15]	Yes	Cohort design; Case-control design, (n=16)	General population	Seasonal influenza; Pandemic influenza (unvaccinated)	Narrative description	Laboratory-confirmed infection; Mortality; Hospitalization; Symptomatic disease (otitis media, ILI, respiratory disease, outpatient visit, respiratory disease, exacerbations of asthma)	Yes, NOS	Unclear
**Abubakar (2013), UK** [16]	Yes	Cohort design; Cross-sectional design; Case-control design; Case population studies, (n=58)	General population	BCG (unvaccinated)	(1-RR)*100; (1-OR)*100	Symptomatic disease; Death	No (narrative description/self-developed methods)	N/A

Author (year of publication), Country	Included RCTs (yes/no)	Observational study designs studied, (n)	Population of Interest	Vaccine(s) assessed (Comparator)	Definitions of VE, effect estimate (if applicable)	VE-specific outcomes	Used a RoB tool (yes/no). If yes, name of RoB tool	Modified RoB tool (yes/no/unclear/ not applicable) If yes, see Table S3.
**Eliakim-Raz (2013), Israel** [17]	Yes	Cohort design; Case-control design, (n=3)	Other (people with cancer)	Seasonal influenza (unvaccinated)	(1-OR)*100	Laboratory-confirmed infection; Symptomatic disease; Death; Hospitalization	Yes, NOS; GRADE	Yes
**Davis (2013), USA** [18]	Yes	Case-control design; pre-post-vaccine introduction time periods, (n=16)	Pediatric population	Pneumococcal disease: Haemophilus influenzae type B (not reported)	(1-RR)*100	Death	Yes, NOS; CHERG	No
**Das (2013), Pakistan** [19]	Yes	Case-control design, (n=2)	Pediatric population	Cholera; Enterotoxigenic Escherichia coli (ETEC) (unvaccinated)	(1-RR)*100	Symptomatic disease; Death Hospitalization	Yes, CHERG (as RoB assessment tool)	Unclear
**O'Neill (2014), Canada** [20]	Yes	Cohort design, (n=5)	Other (Occupational group other than HCWs)	Q fever (unvaccinated)	Other (VE = 1 - [(c1 / N1) / ((c0 + 1) / (N0 + 1))], where VE = (1 - RR) * 100, RR = (c1 / N1) (risk in the vaccinated group) and the risk in the unvaccinated group is adjusted with a continuity correction (c0 + 1) / (N0 + 1))	Laboratory-confirmed infection; Symptomatic disease	Yes, Other (ROBANS)	Unclear
**Chan (2014), China** [21]	No	Cohort design; Case-control design, (n=11)	Elderly population	Seasonal influenza (unvaccinated)	(1-OR)*100; (1-RR)*100	Laboratory-confirmed infection; Mortality (due to pneumonia or influenza); Hospitalization; Symptomatic disease (clinically defined ILI, clinically defined pneumonia)	Yes, NOS	Yes
**Darvishian (2014), Netherlands** [22]	No	Cohort design, (n=14)	Elderly population	Seasonal influenza (unvaccinated)	(1-OR)*100	Laboratory-confirmed infection; Symptomatic disease; Death; Hospitalization	No (narrative description/self-developed methods)	N/A
**Remschmidt (2014), Germany** [23]	Yes	Cohort design, (n=3)	General population; Pediatric population; Other (People with underlying chronic condition); Other (Individuals with HIV infection)	Seasonal influenza (unvaccinated)	(1-RR)*100	Laboratory-confirmed infection; Symptomatic disease; Hospitalization	Yes, CASP; GRADE	No
**Remschmidt (2014), Germany** [24]	No	Cohort design, (n=5)	Other (People with underlying chronic condition)	Seasonal influenza (unvaccinated)	(1-OR)*100; (1-IRR)*100	Laboratory-confirmed infection; Symptomatic disease; Death; Hospitalization	Yes, CASP; GRADE	No

Author (year of publication), Country	Included RCTs (yes/no)	Observational study designs studied, (n)	Population of Interest	Vaccine(s) assessed (Comparator)	Definitions of VE, effect estimate (if applicable)	VE-specific outcomes	Used a RoB tool (yes/no). If yes, name of RoB tool	Modified RoB tool (yes/no/unclear/ not applicable) If yes, see Table S3.
**Machaira (2015), Greece** [25]	Yes	Cohort design, (n=5)	Pediatric population	Hepatitis B (HBIG recipients)	(1-OR)*100	Laboratory-confirmed infection; Mortality (all-cause)	Yes, NOS	No
**Li (2015), China** [26]	Yes	Cohort design; Case-control design, (n=18)	General population	Seasonal influenza (not reported)	(1-OR)*100; (1-RR)*100	Laboratory-confirmed infection; Hospitalization; Symptomatic disease	Yes, NOS	No
**Remschmidt (2015), Germany** [27]	No	Cohort design; Case-control design, (n=11)	Other (People with underlying chronic condition)	Seasonal influenza (unvaccinated)	(1-OR)*100	Laboratory-confirmed infection; Symptomatic disease; Death; Hospitalization	Yes, NOS; GRADE	No
**de Oliveira (2015), USA** [28]	No	Case-control design, (n=8)	Pediatric population	Rotavirus (hospitalized controls)	(1-OR)*100	Laboratory-confirmed infection; Symptomatic disease; Hospitalization	Yes, NOS	No
**Remschmidt (2015), Germany** [29]	No	Cohort design; Case-control (matched) design, (n=23)	General population; Elderly population; Other (People with underlying chronic condition); Other (Pregnant and/or lactating women)	Seasonal influenza (unvaccinated)	(1-OR)*100	Symptomatic disease; Death; Hospitalization	No (narrative description/self-developed methods)	N/A
**Hirve (2016), Switzerland** [30]	Yes	Cohort design; Case-control design, (n=18)	Pediatric population; Elderly population; Other (Pregnant and/or lactating women); Other (high risk individuals, healthy adults)	Seasonal influenza (unvaccinated)	Narrative description	Laboratory-confirmed infection; Symptomatic disease; Death; Hospitalization	Yes, NOS	No
**Kraicer-Melamed (2016), Canada** [31]	Yes	Cohort design; Case-control design; Ecological studies; Surveillance methods, (n=29)	Elderly population	Pneumococcal disease (unvaccinated)	Other (Broome method)	Symptomatic disease (community-acquired pneumonia, invasive pneumococcal disease)	Yes, Other (NACI guidelines for quality assessment)	Unclear
**Fulton (2016), USA** [32]	Yes	Cohort design; Case-control design; Screening study, (n=23)	Pediatric population	Pertussis (not reported)	Narrative description	Laboratory-confirmed infection; Symptomatic disease	Yes, CHERG (as RoB assessment tool)	No
**Garland (2016), Australia** [33]	Yes	Cohort design; Cross-sectional design; Case-control design, (n=29)	General population	HPV (unvaccinated)	Narrative description	Laboratory-confirmed infection; Symptomatic disease (genital warts, cervical lesions)	No (narrative description/self-developed methods)	N/A
**Lamberti (2016), USA** [34]	Yes	Not reported, (n=19)	Pediatric population	Rotavirus (not reported)	(1-OR)*100; (1-HR)*100	Symptomatic disease; Death; Hospitalization	Yes, CHERG (as RoB assessment tool)	Unclear

**Author (year of publication), Country**	**Included RCTs (yes/no)**	**Observational study designs studied, (n)**	**Population of Interest**	**Vaccine(s) assessed (Comparator)**	**Definitions of VE, effect estimate (if applicable)**	**VE-specific outcomes**	**Used a RoB tool (yes/no). If yes, name of RoB tool**	**Modified RoB tool (yes/no/unclear/ not applicable) If yes, see Table S3.**
**Casanova (2016), France** [35]	No	Cohort design; Case-control design, (n=7)	Other (People with underlying chronic condition)	Seasonal influenza (unvaccinated)	Other (Risk reduction)	Death; Hospitalization	Yes, Other (Gradation of the National Agency of accreditation and health assessment)	Unclear
**Santos (2016), Brazil** [36]	No	Cohort; Cross-sectional; Case-control design; Case series; Surveillance methods, (n=20)	Pediatric population	Rotavirus (unvaccinated)	(1-OR)*100	Laboratory-confirmed infection; Hospitalization	Yes, NOS	No
**Zhang (2016), China** [37]	Yes	Cohort design; Prospective study, retrospective, (n=5)	Elderly population	Seasonal influenza; Pneumococcal disease (unvaccinated)	(1-RR)*100	Symptomatic disease; Mortality	Yes, NOS	No
**Caspard (2017), USA** [38]	No	Cohort design; Case-control design; Test-negative design, (n=29)	Pediatric population	Seasonal influenza (unvaccinated)	(1-OR)*100; (1-RR)*100	Laboratory-confirmed infection	No (narrative description/self-developed methods)	N/A
**Willame (2018), Belgium** [39]	No	Cohort design; Case-control design; Test-negative design, (n=29)	Pediatric population	Rotavirus (unvaccinated)	(1-OR)*100	Laboratory-confirmed infection; Symptomatic disease	Yes, Other (CoCanCPG)	No
**Poudel (2019), USA** [40]	No	Cohort design, (n=8)	Other (People with underlying chronic condition); Other (Patients with heart failure)	Seasonal influenza (unvaccinated)	(1-HR)*100	Mortality; Hospitalization	Yes, NOS	No
**Domnich (2017), Italy** [41]	No	Cohort design; Case-control design, (n=11)	General population; Elderly Population (long-term care facilities)	Seasonal influenza (unvaccinated; recipients of other influenza vaccines)	(1-OR)*100; (1-RR)*100	Laboratory-confirmed infection; Symptomatic disease; Hospitalization	Yes, NOS	No
**Falkenhorst (2017), Germany** [42]	Yes	Cohort design; Case-control design; Case-case design, (n=13)	Elderly population; Other (People with underlying chronic condition)	Pneumococcal disease (unvaccinated)	(1-OR)*100	Symptomatic disease	Yes, NOS; GRADE	Unclear
**Lansbury (2017), UK** [43]	Yes	Cohort design; Case-control design; Test-negative design; Screening method studies, (n=36)	General population; Pediatric population	Pandemic influenza (unvaccinated)	(1-OR)*100	Laboratory-confirmed infection; Hospitalization	Yes, NOS; Cochrane RoB Tool	Unclear

Author (year of publication), Country	Included RCTs (yes/no)	Observational study designs studied, (n)	Population of Interest	Vaccine(s) assessed (Comparator)	Definitions of VE, effect estimate (if applicable)	VE-specific outcomes	Used a RoB tool (yes/no). If yes, name of RoB tool	Modified RoB tool (yes/no/unclear/ not applicable) If yes, see Table S3.
**Bekkat-Berkani (2017), USA** [44]	Yes	Cohort design; Self-controlled case series, (n=9)	Other (People with underlying chronic condition)	Seasonal influenza (unvaccinated)	Narrative description	Death; Hospitalization	Yes, SIGN	Unclear
**Tin Tin Htar (2017), Italy** [45]	No	Cohort design; Case-control design; Case-cohort, Self-controlled risk windows, (n=33)	General population	Pneumococcal disease (unvaccinated)	(1-OR)*100; (1-RR)*100; (1-HR)*100; (1-IRR)*100	Symptomatic disease	Yes, NOS	No
**Vasileiou (2017), UK** [46]	Yes	Cohort design; Case-control design, (n=15)	Other (People with underlying chronic condition)	Seasonal influenza (unvaccinated)	(1-OR)*100; (1-RR)*100	Laboratory-confirmed infection; Hospitalization	Yes, Other (EPHPP quality assessment tool); GRADE	No
**Bi (2017), USA** [47]	Yes	Cohort design; Case-control design; Case-cohort, (n=6)	General population	Cholera (unvaccinated)	(1-RR)*100	Laboratory-confirmed infection	Yes, NOS	No
**Hungerford (2017), UK** [48]	No	Cohort design; Case-control design, n=30	General population	Rotavirus (unvaccinated)	(1-OR)*100; (1-RR)*100	Contact with HCP	Yes, NOS	No
**Furuta (2017), Japan** [49]	Yes	Cohort design; Case-control design, n=13	Other (Pregnant and/or lactating women)	Pertussis (unvaccinated)	(1-OR)*100; (1-RR)*100	Laboratory-confirmed infection; Pregnancy-related outcomes (preterm deliveries)	Yes, ROBINS-I; GRADE	No
**Restivo (2018), Italy** [50]	No	Cohort design; Case-control design, n=38	Pediatric population; Elderly population; Other (People with underlying chronic condition); Other (Pregnant and/or lactating women); Other (Healthcare workers)	Seasonal influenza; Pandemic influenza (unvaccinated)	(1-OR)*100	Laboratory-confirmed infection; Death; Hospitalization	Yes, NOS; Cochrane RoB Tool	Unclear
**Zhu (2018), China** [51]	Yes	Cohort, computer system-based, n=23	Pediatric population	Varicella (not reported)	Narrative description	Symptomatic disease	Yes, NOS	Yes
**Young (2018), Singapore** [52]	No	Test-negative design, n=14	General population	Seasonal influenza (seasonal influenza)	(1-OR)*100	Laboratory-confirmed infection	Yes, GRADE (as RoB assessment tool)	No

Author (year of publication), Country	Included RCTs (yes/no)	Observational study designs studied, (n)	Population of Interest	Vaccine(s) assessed (Comparator)	Definitions of VE, effect estimate (if applicable)	VE-specific outcomes	Used a RoB tool (yes/no). If yes, name of RoB tool	Modified RoB tool (yes/no/unclear/ not applicable) If yes, see Table S3.
**Schwerdtle (2018), Australia** [53]	No	Case-control design; Case-cohort design, n=4	General population; Other (areas where cases reached epidemic threshold and cholera epidemic was declared)	Cholera (unvaccinated)	(1-OR)*100	Symptomatic disease	Yes, JBI	Unclear
**Yin (2018), China** [54]	Yes	Cohort design; Case-control design, n=12	Pediatric population	Varicella (varicella)	(1-OR)*100; (1-RR)*100	Laboratory-confirmed infection; Symptomatic disease	Yes, NOS	No
**Bitterman (2018), Israel** [55]	Yes	Cohort design; Case-control design, n=3	Other (people with cancer)	Seasonal influenza; Pandemic influenza (unvaccinated; placebo; or a different vaccine)	(1-OR)*100	Laboratory-confirmed infection; Symptomatic disease; Death; Hospitalization; Composite outcomes (hospitalizations for fever or acute respiratory infection, pneumonia requiring antibiotics, chemotherapy interruptions due to infection, necessity of antibiotics during hospitalization)	Yes, NOS; GRADE	Yes
**Dos Santos (2018), Belgium** [56]	Yes	Cohort design; Case-control design, n=9	Other (People with underlying chronic condition)	Seasonal influenza (unvaccinated)	Narrative description	Symptomatic disease; Death Hospitalization; Contact with HCP	Yes, SIGN	No
**Zimmerman (2018), Australia** [57]	Yes	Cohort design; Case-control design, n=8	General population; Pediatric population	Other: tuberculosis (BCG) (unvaccinated)	(1-OR)*100; (1-RR)*100	Laboratory-confirmed infection; Symptomatic disease	Yes, ROBINS-I	No
**Lee (2018), Canada** [58]	Yes	Cohort design, n=4	Elderly population	Seasonal influenza (seasonal influenza)	(1-OR)*100; Other (pooled relative VE (rVE))	Laboratory-confirmed infection; Symptomatic disease; Death; Hospitalization; Contact with HCP	Yes, Downs and Black checklist	Yes
**Bartoszko (2018), Canada** [59]	Yes	Cohort design; Case-control design; Test-negative design, n=34	General population; Pediatric population	Seasonal influenza; Pandemic influenza (seasonal influenza)	(1-OR)*100	Laboratory-confirmed infection	Yes, NOS; GRADE	No
**Markowitz (2018), Canada** [60]	No	Cohort design; Cross-sectional design; Case-control design, n=14	General population	HPV (unvaccinated)	Narrative description	Laboratory-confirmed infection; Symptomatic disease	No (narrative description/self-developed methods)	N/A

Author (year of publication), Country	Included RCTs (yes/no)	Observational study designs studied, (n)	Population of Interest	Vaccine(s) assessed (Comparator)	Definitions of VE, effect estimate (if applicable)	VE-specific outcomes	Used a RoB tool (yes/no). If yes, name of RoB tool	Modified RoB tool (yes/no/unclear/ not applicable) If yes, see Table S3.
**Chit (2018), Canada** [61]	No	Cohort design; Case-control design, n=7	Pediatric population	Pertussis (unvaccinated)	(1-RR)*100	Laboratory-confirmed infection; Symptomatic disease	Yes, Downs and Black checklist	Unclear
**Yin (2018), China** [62]	Yes	Cohort design; Case-cohort, n=9	Elderly population	Seasonal influenza; Pneumococcal disease (unvaccinated)	(1-RR)*100	Laboratory-confirmed infection; Symptomatic disease (classifications based on (ICD-10-AM, ICD-9-CM, ICD)); Death; Hospitalization;	Yes, NOS	No
**Harder (2018), Germany** [63]	Yes	Cohort design; Cross-sectional design, n=3	Other (Males only, no age restriction)	HPV (unvaccinated)	(1-RR)*100	Symptomatic disease (recurrent high grade anal intraepithelial neoplasia (AIN), anal condyloma, persisting oral infection)	Yes, ROBINS-I; GRADE	No
**Campbell (2018), UK** [64]	Yes	Cohort design; Non-randomized intervention study, n=7	Pediatric population; Other (infants following immunization in pregnancy)	Pertussis (unvaccinated)	Narrative description	Laboratory-confirmed infection; Hospitalization; Symptomatic disease	Yes, Cochrane RoB tool	Yes
**Tricco (2018), Canada** [65]	Yes	Cohort design; Case-control design; Non-randomized, n=5	Elderly population	Herpes zoster (Shingles) (placebo)	(1-OR)*100; (1-RR)*100	Symptomatic disease (herpes zoster ophthalmicus, postherpetic neuralgia)	Yes, NOS; Other (Cochrane EPOC RoB tool)	No
**Sings (2019), USA** [66]	No	Case-control design; Indirect cohort, n=8	Pediatric population	Pneumococcal disease (unvaccinated)	(1-OR)*100	Symptomatic disease	Yes, NOS	No
**Yakely (2019), USA** [67]	No	Cohort design, n=3	Other (MSM >25 years old, females 9-18 years, females 15-22 with evidence of sexual activity)	HPV (unvaccinated)	Narrative description	Symptomatic disease	No (narrative description/se lf-developed methods)	N/A
**Ngocho (2019), Tanzania** [68]	No	Laboratory surveillance (before and after vaccine), n=8	Pediatric population	Pneumococcal disease (other: before/after design)	(1-OR)*100; (1-RR)*100	Symptomatic disease	Yes, NOS; NHLBI tool	No
**Senderovich (2019), Canada** [69]	Yes	Cohort design; Case-control design, n=7	Elderly population (Long-term care facility residents)	Herpes zoster (Shingles) (unvaccinated)	Narrative description	Laboratory-confirmed infection	Yes, Cochrane RoB tool	Unclear

Author (year of publication), Country	Included RCTs (yes/no)	Observational study designs studied, (n)	Population of Interest	Vaccine(s) assessed (Comparator)	Definitions of VE, effect estimate (if applicable)	VE-specific outcomes	Used a RoB tool (yes/no). If yes, name of RoB tool	Modified RoB tool (yes/no/unclear/ not applicable) If yes, see Table S3.
**Adetokunboh (2019), South Africa** [70]	Yes	Cohort design; Cross-sectional design; Case-control design, n=9	Pediatric population; Other (HIV-infected in comparison with HIV-exposed or HIV-uninfected children aged <18 years)	Other: 'standard' vaccines (unvaccinated; other vaccines)	(1-OR)*100; (1-RR)*100	Laboratory-confirmed infection; Symptomatic disease	Yes, ROBINS-I; GRADE	Yes
**Doyon-Plourde (2019), Canada** [71]	No	Cohort design; Case-control design; Case-base, n=22	General population	Seasonal influenza (unvaccinated)	(1-OR)*100; (1-RR)*100; (1-HR)*100	Laboratory-confirmed infection; Hospitalization; Contact with HCP	Yes, ROBINS-I; GRADE	Yes
**Vardanjani (2019), Iran** [72]	Yes	Case-control design; Pre-post studies, n=7	Pediatric population; Other (pediatric population living with and without HIV)	Pneumococcal disease (unvaccinated)	(1-OR)*100; (1-RR)*100	Symptomatic disease	Yes, NOS	No
**Harmala (2019), UK** [73]	Yes	Cohort design, n=5	Other (People with underlying chronic condition)	Seasonal influenza (unvaccinated)	(1-RR)*100	Death; Hospitalization	Yes, NOS; GRADE	Unclear
**Nic Lochlainn (2019), Netherlands** [74]	Yes	Pre-post (within study comparisons), n=8	Pediatric population; Other (infants <9 months)	Other: first measles-containing vaccine (MCV1) (unvaccinated)	(1-RR)*100	Symptomatic disease	Yes, GRADE (as RoB assessment tool)	No
**Nic Lochlainn (2019), Netherlands** [75]	Yes	Not reported, n=2	Pediatric population; Other (infants <9 months)	Other: measles-containing vaccine (unvaccinated)	Narrative description	Symptomatic disease	Yes, GRADE (as RoB assessment tool)	No
**Friedman (2019), Canada** [76]	Yes	Cohort design, n=19	General population; Pediatric population; Elderly population	Seasonal influenza (unvaccinated)	(1-OR)*100; (1-RR)*100	Symptomatic disease; Death; Hospitalization; Contact with HCP	Yes, CASP	No
**Drolet (2019), Canada** [77]	No	Time-trend analysis, n=40	General population	HPV (unvaccinated)	(1-RR)*100	Laboratory-confirmed infection; Symptomatic disease	No (narrative description/self-developed methods)	N/A
**Lindsey (2019), Gambia** [78]	Yes	Case-control design; Cohort design, n=11	General population; Pediatric population; Elderly population	Seasonal influenza; Pandemic influenza (not reported)	(1-OR)*100	Laboratory-confirmed infection; Death; Hospitalization	Yes, GRADE (as RoB assessment tool)	No
**Tadount (2020), Canada** [79]	Yes	Test-negative design, n=6	Elderly population	Seasonal influenza (unvaccinated)	Narrative description	Laboratory-confirmed infection	Yes, ROBINS-I; GRADE	Yes
**Hughes (2020), Canada** [80]	No	Surveillance methods (vaccine registry), n=36	General population	Other: measles-containing vaccine (not reported)	Narrative description	Symptomatic disease	Yes, ROBINS-I	Yes
**Quach (2020), USA** [81]	Yes	Cohort design; Case-control design, n=14	Other (Pregnant and/or lactating women)	Seasonal influenza; Pandemic influenza (unvaccinated)	(1-RR)*100; (1-OR)*100	Laboratory-confirmed infection; Symptomatic disease	Yes, NOS	No

Author (year of publication), Country	Included RCTs (yes/no)	Observational study designs studied, (n)	Population of Interest	Vaccine(s) assessed (Comparator)	Definitions of VE, effect estimate (if applicable)	VE-specific outcomes	Used a RoB tool (yes/no). If yes, name of RoB tool	Modified RoB tool (yes/no/unclear/ not applicable) If yes, see Table S3.
**Jarvis (2020), UK** [82]	Yes	Cohort design; Case-control design; Screening method, n=15	Other (Pregnant and/or lactating women); Pediatric population (infants)	Seasonal influenza; Pandemic influenza (not reported)	Narrative description	Laboratory-confirmed infection; Symptomatic disease; Contact with HCP	Yes, NHLBI tool	No
**Vygen-Bonnet (2020), Germany** [83]	Yes	Cohort design; Case-control design, n=8	Other (Pregnant and/or lactating women); Pediatric population (infants)	Pertussis (unvaccinated)	(1-RR)*100	Laboratory-confirmed infection; Death; Hospitalization	Yes, ROBINS-I; GRADE	No
**Kalligeros (2020), USA** [84]	No	Test-negative design, n=28	Pediatric population	Seasonal influenza (not reported)	Pooled VE, effect estimate not specified	Hospitalization	Yes, NOS	No
**Berman-Rosa (2020), Canada** [85]	Yes	Cohort design; Case-control design; Indirect cohort, n=13	Pediatric population	Pneumococcal disease (unvaccinated)	(1-OR)*100; (1-IRR)*100	Laboratory-confirmed infection; Symptomatic disease	Yes, Other (NACI guidelines for quality assessment)	Yes
**Di Pietrantonj (2020), Italy** [86]	Yes	Cohort design; Case-control design; Case-only ecological method studies, n=51	Pediatric population	Varicella; Measles/mumps/rubella (MMR) (unvaccinated)	(1-RR)*100	Laboratory-confirmed infection; Symptomatic disease	Yes, NOS; GRADE	No
**Kandeil (2020), Belgium** [87]	No	Cohort design; Case-control design, n=11	Other (Pregnant and/or lactating women); Pediatric population (infants)	Pertussis (not reported)	(1-OR)*100; (1-RR)*100; (1-HR)*100	Laboratory-confirmed infection; Symptomatic disease; Death; Hospitalization	No (narrative description/self-developed methods)	N/A
**Yitbarek (2020), Ethiopia** [88]	Yes	Cohort design; Cross-sectional design; Case-control design; Multinational surveillance data analysis, n=6	General population; Pediatric population	BCG (unvaccinated)	Narrative description	Symptomatic disease; Death; Hospitalization	Yes, JBI	No
**Mo (2020), China** [89]	Yes	Cohort design (retrospective), n=5	Other (People with underlying chronic condition)	Pneumococcal disease (unvaccinated; placebo)	(1-RR)*100, where RR = OR / [(1 - P0) + (P0 * OR)], where P0 is the event incidence in the control group	Laboratory-confirmed infection; Symptomatic disease; Death; Hospitalization	Yes, NOS; GRADE	No
**Zangiabadian (2020), Iran** [90]	Yes	Cohort design; Case-control design, n=11	General population	Seasonal influenza (unvaccinated)	Narrative description	Death	Yes, JBI	No
**McMillan (2021), Australia** [91]	Yes	Cohort design; Cross-sectional design; Case-control design; Interrupted time series, n=22	General population; Pediatric population	Meningococcal disease (unvaccinated; pre- and post-vaccination period)	(1-OR)*100; (1-RR)*100	Laboratory-confirmed infection; Symptomatic disease	Yes, ROBINS-I	No

Author (year of publication), Country	Included RCTs (yes/no)	Observational study designs studied, (n)	Population of Interest	Vaccine(s) assessed (Comparator)	Definitions of VE, effect estimate (if applicable)	VE-specific outcomes	Used a RoB tool (yes/no). If yes, name of RoB tool	Modified RoB tool (yes/no/unclear/ not applicable) If yes, see Table S3.
**Lee (2021), Canada** [92]	Yes	Cohort design; Test-negative design, n=12	Elderly population	Seasonal influenza (seasonal influenza)	(1-OR)*100; Other (pooled relative VE (rVE))	Laboratory-confirmed infection; Death; Hospitalization; Contact with HCP	Yes, Downs and Black checklist	Yes
**van den Boogaard (2021), Netherlands** [93]	Yes	Not reported, n=1	Pediatric population; Other (Pregnant and/or lactating women)	Other (rubella-containing vaccines) (not reported)	(1-RR)*100	Not reported	Yes, GRADE (as RoB assessment tool)	No
**Brown (2021), USA** [94]	Yes	Cohort design; Cross-sectional design, n=33	General population	HPV (unvaccinated; other type of vaccine)	Narrative description	Symptomatic disease (Type-specific anogenital HPV prevalence/incidence in vaccinated versus unvaccinated populations or comparing various dosing schedules, genital warts)	Yes, ROBINS-I	Unclear
**Boddington (2021), UK** [95]	No	Case-control design; Test-negative design, n=37	Pediatric population	Seasonal influenza (not reported)	Pooled VE, effect estimate not specified	Hospitalization; Laboratory-confirmed infection	Yes, ROBINS-I	Unclear
**Xu (2021), Canada** [96]	Yes	Cohort design; Cross-sectional design; Case-control design, n=54	Pediatric population	Measles (MCV1, MCV2) (unvaccinated)	(1-OR)*100; (1-RR)*100	Laboratory-confirmed infection; Symptomatic disease	Yes, ROBINS-I; GRADE	Yes
**Wall (2021), USA** [97]	Yes	Cohort design; Test-negative design, n=10	Pediatric population	Seasonal influenza (unvaccinated)	Narrative description	Laboratory-confirmed infection; Symptomatic disease; Hospitalization	Yes, NHLBI tool	Unclear
**Coleman (2021), Canada** [98]	Yes	Cohort design; Case-control design; Test-negative design, n=20	Elderly population	Seasonal influenza (unvaccinated; non-influenza comparator vaccine)	(1-OR)*100; (1-RR)*100; (1-IRR)*100	Laboratory-confirmed infection; Symptomatic disease (clinically diagnosed influenza)	Yes, ROBINS-I; GRADE	No
**Zhang (2021), China** [99]	No	Cohort design, n=12	Pediatric population	Varicella (not reported)	(1-RR)*100	Symptomatic disease	Yes, NOS; GRADE	Yes
**Tsentemeidou (2021), Greece** [100]	Yes	Cross-sectional design, n=4	General population; Pediatric population; Other (Young men who have sex with men)	HPV (unvaccinated)	(1-RR)*100	Laboratory-confirmed infection	Yes, NIH tool	No
**Harder (2021), Germany** [101]	Yes	Cohort design; Case-control design; Test-negative design, n=28	General population; Other (Healthcare workers)	COVID-19 (unvaccinated)	Narrative description	Laboratory-confirmed infection (asymptomatic SARS-CoV2 infections); Symptomatic disease	Yes, ROBINS-I	Yes

**Author (year of publication), Country**	**Included RCTs (yes/no)**	**Observational study designs studied, (n)**	**Population of Interest**	**Vaccine(s) assessed (Comparator)**	**Definitions of VE, effect estimate (if applicable)**	**VE-specific outcomes**	**Used a RoB tool (yes/no). If yes, name of RoB tool**	**Modified RoB tool (yes/no/unclear/ not applicable) If yes, see Table S3.**
**Nielsen (2021), Denmark** [102]	Yes	Cohort design; Cross-sectional design; Case-control design, n=8	General population	HPV (unvaccinated)	Narrative description	Laboratory-confirmed infection	Yes, NIH tool	No
**Harder (2021), Germany** [103]	No	Cohort design; Cross-sectional design; Test-negative design, n=17	General population; Elderly population (Residents of long term care); Other (Healthcare workers)	COVID-19 (unvaccinated)	Pooled VE, effect estimate not specified	Laboratory-confirmed infection; Symptomatic disease (severe disease); Hospitalization; Disease severity	Yes, ROBINS-I	No
**Sharif (2021), Bangladesh** [104]	Yes	Observational study, n=1	General population	COVID-19 (placebo)	Narrative description	Laboratory-confirmed infection	Yes, Other (SYRCLE)	No
**Liu (2021), China** [105]	Yes	Cohort design; Cross-sectional design; Case-control design, n=32	General population; Elderly population; Other (People with underlying chronic condition); Other (Healthcare workers)	COVID-19 (unvaccinated)	Pooled VE, effect estimate not specified	Laboratory-confirmed infection; Symptomatic disease; Mortality; Hospitalization (ICU admissions)	Yes, NOS; AHRQ checklist	Yes
**Chen (2021), China** [106]	Yes	Cohort design, n=14	Pediatric population	Varicella (unvaccinated)	(1-RR)*100	Symptomatic disease (varicella infection)	Yes, NOS	Yes
**Kow (2021), UK** [107]	No	Cohort design; Case-control design, n=19	General population; Elderly population; Other (Healthcare workers)	COVID-19 (unvaccinated)	(1-HR); (1-IRR); (1-OR)	Laboratory-confirmed infection	Yes, NOS	No
**Andani (2022)** [108]	Yes	Not reported, n=28	Pediatric population	Hepatitis A (unvaccinated)	Narrative description	Symptomatic disease (reported Hepatitis A cases)	No (narrative description/self-developed methods)	No
**Almasri (2022), Belgium** [109]	Yes	Cohort design; Case-control design; "Descriptive non-experimental studies or retrospective comparisons of vaccine outcomes"; Survey study (supplementary material), n=32	Other (People with underlying chronic condition)	All vaccines recommended to adults with DM over age 65 (not reported)	Narrative description	Mortality; Hospitalization	Yes, Other (PRISMA-guided checklist)	Unclear
**Sim (2021), USA** [110]	Yes	Cohort design; Cross-sectional design, n=4	Other (People with underlying chronic condition)	Seasonal influenza (unvaccinated)	Narrative description	Laboratory-confirmed infection; Symptomatic disease; Mortality; Hospitalization	Yes, ROBINS-I	No
**Fu (2022), Singapore** [111]	No	Cohort design; Cross-sectional design; Case-control design, n=1	Pediatric population; Other (Pregnant and/or lactating women)	COVID-19 (unvaccinated)	Narrative description	Symptomatic disease	Yes, NIH tool	No
**Wang (2021), Canada** [112]	No	Cohort design; Cross-sectional design; Case-control design, n=96	General population; Pediatric population; People with underlying chronic condition	HPV (unvaccinated)	Narrative description	Laboratory-confirmed infection; Symptomatic disease	Yes, ROBINS-I	Yes

Author (year of publication), Country	Included RCTs (yes/no)	Observational study designs studied, (n)	Population of Interest	Vaccine(s) assessed (Comparator)	Definitions of VE, effect estimate (if applicable)	VE-specific outcomes	Used a RoB tool (yes/no). If yes, name of RoB tool	Modified RoB tool (yes/no/unclear/ not applicable) If yes, see Table S3.
**Sung (2022), Sweden** [113]	No	Retrospective cohort design, n=3	Other (People with underlying chronic condition)	COVID-19 (unvaccinated)	(1-OR)*100	Laboratory-confirmed infection; Mortality	Yes, NOS	Unclear
**Kow (2022), UK** [114]	No	Cohort design; Case-control design; Test-negative design, n=7	General population	COVID-19 (unvaccinated)	Other [(pooled HR - 1) / HR or (pooled OR - 1) / OR)]	Laboratory-confirmed infection	Yes, NOS	No
**Izurieta (2022), Belgium** [115]	Yes	Cohort design; Case-control design; Surveillance active prospective, ecological, n=4	Pediatric population	Pneumococcal disease (unvaccinated)	Narrative description	Laboratory-confirmed infection; Symptomatic disease	Yes, ROBINS-I	No
**Puig-Barberà (2022), Spain** [116]	No	Cohort design; Test-negative design, n=12	General population; Elderly population	Seasonal influenza (seasonal influenza)	(1-RR)*100	Laboratory-confirmed infection; Hospitalization; Healthcare utilisation ('outpatient consultation', emergency room visit)	Yes, ROBINS-I	No
**Kow (2022), Malaysia** [117]	No	Cohort design; Case-control design; Test-negative design, n=7	General population	COVID-19 (unvaccinated)	Other [(pooled HR - 1) / HR or (pooled OR - 1) / OR)]	Laboratory-confirmed infection	Yes, NOS	Unclear
**Huang (2022), Taiwan** [118]	Yes	Cohort design, n=1	Other (COVID-19 patients)	COVID-19 (unvaccinated)	(1-RR)*100	Symptomatic disease	Yes, NOS	No
**Bhurwal (2022), USA** [119]	No	Cohort design; Survey-based studies, n=21	Other (People with underlying chronic condition)	COVID-19 (unvaccinated)	(1-OR)*100	Symptomatic disease	Yes, ROBINS-I; NIH tool	Unclear
**Gertosio (2022), Italy** [120]	Yes	Cohort design; Case-control design, n=3	Pediatric population (children with chronic conditions requiring therapy with biologics)	Seasonal influenza; Measles/mumps/rubella (MMR) (not reported)	Narrative description	Disease exacerbation/deterioratio n; Relapse of disease	Yes, Other (STROBE)	No
**Zheng (2022), China** [121]	No	Cohort design; Case-control design; Test-negative case-control, n=51	General population; Elderly population; Other (Healthcare workers); Other (hospitalized adults, prioritized population, veterans, adolescents)	COVID-19 (not reported)	(1-OR)*100; (1-RR)*100; (1-HR)*100; (1-IRR)*100	Laboratory-confirmed infection; Mortality; Hospitalization;	Yes, NOS	No
**Galmiche (2022), France** [122]	Yes	Cohort design; Test-negative design, n=4	Other (People with underlying chronic condition)	COVID-19 (not reported)	Narrative description	Laboratory-confirmed infection; Hospitalization	Yes, NIH tool	No
**Marra (2022), USA** [123]	No	Cohort design, n=33	Other (People with underlying chronic condition)	COVID-19 (unvaccinated; vaccinated control group)	(1-DOR)*100	Symptomatic disease	Yes, Downs and Black checklist	Yes

Author (year of publication), Country	Included RCTs (yes/no)	Observational study designs studied, (n)	Population of Interest	Vaccine(s) assessed (Comparator)	Definitions of VE, effect estimate (if applicable)	VE-specific outcomes	Used a RoB tool (yes/no). If yes, name of RoB tool	Modified RoB tool (yes/no/unclear/ not applicable) If yes, see Table S3.
**Gärtner (2022), Germany** [124]	Yes	Retrospective cohort design, n=7	Elderly population	Seasonal influenza (seasonal influenza)	Other (pooled relative VE (rVE))	Laboratory-confirmed infection; Symptomatic disease; Death; Hospitalization; Contact with HCP	Yes, ROBINS-I	No
**Ssentongo (2022), USA** [125]	Yes	Cohort design; Case-control design, n=13	General population	COVID-19 (unvaccinated)	(1-IRR)*100	Laboratory-confirmed infection; Symptomatic disease	Yes, NOS; GRADE	No
**Prasad (2022), UK** [126]	Yes	Cohort design; Case-control design; Test-negative design, n=3	Other (Pregnant and/or lactating women)	COVID-19 (unvaccinated)	(1-HR)*100	Laboratory-confirmed infection; Death; Hospitalization; Symptomatic disease; Pregnancy-related outcomes (delivery and fetal)	Yes, ROBINS-I	No
**Gupta (2022), Canada** [127]	No	Cohort design, n=7	Other (People with underlying chronic condition)	Seasonal influenza (unvaccinated)	(1-RR)*100	Death; Hospitalization	Yes, NOS; GRADE	Yes
**Zeng (2022), China** [128]	Yes	Cohort design; Case-control design; Test-negative design, n=46	General population; Pediatric population; Elderly population (residents of long-term care facilities); Other (Healthcare workers)	COVID-19 (unvaccinated)	Pooled VE, effect estimate not specified	Laboratory-confirmed infection; Symptomatic disease	Yes, NOS	No
**Tsiakos (2022), Greece** [129]	No	Retrospective cohort design, n=5	Other (people with cancer)	Seasonal influenza (unvaccinated)	(1-OR)*100	Mortality; Incidence of disease	Yes, JBI	No
**Piechotta (2022), Germany** [130]	No	Cohort design, n=22	Other (people with cancer)	COVID-19 (not reported)	Narrative description	Laboratory-confirmed infection; Symptomatic disease; Mortality; Hospitalization	Yes, Other (RoB-OPS); GRADE	No
**Marra (2022), Brazil** [131]	No	Cohort design; Case-control design; Before-after (Pre-post), time series, n=48	Pediatric population	Pneumococcal disease (unvaccinated; period prior to pneumococcal vaccination)	(1-OR)*100; (1-RR)*100	Symptomatic disease	Yes, NHLBI tool	Unclear
**Baradaran (2022), UK** [132]	No	Cohort design, n=8	General population; Healthcare workers	COVID-19 (unvaccinated)	Pooled VE, effect estimate not specified	Laboratory-confirmed infection; Symptomatic disease; Mortality; Hospitalization	Yes, JBI	No

Author (year of publication), Country	Included RCTs (yes/no)	Observational study designs studied, (n)	Population of Interest	Vaccine(s) assessed (Comparator)	Definitions of VE, effect estimate (if applicable)	VE-specific outcomes	Used a RoB tool (yes/no). If yes, name of RoB tool	Modified RoB tool (yes/no/unclear/ not applicable) If yes, see Table S3.
**Domnich (2022), Italy** [133]	No	Retrospective cohort design, n=10	Elderly population	Seasonal influenza (seasonal influenza)	(1 - RR)*100; (1 - HR)*100; (1 - OR)*100; Other (pooled relative VE (rVE))	Hospitalization; Healthcare utilisation (medical encounters, any cardio-respiratory condition, pneumonia, asthma/COPD/bronchial, coronary artery, myocardial infarction, congestive heart failure, cerebrovascular events, stroke, influenza-related office visits)	Yes, ROBINS-I; Other (GRACE checklist)	No
**Zou (2022), USA** [134]	No	Cohort design; Case-control design; Observational study, n=13	Unclear	COVID-19 (unvaccinated)	(1-OR)*100	Laboratory-confirmed infection	Yes, NOS	No
**Kechagias (2022), UK** [135]	Yes	Cohort design; Case-control design, n=12	General population	HPV (unvaccinated)	(1-RR)*100	Symptomatic disease (HPV-related lesion recurrence (e.g., CIN2+, CIN3))	Yes, ROBINS-I; GRADE	No
**Shao (2022), China** [136]	No	Cohort design; Case-control design; Test-negative design, n=113	General population; Elderly population; Healthcare workers; Other: adolescents, close contacts of COVID-19 casw	COVID-19 (unvaccinated)	(1-OR)*100; (1-RR)*100; (1-HR)*100; (1-IRR)*100	Laboratory-confirmed infection; Symptomatic disease; Mortality; Hospitalization; Healthcare utilisation (emergency department or urgent care visits)	Yes, NOS	No
**Külper-Schiek (2022), Germany** [137]	No	Cohort design; Case-control design; Test-negative design, n=26	General population; People with underlying chronic condition; Healthcare workers; Other: Veterans	COVID-19 (unvaccinated)	Narrative description	Laboratory-confirmed infection; Symptomatic disease; Mortality; Hospitalization	Yes, ROBINS-I; GRADE	No
**Xia (2022), China** [138]	Yes	Cohort design, n=19	General population; People with underlying chronic condition	Herpes zoster (Shingles) (unvaccinated)	(1-IRR)*100	Laboratory-confirmed infection; Symptomatic disease	Yes, NOS	No
**Petras (2022), Czech Republic** [139]	No	Cohort design; Cross-sectional design; Case-control design, n=290	General population	COVID-19 (unvaccinated)	Pooled VE, effect estimate not specified	Laboratory-confirmed infection; Symptomatic disease	Yes, NOS; GRADE	No
**Markowitz (2022), Canada** [140]	No	Cohort design; Cross-sectional design; Case-control design, n=35	General population	HPV (unvaccinated)	Narrative description	Laboratory-confirmed infection; Symptomatic disease (anogenital warts, cervical abnormalities)	Yes, ROBINS-I	Yes

Author (year of publication), Country	Included RCTs (yes/no)	Observational study designs studied, (n)	Population of Interest	Vaccine(s) assessed (Comparator)	Definitions of VE, effect estimate (if applicable)	VE-specific outcomes	Used a RoB tool (yes/no). If yes, name of RoB tool	Modified RoB tool (yes/no/unclear/ not applicable) If yes, see Table S3.
**Mbinta (2022), UK** [141]	No	Cohort design; Case-control design, n=22	Elderly population	Herpes zoster (Shingles) (unvaccinated)	(1-HR)*100	Symptomatic disease; Hospitalization; Quality of life	Yes, JBI; GRADE	No
**Au (2022), China** [142]	Yes	Cohort design; Case-control design, n=47	General population	COVID-19 (unvaccinated)	(1-OR)*100	Symptomatic disease; Mortality; Hospitalization	Yes, ROBINS-I	Unclear
**Deng (2022), China** [143]	Yes	Cohort design; Case-control design, n=11	General population	COVID-19 (COVID-19)	(1-OR)*100	Laboratory-confirmed infection; Symptomatic disease; Mortality; Hospitalization	Yes, NOS	No
**Gao (2022), China** [144]	No	Cohort design; Cross-sectional design; Case-control design, n=18	General population	COVID-19 (unvaccinated)	(1-RR)*100	Symptomatic disease (long COVID)	Yes, NOS; AHRQ checklist	No
**Rahmani (2022), Netherlands** [145]	No	Cohort design; Case-control design, n=54	General population; Elderly population (Nursing home residents); Other (Healthcare workers)	COVID-19 (unvaccinated)	(1-OR)*100; (1-RR)*100; (1-HR)*100	Laboratory-confirmed infection; Mortality; Hospitalization	Yes, NOS	No
**Martinez (2022), USA** [146]	No	Cohort design, n=26	General population (infant vaccination on outcomes in young children, adolescents, and adults)	Other: Tuberculosis Bacillus Calmette–Guérin (BCG) (unvaccinated)	(1-OR)*100	Laboratory-confirmed infection	Yes, NOS	No
**Berild (2020), Norway** [147]	Yes	Cohort design; Case-control design; Test-negative design; Indirect cohort, n=10	Elderly population	Pneumococcal disease (unvaccinated; placebo)	Narrative description	Laboratory-confirmed infection; Symptomatic disease; Mortality (all-cause, pneumococcal and/or serotype-specific disease)	Yes, NOS	Yes
**Bechini (2020), Italy** [148]	No	Cohort design; Case-control design, n=6	Elderly population; Other (People with underlying chronic condition)	Seasonal influenza (unvaccinated)	(1-RR)*100	Mortality; Hospitalization	Yes, NOS; GRADE	No
**Murunga (2020), Kenya** [149]	No	Case-control design, n=13	Pediatric population	Rotavirus (unvaccinated)	(1-OR)*100	Hospitalization	Yes, NOS; GRADE	No
**Yang (2021), China** [150]	No	Case-control design; Test-negative design, n=21	General population	Seasonal influenza (unvaccinated)	(1-OR)*100	Laboratory-confirmed infection	Yes, NOS	No
**Okoli (2021), UK** [151]	No	Test-negative design, n=72	General population; Elderly population	Seasonal influenza (not reported)	Pooled VE, effect estimate not specified	Laboratory-confirmed infection	No (narrative description/self-developed methods)	N/A
**Iheanacho (2021), Nigeria** [152]	Yes	Not reported, n=11	General population	COVID-19 (unvaccinated)	Narrative description	Laboratory-confirmed infection; Symptomatic disease; Mortality	Yes, NHLBI tool	Unclear

**Author (year of publication), Country**	**Included RCTs (yes/no)**	**Observational study designs studied, (n)**	**Population of Interest**	**Vaccine(s) assessed (Comparator)**	**Definitions of VE, effect estimate (if applicable)**	**VE-specific outcomes**	**Used a RoB tool (yes/no). If yes, name of RoB tool**	**Modified RoB tool (yes/no/unclear/ not applicable) If yes, see Table S3.**
**Fan (2021), China** [153]	Yes	Observational study, n=4	General population	COVID-19 (unvaccinated)	(1-RR)*100	Symptomatic disease; Laboratory-confirmed infection (Asymptomatic COVID-19 cases)	Yes, Other (QUIPS tool)	No
**Perego (2021), Italy** [154]	Yes	Cohort design; Cross-sectional design, n=2	Other (US military service members on active duty)	Seasonal influenza (unvaccinated)	(1-OR)*100	Symptomatic disease; Hospitalization	Yes, NOS; ROBINS-I	No
**Li (2021), China** [155]	Yes	Cohort design; Case-control design, n=10	Healthcare workers	Seasonal influenza (unvaccinated)	(1-RR)*100	Laboratory-confirmed infection; Symptomatic disease; Absenteeism (incidence of absenteeism, the number of working days lost per person)	Yes, NOS	Unclear
**Hayawi (2021), United Arab Emirates** [156]	Yes	Cohort design; Case-control design; Test-negative design, n=29	General population	COVID-19 (not reported)	Narrative description	Laboratory-confirmed infection	Yes, Other (The risk of bias in the prevalence tool)	No
**Cheng (2021), Taiwan** [157]	No	Cohort design; Case-control design, n=39	General population; Elderly population (residents of long-term care facility); Other (People with underlying chronic condition); Other (Healthcare workers);	COVID-19 (unvaccinated; number of doses received)	(1-OR)*100; (1-HR)*100; (1 - IRR)*100	Laboratory-confirmed infection; Symptomatic disease; Mortality; Hospitalization;	Yes, ROBINS-I	Unclear
**Pormohammad (2021), Canada** [158]	Yes	Cohort design; Case-control design, n=35	General population	COVID-19 (unvaccinated)	Pooled VE, effect estimate not specified	Laboratory-confirmed infection; Mortality; Hospitalization; Symptomatic disease	Yes, JBI	No
**Marra (2021), USA** [159]	No	Cohort design; Case-control design, n=16	Other (Healthcare workers)	COVID-19 (unvaccinated)	(1-DOR)*100	Laboratory-confirmed infection	Yes, Downs and Black checklist	Yes
**Meggiolaro (2022), Italy** [160]	No	Cohort design; Cross-sectional design; Case-control design; Test-negative design, n=31	General population; Elderly population (long-term care facility residents); Other (Healthcare workers)	COVID-19 (unvaccinated; pre- and post-vaccination period)	(1-RR)*100	Laboratory-confirmed infection; Symptomatic disease	Yes, NOS	Yes
**Ma (2022), China** [161]	No	Cohort design, n=6	Other (Pregnant and/or lactating women)	COVID-19 (unvaccinated)	(1-OR)*100	Laboratory-confirmed infection; Symptomatic disease; Mortality; Hospitalization	Yes, NOS	No
**Mahumud (2022), Australia** [162]	No	Cohort design; Case-control design; Case-case design, n=11	Unclear	COVID-19 (unvaccinated)	(1-RR)*100	Laboratory-confirmed infection	Yes, JBI	No

**Author (year of publication), Country**	**Included RCTs (yes/no)**	**Observational study designs studied, (n)**	**Population of Interest**	**Vaccine(s) assessed (Comparator)**	**Definitions of VE, effect estimate (if applicable)**	**VE-specific outcomes**	**Used a RoB tool (yes/no). If yes, name of RoB tool**	**Modified RoB tool (yes/no/unclear/ not applicable) If yes, see Table S3.**
**Ghazy (2022), Egypt** [163]	Yes	Cohort design; Case-control design; Observational study, n=14	General population; Elderly population (residents in a long-term care facility, nursing home residents, Veterans, and others); Other (Healthcare workers)	COVID-19 (unvaccinated)	(1-OR)*100	Laboratory-confirmed infection; Symptomatic disease; Mortality	Yes, NHLBI tool	No
**Chang (2022), China** [164]	No	Test-negative design, n=19	General population; Pediatric population (Adolescent patients); Other (Healthcare workers)	COVID-19 (unvaccinated)	(1-OR)*100	Laboratory-confirmed infection	Yes, NOS	No
**Wang (2022), China** [165]	Yes	Cohort design; Case-control design; Test-negative design, n=38	General population; Pediatric population; Elderly population; Other (Healthcare workers)	COVID-19 (unvaccinated; asymptomatic vs symptomatic cases)	(1-OR)*100	Laboratory-confirmed infection	Yes, NOS	No
**Cai (2022), China** [166]	Yes	Cohort design; Case-control design; Case series; Non-randomized, n=28	Other (women with precancerous lesions)	HPV (unvaccinated; not reported)	(1-IRR)*100	Laboratory-confirmed infection (HSIL regression, HPV clearance; would fall under lab-confirmed infection), Symptomatic disease (HSIL recurrence)	Yes, NOS; ROBINS-I; Other (Recommendations by NICE for case-series)	Unclear
**Jansen (2022), Netherlands** [167]	Yes	Cohort design, n=8	Pediatric population	Pneumococcal disease; Meningococcal disease; HPV; Varicella; Measles/mumps/rubella (MMR); Hepatitis A and B (unvaccinated)	Narrative description	Symptomatic disease; Laboratory-confirmed infection	Yes, Other (OCEBM tool)	Unclear
**Zhu (2022), China** [168]	No	Cohort design; Test-negative case-control, n=7	Unclear	COVID-19 (number of doses received)	(1-OR)*100	Laboratory-confirmed infection	Yes, NOS	Yes
**Foucambert (2022), USA** [169]	Yes	Case-control design, n=1	Pediatric population	Dengue (unvaccinated)	Narrative description	Symptomatic disease	Yes, JBI	Yes
**Marra (2022), Brazil** [170]	Yes	Cohort design; Case-control design, n=16	Unclear	COVID-19 (unvaccinated)	(1-DOR)*100	Laboratory-confirmed infection; Hospitalization	Yes, Downs and Black checklist	Yes
**Liu (2022), China** [171]	Yes	Cohort design; Case-control design, n=6	Other (People with underlying chronic condition); Other (USA military)	Seasonal influenza (unvaccinated)	(1-OR)*100	Symptomatic disease (atrial fibrillation, ventricular arrythmia, flutter, primary cardiac arrest)	Yes, NOS	No

Author (year of publication), Country	Included RCTs (yes/no)	Observational study designs studied, (n)	Population of Interest	Vaccine(s) assessed (Comparator)	Definitions of VE, effect estimate (if applicable)	VE-specific outcomes	Used a RoB tool (yes/no). If yes, name of RoB tool	Modified RoB tool (yes/no/unclear/ not applicable) If yes, see Table S3.
**Sabu (2022), Australia** [172]	Yes	Cohort design; Case-control design; Test-negative design, n=13	Pediatric population	COVID-19 (unvaccinated)	(1-OR)*100	Hospitalization (ICU admissions); Symptomatic disease	Yes, Other (QATSDD tool)	Yes
**Wallace (2022), Ethiopia** [173]	No	Cohort design; Test-negative design; Longitudinal household survey, n=26	General population; Other (Pregnant and/or lactating women); Other (Healthcare workers); Other (groups prioritized for vaccination)	COVID-19 (not reported)	(1-OR)*100; (1-RR)*100; (1-HR)*100	Laboratory-confirmed infection; Symptomatic disease; Mortality; Hospitalization	Yes, NOS; GRADE	No
**Angkasekwinai (2022), Thailand** [174]	Yes	Cohort design; Case-control design; Test-negative design, n=7	General population; Healthcare workers	COVID-19 (unvaccinated)	Narrative description	Laboratory-confirmed infection; Symptomatic disease; Mortality; Hospitalization	Yes, NOS	Unclear
**Ruiz (2023), USA** [175]	No	Cohort design; Cross-sectional design; Case reports, n=3	Other (people with cancer)	COVID-19 (unvaccinated; non-ICI interventions)	(1-RR)*100	Laboratory-confirmed infection	Yes, NOS	No
**Wu (2023), China** [176]	No	Cohort design; Test-negative design; Test-negative case-control, n=4	General population	COVID-19 (unvaccinated)	(1-OR)*100	Laboratory-confirmed infection; Symptomatic disease	Yes, ROBINS-I	No
**Paul (2023), Qatar** [177]	No	Cohort design; Case-control design; Test-negative design, n=51	General population	COVID-19 (unvaccinated)	Narrative description	Laboratory-confirmed infection; Mortality; Hospitalization; Disease severity	Yes, NOS	Unclear
**Jones-Gray (2023), Australia** [178]	No	Cohort design; Case-control design; Test-negative design, n=83	General population	Seasonal influenza (unclear)	Pooled VE, effect estimate not specified	Laboratory-confirmed infection	Yes, ROBINS-I; GRADE	No
**Hameed (2023), Pakistan** [179]	No	Cohort design, n=6	Pregnant and/or lactating women	COVID-19 (unvaccinated)	(1-OR)*100	Laboratory-confirmed infection; Hospitalization (ICU admissions)<br><br>Pregnancy-related outcomes (delivery and fetal)	Yes, NOS	No
**Xu (2023), China** [180]	Yes	Cohort design; Case-control design; Descriptive studies, n=21	General population	COVID-19 (unvaccinated)	(1-OR)*100; (1-RR)*100; (1-HR)*100	Laboratory-confirmed infection; Symptomatic disease	Yes, NOS; AHRQ checklist	No

Author (year of publication), Country	Included RCTs (yes/no)	Observational study designs studied, (n)	Population of Interest	Vaccine(s) assessed (Comparator)	Definitions of VE, effect estimate (if applicable)	VE-specific outcomes	Used a RoB tool (yes/no). If yes, name of RoB tool	Modified RoB tool (yes/no/unclear/ not applicable) If yes, see Table S3.
**Tan (2023), Singapore** [181]	Yes	Cross-sectional design; Cohort design; Non-randomized controlled trial, n=6	Other (People with underlying chronic condition)	COVID-19 (not reported)	Narrative description	Symptomatic disease	Yes, ROBINS-I	No
**Menegale (2023), Italy** [182]	No	Cohort design; Case-control design; Test-negative design, n=40	General population; Pediatric population; Elderly population	COVID-19 (unvaccinated; individuals who received the vaccine not earlier than 14 days)	Other (VE(t) = A * e^(-w * t), VE(t): Represents vaccine effectiveness as a function of time A: A constant representing the initial vaccine effectiveness. e: The base of the natural logarithm. w: A rate constant representing the rate at which vaccine effectiveness wanes over time. t: Time.)	Laboratory-confirmed infection; Symptomatic disease	Yes, NOS	No
**Comber (2021), Ireland** [183]	Yes	Cohort design; Test-negative design, n=9	General population; Elderly population	Seasonal influenza (unvaccinated; placebo; other vaccines of influenza)	(1-OR)*100; (1-RR)*100; (1-HR)*100; (1-IRR)*100	Laboratory-confirmed infection; Mortality; Hospitalization	Yes, ROBINS-I; GRADE	No
**Jordan (2023), Ireland** [184]	Yes	Cohort design; Test-negative design, n=4	General population	Seasonal influenza (unvaccinated; SD-IIV3)	(1-OR)*100	Laboratory-confirmed infection; Hospitalization; Healthcare utilisation (influenza-related hospital encounters, influenza-related office visits); Symptomatic disease (influenza-like illness)	Yes, ROBINS-I; GRADE	No
**O Murchu (2022), Ireland** [185]	No	Cohort design; Case-control design, n=48	General population; Elderly population	Seasonal influenza (unvaccinated; other type of influenza vaccines)	(1-RR)*100; (1-OR)*100	Laboratory-confirmed infection	Yes, ROBINS-I; GRADE	No
**Wu (2023), Canada** [186]	Yes	Cohort design; Test-negative design, n=65	General population	COVID-19 (unvaccinated)	(1-RR)*100	Laboratory-confirmed infection; Symptomatic disease; Mortality; Hospitalization	Yes, ROBINS-I	Yes
**Marra (2022), USA** [187]	No	Cohort design; Case-control design, n=10	General population	COVID-19 (unvaccinated)	(1-DOR)*100	Symptomatic disease (post-COVID-19 conditions defined as a	Yes, Downs and Black checklist	Yes

Author (year of publication), Country	Included RCTs (yes/no)	Observational study designs studied, (n)	Population of Interest	Vaccine(s) assessed (Comparator)	Definitions of VE, effect estimate (if applicable)	VE-specific outcomes	Used a RoB tool (yes/no). If yes, name of RoB tool	Modified RoB tool (yes/no/unclear/ not applicable) If yes, see Table S3.
						wide range of health symptoms that are present 3 or more weeks after having COVID-19)		
**Lopez-Olivo (2022), USA** [188]	Yes	Cohort design; Case-control design, n=13	Other (people with cancer)	Seasonal influenza (unvaccinated)	Narrative description	Laboratory-confirmed infection; Symptomatic disease (cancer-related outcomes (survival, progression-free survival))	Yes, NOS	No
**van den Berg (2022), Netherlands** [189]	No	Cohort design; Case-control design; Test-negative design; Retrospective record review, n=17	Other (People with underlying chronic condition)	COVID-19 (unvaccinated)	(1-RR)*100; (1-OR)*100	Symptomatic disease (severe disease, breakthrough-infection, pneumonia); Mortality; Hospitalization	Yes, ROBINS-I	No
**Sikjær (2023), Denmark** [190]	Yes	Cohort design; Case-control design, n=8	Elderly population; Other (People with underlying chronic condition)	Pneumococcal disease (unvaccinated)	Narrative description	Laboratory-confirmed infection	Yes, NOS	No
**Mohammed (2023), Australia** [191]	No	Cohort design; Case-control design, n=28	General population; Pediatric population; Elderly population (long-term care residents); Other (People with underlying chronic condition); Other (Healthcare workers)	COVID-19 (unvaccinated)	(1-OR)*100; (1-RR)*100; (1-HR)*100; (1-IRR)*100	Laboratory-confirmed infection; Symptomatic disease; Mortality; Hospitalization; Disease severity	Yes, JBI	Yes
**Beran (2023), USA** [192]	No	Cohort design, n=4	Other (People with underlying chronic condition)	COVID-19 (unvaccinated)	(1-RR)*100	Mortality; Hospitalization; Composite outcomes (Need for invasive mechanical ventilation)	Yes, NOS	Unclear
**Pratama (2022), Indonesia** [193]	No	Cohort design; Case-control design, n=20	General population	COVID-19 (unvaccinated)	(1-OR)*100; (1-RR)*100; (1-HR)*100	Laboratory-confirmed infection; Symptomatic disease; Hospitalization; Disease severity	Yes, NOS	No
**Gao (2022), China** [194]	Yes	Cohort design; Cross-sectional design, n=6	Pediatric population	COVID-19 (unvaccinated)	(1-RR)*100	Laboratory-confirmed infection	Yes, NOS	Yes
**Byambasuren (2022), Australia** [195]	No	Cohort design, n=16	General population; Other (Healthcare workers)	COVID-19 (unvaccinated; number of doses received)	Narrative description	Symptomatic disease (Long COVID-19 illness)	Yes, ROBINS-I	Unclear
**Macilwraith (2023), Australia** [196]	Yes	Cohort design; Cross-sectional design, n=3	General population (Men only)	HPV (unvaccinated)	Narrative description	Laboratory-confirmed infection; Symptomatic disease (oropharyngeal cancer)	Yes, ROBINS-I; NIH tool	No

Author (year of publication), Country	Included RCTs (yes/no)	Observational study designs studied, (n)	Population of Interest	Vaccine(s) assessed (Comparator)	Definitions of VE, effect estimate (if applicable)	VE-specific outcomes	Used a RoB tool (yes/no). If yes, name of RoB tool	Modified RoB tool (yes/no/unclear/ not applicable) If yes, see Table S3.
**Dicembrini (2023), Italy** [197]	No	Cohort design; Case-control design, n=13	Other (People with underlying chronic condition)	Seasonal influenza; Pandemic influenza (unvaccinated)	(1-OR)*100	Mortality; Hospitalization	Yes, NOS	No
**Gao (2023), China** [198]	Yes	Cohort design; Cross-sectional design; Case-control design, n=33	Pediatric population	COVID-19 (unvaccinated)	(1-OR)*100; (1-RR)*100	Laboratory-confirmed infection; Hospitalization	Yes, NOS; AHRQ checklist	No
**Kontovazainitis (2023), Greece** [199]	No	Cohort design, n=5	Pediatric population (infant outcomes); Other (Pregnant and/or lactating women)	COVID-19 (unvaccinated)	(1-OR)*100	Laboratory-confirmed infection; Mortality; Hospitalization	Yes, ROBINS-I	No
**Guo (2023), China** [200]	No	Cohort design; Case-control design; Test-negative design, n=34	General population	COVID-19 (unvaccinated)	(1-OR)*100; (1-RR)*100; (1-HR)*100; (1-IRR)*100	Laboratory-confirmed infection; Symptomatic disease; Mortality; Hospitalization	Yes, ROBINS-I	No
**Piechotta (2023), Germany** [201]	Yes	Cohort design; Cross-sectional design; Case-control design; Test-negative design, n=47	Pediatric population	COVID-19 (unvaccinated)	(1-OR)*100; (1-RR)*100; (1-HR)*100; (1-IRR)*100	Laboratory-confirmed infection; Symptomatic disease (Multisystem inflammatory syndrome in children); Mortality; Hospitalization (ICU admission due to COVID-19),	Yes, ROBINS-I; GRADE	No
**Katoto (2023), South Africa** [202]	Yes	Case-control design, n=9	Pediatric population (adolescents aged 12-17)	COVID-19 (unvaccinated)	Other ((ARU - ARV) / ARU)*100, where ARU is the attack rate in the unvaccinated group and ARV is the attack rate in the vaccinated group	Laboratory-confirmed infection; Symptomatic disease (Multisystem inflammatory syndrome in children); Hospitalization; Asymptomatic infection; Healthcare utilisation (ED visits); Death	Yes, ROBINS-I	No
**Luna (2014), Brazil** [203]	No	Cross-sectional, case-control, cohort, ecologic, n=31	General population	influenza (unvaccinated)	Narrative description	Mortality, hospitalization, symptomatic disease, lab-confirmed infection	No (narrative description/self-developed methods)	N/A

Abbreviations: NOS (Newcastle-Ottawa Scale), GRADE (Grading of Recommendations Assessment, Development and Evaluation), OCEBM (the Oxford Centre for Evidence-Based Medicine), CHERG (Child Health Epidemiology Reference Group), ROBINS-I (Risk of Bias in Non-randomized Studies of Interventions), CASP (Critical Appraisal Skills Programme), NACI (National Advisory Committee on Immunization), EPOC (Effective Practice and Organisation of Care), AHRQ (Agency for Healthcare Research and Quality), NICE (National Institute for Health and Care Excellence), VE (Vaccine Effectiveness), JBI (Joanna Briggs Institute), QATSDD (Quality Assessment Tool for Studies with Diverse Designs), NIH (the National Institutes of Health), QUIPS (the Quality in Prognosis Studies), GSWGTAHC (German Scientific Working Group Technology Assessment for Health Care), ROBANS (Risk Of Bias Assessment Tool for Non-randomized Studies), CoCanCPG (Collaborative Care Clinical Practice Guidelines), EPHPP quality assessment tool (Effective Public Health Practice Project Quality Assessment Tool), SIGN (Scottish Intercollegiate Guidelines Network), SYRCLE (Systematic Review Center for Laboratory animal Experimentation), STROBE (Strengthening the Reporting of Observational Studies in Epidemiology), RoB-OPS (Risk of Bias in Non-randomized Studies of Interventions), NHLBI (National Heart, Lung, and Blood Institute; DOR (Diagnostic Odds Ratio)

Table S2. Risk of Bias Assessment Tools Adapted in Included Studies (N=34)

Adapted RoB tool	N	%
**Newcastle Ottawa Scale (NOS)**	**14**	**41.2%**
**Risk of Bias in Non-randomized Studies - of Interventions (ROBINS-I)**	**9**	**26.5%**
**Downs and Black checklist**	**6**	**17.6%**
Joanna Briggs Institute (JBI)	2	5.9%
Quality Assessment Tool for Studies with Diverse Designs (QATSDD)	1	2.9%
National Advisory Committee on Immunization (NACI) Guidelines	1	2.9%
Cochrane risk of bias tool	1	2.9%

Table S3. Characteristics of Risk-of-bias Tool Adaptations (N=34).*

***Note: The first row for each RoB tool presents the summary of the original tool; subsequent rows show instances of adaptations and corresponding details.**

**RoB tool**	**Summary of the original tool**	
**Newcastle Ottawa Scale (NOS)**	Original NOS employs a 9-star system across three domains (Selection: 4 categories, Comparability: 2 categories, Outcome/Exposure: 3 categories). Items are individually evaluated, with responses earning stars one (in the Selection and Outcome/Exposure domains) and up to two stars (in the Comparability domain) [204].	
**Adapted in (Author, Year)**	**Adapted Aspect**	**Adaptation Details**
**Sun et al. (2021) [2]**	Risk-based scoring system	Low risk: ≤1 inadequate item<br>Medium risk: ≤3 inadequate items<br>High risk: >3 inadequate items<br>Very high risk: No method description
**Ramsay et al. (2019) [3]**	Risk-based scoring system	Low risk: ≤1 missing item<br>Moderate risk: 2-3 missing items<br>High risk: >3 missing items
**Eliakim-Raz et al. (2013) [17]**	Risk-based scoring system	Low risk: ≤1 inadequate item<br>Medium risk: ≤3 inadequate items<br>High risk: >3 inadequate items or no method description
**Meggiolaro et al. (2022) [160]**	Risk-based scoring system	Satisfactory (NOS ≤ 6)<br>Good (NOS > 6)
**Zhu et al. (2022) [168]**	Quality thresholds	Low Quality: 0–3 stars<br>Moderate Quality: 4–6 stars<br>High Quality: 7 stars or more
**Bitterman et al. (2018) [55]**	Assessment criteria	The authors adapted the assessment criteria across its three domains, and by adding evaluations of adjusted analyses and other potential sources of bias, including baseline comparability, sample size calculation, and funding:<br>1) Selection (representativeness of the exposed cohort, selection of the non-exposed cohort, and ascertainment of exposure);<br>2) Comparability of cohorts;<br>3) Outcome (assessment method, length and adequacy of follow-up).
**Chan et al. (2014) [21]**	Assessment criteria	1 or 2 additional star given if either criteria met:<br>if age were compared between groups;

		if comorbidity and/or functional status compared between groups.
**Gao et al. (2022) [194]**	Quality thresholds	Cohort studies were classified as:<br>Low risk of bias: 7-9 stars<br>Moderate risk of bias: 5-6 stars<br>High risk of bias: 0-4 stars
**Zhang et al. (2021) [99]**	Quality thresholds	1–3 points: Low methodological quality<br>4–6 points: Intermediate methodological quality<br>7–9 points: High methodological quality
**Gupta et al. (2022) [127]**	Quality thresholds	Poor quality: defined as 1 star in the selection or outcome/exposure domains, or 0 stars in the comparability domain.<br>Fair quality: defined as 2 stars in the selection domain, 1–2 stars in the comparability domain, and 2–3 stars in the outcome/exposure domain.<br>Good quality: defined as 3–4 stars in the selection domain, 1–2 stars in the comparability domain, and 2–3 stars in the outcome/exposure domain<br><u>Note:</u> Authors converted the NOS scores to align with Agency for Healthcare Research and Quality (AHRQ) thresholds for overall quality assessment.
**Berild et al. (2020) [147]**	Quality thresholds	High Quality:<br>3 or 4 stars in the selection domain<br>1 or 2 stars in the comparability domain<br>2 or 3 stars in the outcome/exposure domain<br>Moderate Quality:<br>2 stars in the selection domain<br>1 or 2 stars in the comparability domain<br>2 or 3 stars in the outcome/exposure domain<br>Low Quality:<br>0 or 1 star in the selection domain OR 0 stars in the comparability domain OR 0 or 1 star in the outcome/exposure domain<br><u>Note:</u> Authors converted the NOS scores to align with the AHRQ thresholds for overall quality assessment.
**Chen et al. (2021) [106]**	Quality thresholds; Assessment criteria	The authors included three different domains: 1. Patient selection 2. Comparability of the intervention/control group 3. Outcome assessment. Studies scoring >5 points were considered 'high quality'.
**Zhu et al. (2018) [51]**	Scoring range; Assessment criteria	The authors streamlined the NOS by excluding unvaccinated control groups, nonexposed cohort selection, and comparability assessments. This modification focused on adapted selection criteria and outcome assessment, reducing the scoring range from 0-9 to 0-6.
**Liu et al. (2021) [105]**	Quality thresholds	<u>For cohort and case-control studies:</u><br>Low: (≥7 stars)<br>Moderate: (5–6 stars)

		High: (≤4 stars)<br>Note: for cross-sectional studies, the authors assigned 1 point per item on AHRQ checklist and summed them (0–11) to classify overall risk as low (8-11), moderate (4-7), or high (0-3).
**RoB tool**	**Summary of the original tool**	
**Risk of Bias in Non-randomized Studies - of Interventions (ROBINS-I)**	Original ROBINS-I tool encompasses seven domains where bias may be introduced, including confounding, selection of participants, classification of interventions, departures from intended interventions, missing data, measurement of outcomes, and selection of reported results. The overall risk of bias can be categorized as Low, Moderate, Serious, or Critical based on responses to the signaling questions and judgments made within each domain [205].	
**Adapted in (Author, Year)**	**Adapted Aspect**	**Adaptation Details**
**Adetokunboh et al. (2019) [70]**	Risk categories	The authors adapted the risk-of-bias assessment to non-randomized studies by evaluating incomplete outcome data (attrition bias), selection of study population (selection bias), origin of data, definition of outcome, and control for confounders.
**Doyon-Plourde et al. (2019) [71]**	Context-specific assessment criteria	The authors classified bias due to confounding for health status and functional status:<br>Moderate: Requires appropriate control for both health status and functional status.<br>Serious Risk of Bias: Assigned if at least one of the confounding factors (health or functional status) is not adequately controlled.<br>Critical: Given when neither health status nor functional status is properly controlled.<br>Note: Functional status was ascertained if details about a diagnosis of dementia and/or mention of a subject's need for support in activities of daily living were mentioned.
**Tadount et al. (2020) [79]**	Context-specific assessment criteria	The authors classified bias risk in studies on sex differences in influenza vaccine responses:<br>Criteria for serious bias: Studies that failed to adjust for critical confounders, such as age and immune history. (assessed RoB for the outcomes collected, independently of those for which the study was designed)<br>Low to moderate confounding bias: Sex-stratified vaccine efficacy estimates were required to account for age, health status, and vaccination history to be considered at low or moderate risk of bias.
**Hughes et al. (2020) [80]**	Context-specific assessment criteria	The authors classified studies as 'high' risk of bias if they reported issues in the following groups:<br>Cold Chain Issues: Problems related to the storage and transportation of temperature-sensitive vaccines.<br>Width of Age Groups (i.e., age groups spanning 10 years, which may dilute specific age-related effects.)<br>Unclear or Absent Age at First Dose: Lack of clear upper and/or lower age limits for the first vaccine dose.

**Xu et al. (2021) [96]**	Context-specific assessment criteria; Scoring system	The authors classified bias due to confounding related to the presence of maternal antibodies before vaccination and/or previous measles infection.<br>Low risk: Studies that considered measles antibody levels before vaccination and/or previous measles infection.<br>Moderate risk: Studies that did not control for maternal antibodies but discussed their implications on study results.<br>Critical risk: Studies (especially cross-sectional study designs) that did not control for this confounding or discuss their implications on study results.
**Wang et al. (2021) [112]**	Context-specific assessment criteria; Scoring system	The authors assessed bias in four domains: 1. Selection Bias 2. Exposure Misclassification 3. Outcome Misclassification 4. Design Limitations. Bias in classification of the intervention was not evaluated. Deviation from intended intervention was also excluded due to the absence of an intervention being assessed.
**Markowitz et al. (2022) [140]**	Context-specific assessment criteria	The authors examined selection bias by assessing whether participant characteristics or outcomes influenced participant selection.<br>For information bias, they evaluated potential biases in:<br>Measurement of Intervention: This included the validity of data sources for determining dose groups and ensuring adequate intervals between first and second doses for two-dose recipients.<br>Measurement of Outcome: The authors reviewed the validity of algorithms used to identify outcomes and the use of lag time or buffer periods to exclude outcomes arising from pre-existing infections at the time of vaccination.<br>For confounding, the authors investigated differences between dose groups regarding:<br>Prevalence of HPV infection at the first dose<br>Risk of HPV acquisition during follow-up<br>Immunogenicity in studies comparing three, two, and one doses.
**Wu et al. (2023) [186]**	Context-specific assessment criteria	The authors indicated the tool was adapted to COVID-19, but no additional details were provided.
**Harder et al. (2021) [101]**	Study-specific assessment criteria	The authors could not apply the ROBINS-I tool to one cohort study with elements of an interrupted time series. Instead, they used the EPOC (Effective Practice and Organisation of Care) framework to assess the risk of bias across relevant domains.

**RoB tool**	**Summary of the original tool**
**Downs and Black checklist**	Original Downs and Black checklist which includes 27 items divided into four components: Quality of Reporting (10 questions), External Validity (3 questions), Internal Validity (13 questions), and Statistical Power (1 question). Each item is scored as "yes" (1 point) or "no/unable to determine" (0 points), with one item rated on a 3-point scale (yes=2, partial=1, no=0). Total scores range from 0 to 28, with higher scores indicating better quality [206].

Adapted in (Author, Year)	Adapted Aspect	Adaptation Details
**Marra et al. (2021, 2022, 2022, 2022) [123], [159], [170], [187]**	Quality thresholds	The authors followed all questions from the scale as original, except for question #27, which was modified from a score of 0 to 5 to a yes or no format. Studies were classified for analysis as:<br>Good quality: 19–23 out of 28 possible points<br>Fair quality: 14–18 out of 28 possible points
**Lee et al. (2021) [92]**	Quality thresholds	The authors replaced the numerical scoring system with a qualitative rating (poor, fair, good, excellent)
**Lee et al. (2018) [58]**	Quality thresholds	The authors reduced the number of questions from 27 to 26 (by removing or modifying the statistical power assessment) and replaced the numerical scoring system with a qualitative rating (poor, fair, good, excellent)
**RoB tool**	**Summary of the original tool**	
**Joanna Briggs Institute (JBI)**	Original JBI Checklist evaluates studies using 10-11 questions, addressing key methodological aspects, including review question clarity, inclusion criteria appropriateness, search strategy, critical appraisal process, data extraction, analysis methods, and publication bias assessment, answered as yes/no/unclear [207].	
**Adapted in (Author, Year)**	**Adapted Aspect**	**Adaptation Details**
**Mohammed et al. (2023) [191]**	Signaling questions; Response scale	The authors modified the JBI Checklist by creating a combined tool for case-control and cohort studies, featuring 9 tailored items (A-I), compared to the original 10 questions for case-control and 11 for cohort studies.
**Foucambert et al. (2022) [169]**	Response scale	The authors classified ≥8 "Yes" in a study as 'high' quality.
**RoB tool**	**Summary of the original tool**	
**Quality Assessment Tool for Studies with Diverse Designs (QATSDD)**	QATSDD includes 16 criteria focusing on key domains such as study design, sampling methods, data collection, and analysis appropriateness. Each item is rated on a 4-point scale as follows: 0 (not at all), 1 (very slightly), 2 (moderately), and 3 (completely) [208].	
**Adapted in (Author, Year)**	**Adapted Aspect**	**Adaptation Details**
**Sabu et al. (2022) [172]**	Assessment criteria	The authors modified the assessment tool by excluding three criteria deemed irrelevant for evaluating the included studies. Thirteen criteria were scored on a scale of 0 to 3 (similar to the original scale).
**RoB tool**	**Summary of the original tool**	

**National Advisory Committee on Immunization (NACI) Guidelines**	The NACI guidelines for quality assessment are based on methods from Harris et al. and the US Preventive Task Force [210].	
**Adapted in (Author, Year)**	**Adapted aspect**	**Adaptation Details**
**Berman-Rosa et al. (2020) [85]**	Context-specific quality measures	Studies lacking control for age and/or underlying medical conditions as confounders were classified as 'fair'.<br>Age was identified as a critical confounder due to:<br>Younger children's higher risk for acute otitis media (AOM) and invasive pneumococcal disease (IPD) related to their anatomy and immature immune systems<br>Potential variations in vaccination rates among different age groups based on public health strategies<br>'Fatal' flaws, determined a priori, included inadequate selection of controls in case-control studies.
**RoB tool**	**Summary of the original tool**	
**Cochrane risk of bias tool**	Original Cochrane Risk of Bias tool seven domains to focus on RCTs: random sequence generation and allocation concealment (selection bias), blinding of participants/personnel and outcome assessors (performance and detection bias), incomplete outcome data (attrition bias), selective reporting (reporting bias), and other potential sources of bias [211].	
**Adapted in (Author, Year)**	**Adapted aspect**	**Adaptation Details**
**Campbell et al. (2018) [64]**	Risk categories	The authors modified the tool by implementing a four-category system: high risk, intermediate risk, low risk, and not applicable. The 'not applicable' category was introduced for domains irrelevant to certain study designs, particularly observational studies, where aspects like random sequence generation, allocation concealment, and participant blinding are often not pertinent. To better assess multifactorial outcomes, they expanded the 'other' category into three separate domains, allowing for a more nuanced evaluation of bias implications across different domains and consideration of additional biases such as confounding in safety, immunogenicity, and effectiveness/efficacy outcomes.

Table S4. Characteristics of studies in which authors employed their own risk-of-bias assessment method (N=13)

Reference	Custom Methods	Additional Details
**Jackson et al. (2013)** [14]	Due to the relatively small number of articles identified, authors report not excluding studies based on their potential for bias but summarizing any methodological concerns in the text.	Control selection and confounding variable were assessed for bias in both case-control and cohort studies.
**Abubakar et al. (2013)** [16]	The authors developed criteria to assess bias in observational studies, categorizing risks as high, low, or unclear. Refer to pg. 30 for more details.	To assess RoB in case–control and cross-sectional studies, the authors established a criterion that included: 1) consistency in BCG vaccination definitions between cases and controls 2) blinding of disease status to assessors 3) independent diagnosis from vaccination status, and for matched studies, whether matching was included in the analysis. Cohort studies were evaluated for biases related to: 1) loss to follow-up 2) treatment allocation concealment 3) case ascertainment.
**Darvishian et al. (2014)** [22]	The authors created their own scale and checklist to identify potential internal and external biases. To quantify biases, they created an elicitation scale for additive and proportional biases. See supplementary materials.	For internal biases, the authors focused on selection, performance, attrition, detection, and other suspected biases.<br>For external bias, they compared study populations to the target population in terms of age, sex, and health status.
**Remschmidt et al. (2015)** [29]	The authors used a predefined criteria from a methodological framework to assess the risk of healthy vaccinee bias and confounding by indication in the included studies. See Table 1.	'High risk' of healthy vaccinee bias, if vaccinated participants exhibited significantly fewer comorbidities or related indicators, such as medical visits, compared to unvaccinated participants based on baseline characteristics.<br>'High risk' of confounding by indication if vaccinated participants had significantly more comorbidities than their unvaccinated counterparts.
**Garland et al. (2016)** [33]	The authors detailed the strengths and limitations of each study included in the formal analysis. See Supplementary Table 2.	Not reported
**Caspard et al. (2017)** [38]	See Table 1.	The quality of evidence was examined based on the study eligibility criteria and age range.<br>To account for the risk of bias associated with the designs of observational studies, they adjusted for VE estimates using the following formula: 100×(1−consolidated odds ratio or relative risk).
**Markowitz et al. (2018)** [60]	The authors reported extracting information on how potential biases were addressed in the included studies. Accordingly, no studies were excluded based on methodological quality.	The sources of bias in post-licensure studies examining the impact of HPV vaccination by the number of doses include: (1) differences in characteristics and age at vaccination; (2) the likelihood of prevalent infection at the time of vaccination; and (3) the interval between the first and second doses for those receiving two doses.
**Yakely et al. (2019)** [67]	The authors assessed the quality of effectiveness using their own scale. See Supp 2.	Quality of effectiveness studies were evaluated for: Selection of cohort; Ascertainment of exposure; Control of other factors; Assessment of outcome; Adequacy of follow-up
**Drolet et al. (2019)** [77]	The authors created their own criteria to assess methodological quality of included studies. See Appendix.	Methodological quality of included studies was assessed for:<br>Risk of selection bias:<br>- By examining changes in the study population characteristics between the pre- and post-vaccination periods.<br>Risk of information bias:<br>- By considering errors in the identification of pre-cancerous cervical lesions during the pre- and post-vaccination periods.

Reference	Custom Methods	Additional Details
		Risk of confounding:<br>- By examining whether changes in precancerous lesions between pre- and post-vaccination periods could be diluted or exacerbated by other variables.<br>External validity:<br>- By determining whether results could be generalized to the population at the country or region level.
**Andani et al. (2022)** [108]	The authors developed an in-house tool to critically appraise the methodological quality of articles reporting real-world studies. See Supplementary Text S2.	The authors developed an in-house quality assessment tool with three sections (a total of 6 questions):<br>A. Case Detection<br>B. Data Collection Methods<br>C. Quantitative Methods<br>The rating system used four categories: Weak, Moderate, Strong, and Fatal Error. If a study received a "Fatal Error" rating, it was excluded from the review.
**Okoli et al. (2021)** [151]	The authors developed a quality assessment approach for test-negative design (TND) studies in the absence of a validated tool.<br>See Supplementary Table 4.	The authors examined relevant study characteristics that could introduce bias, including:<br>- Patient recruitment into study<br>- Influenza vaccination confirmation methods<br>- Inclusion of age and comorbidity among covariates adjusted in logistic regression analysis for VE<br>These characteristics were synthesized in a tabular form.
**Luna et al. (2014)** [203]	The authors assessed the quality of included studies based on several criteria.	- Studies with laboratory-confirmed influenza cases were favored.<br>- Representative, randomly selected samples were considered higher quality in vaccination coverage surveys.<br>- Methods accounting for seasonal and cyclical variability were preferred in ecological time series studies.<br>- Quality was judged on geographic scope, time period analyzed, and statistical methods that incorporated seasonal disease variability in ecological historical series.
**Kandeil et al. (2020)** [87]*	The quality of studies reporting population-based outcomes was assessed using an in-house quality assessment tool. See section 3.0 online supplemental file.	Articles with a "fatal error" (a factor undermining the study's main conclusions) were excluded, while all others were included regardless of their overall rating (strong, moderate, or weak). Studies focused on patient follow-up were not evaluated with this tool or existing checklists and were therefore not critically appraised.

***Kandeil et al. (2020)** [87] planned to use the GRADE quality assessment tool for vaccine effectiveness studies but found it unsuitable due to varying study outcomes and the need for data recalculation.

Table S5. Summary of quality assessment methods used in other reports identified through grey literature sources (N=9)

Document	Appraisal Method	Description
CoVaRR-Net/ COVID-END: COVID-19 Living Evidence Synthesis (n=4 versions using the same tool) [212]	Adapted version of ROBINS-I	The adapted version of ROBINS-I tailors the tool specifically for vaccine research by incorporating the following study characteristics unique to this field that could potentially introduce bias.<br>1. Study design<br>2. Method for confirming vaccination<br>3. Databases used for retrieval of COVID test results, participant prognostic factors, and clinical outcomes<br>4. Assignment of infection start date<br>5. Verification of symptoms<br>6. Accounting for nonimmune period<br>7. Inclusion of participants with prior COVID infection<br>8. Accounting for calendar time<br>9. Adjustment for prognostic factors<br>10. Testing frequency<br>Overall "serious" or "critical" judgment given when critical risk in one domain or serious risk in three or more domains. See Appendix 5 for further details.
SPOR/COVID-END: What is the ongoing effectiveness, immunogenicity, and safety of COVID-19 vaccines in persons who have had a prior, confirmed COVID-19 infection? [213]	JBI Checklist	JBI Checklist for Cohort Studies<br>JBI Checklist for Analytical Cross-Sectional Studies<br>Further details about the tool application not available.
SPOR/COVID-END: Transmissibility of COVID-19 among vaccinated individuals [214]	ROBINS-I	RoB was assessed in five non-RCTs. Results shown in Figure 2.
NACI: Repeated Seasonal Influenza Vaccination [215]	AMSTAR-2 for SRs/MAs	The adapted version of AMSTAR 2 differs from its original design in approach to scoring. While the original AMSTAR 2 tool provides an overall qualitative rating based on weaknesses in critical domains without generating a numerical score, the authors converted checklist answers into a numerical scoring system rated out of 16 total points. Results shown in Table 3.
NACI: Updated Recommendations on the Use of Herpes Zoster Vaccines [216]	NOS<br>EPOC	The Newcastle Ottawa Quality Assessment Scale for cohort studies and case-control studies<br>The EPOC Risk of Bias tool for nonrandomized controlled trials.<br>Results shown in Appendix A.
ECDC: Systematic review of the efficacy, effectiveness and safety of newer and enhanced seasonal influenza vaccines for the prevention	ROBINS-I<br>GRADE	ROBINS-I was adapted to evaluate bias in non-randomized vaccine studies across seven domains, with overall risk determined by the highest domain score. GRADE assessed evidence quality for primary outcomes, considering five factors without automatically downgrading non-randomized studies instead treating as high-certainty evidence, similar to RCTs. The review focused on test-

Document	Appraisal Method	Description
of laboratory-confirmed influenza in individuals aged 18 years and over [217]		negative design case-control studies for primary effectiveness of laboratory-confirmed influenza and case-control and cohort studies for additional outcomes. Results shown in Table 3.3 and 'summary of findings' table.

Abbreviations: ROBINS-I (Risk Of Bias In Non-randomized Studies - of Interventions), JBI Checklist (Joanna Briggs Institute Checklist), AMSTAR-2 (A MeaSurement Tool to Assess systematic Reviews 2), NOS (Newcastle-Ottawa Scale), EPOC (Effective Practice and Organisation of Care), GRADE (Grading of Recommendations Assessment, Development and Evaluation)

Bibliography

[1] D. R. Feikin, M. H. Melissa, and L. J. Abu-Raddad, "Duration of effectiveness of vaccines against SARS-CoV-2 infection and COVID-19 disease: results of a systematic review and metaregression (vol 399, pg 924, 2022)," *Lancet*, vol. 401, no. 10377, pp. 644–644, 2023, [Online]. Available: ://WOS:000947978900002

[2] Z. W. Sun *et al.*, "Association of Rotavirus Vaccines With Reduction in Rotavirus Gastroenteritis in Children Younger Than 5 Years A Systematic Review and Meta-analysis of Randomized Clinical Trials and Observational Studies," *JAMA Pediatr.*, vol. 175, no. 7, p. 13, 2021, doi: 10.1001/jamapediatrics.2021.0347.

[3] L. C. Ramsay *et al.*, "The impact of repeated vaccination on influenza vaccine effectiveness: a systematic review and meta-analysis," *BMC Med.*, vol. 17, p. 16, 2019, doi: 10.1186/s12916-018-1239-8.

[4] H. Q. Wang, Y. M. Hu, G. M. Zhang, J. S. Zheng, L. Li, and Z. J. An, "Meta-analysis of vaccine effectiveness of mumps-containing vaccine under different immunization srategies in China," *Vaccine*, vol. 32, no. 37, pp. 4806–4812, 2014, doi: 10.1016/j.vaccine.2014.05.061.

[5] A. Roy *et al.*, "Effect of BCG vaccination against Mycobacterium tuberculosis infection in children: systematic review and meta-analysis," *BMJ-British Medical Journal*, vol. 349, p. 11, 2014, doi: 10.1136/bmj.g4643.

[6] M. Pal, F. Goodyear-Smith, and D. Exeter, "Systematic review of pertussis immunisation among Asians," *Int. J. Hum. Rights Health Care*, vol. 9, no. 2, pp. 135–146, 2016, doi: 10.1108/ijhrh-02-2016-0002.

[7] M. McMillan, D. Kralik, K. Porritt, and H. Marshall, "Influenza vaccination during pregnancy: A systematic review of effectiveness and safety," *JBI Database of Systematic Reviews and Implementation Reports*, vol. 12, no. 6, pp. 281–381, 2014, doi: 10.11124/jbisrir-2014-1269.

[8] A. S. Alqahtani, H. Rashid, and A. E. Heywood, "Vaccinations against respiratory tract infections at Hajj," *Clinical Microbiology and Infection*, vol. 21, no. 2, pp. 115–127, 2015, doi: 10.1016/j.cmi.2014.11.026.

[9] M. Tormen *et al.*, "Effectiveness and safety of COVID-19 vaccine in pregnant women: a systematic review with meta-analysis," *BJOG : an international journal of obstetrics and gynaecology*, 2022, doi: 10.1111/1471-0528.17354.

[10] E. Goodman, M. Reuschenbach, A. Kaminski, and S. Ronnebaum, "Human Papillomavirus Vaccine Impact and Effectiveness in Six High-Risk Populations: A Systematic Literature Review," *Vaccines*, vol. 10, no. 9, p. 1543, 2022, doi: 10.3390/vaccines10091543.

[11] F. Andersohn, R. Bornemann, O. Damm, M. Frank, T. Mittendorf, and U. Theidel, "Vaccination of children with a live-attenuated, intranasal influenza vaccine - analysis and evaluation through a Health Technology Assessment," *GMS health technology assessment*, vol. 10, p. Doc03, 2014, doi: 10.3205/hta000119.

[12] S. Moberley, J. Holden, D. P. Tatham, and R. M. Andrews, "Vaccines for preventing pneumococcal infection in adults," *The Cochrane database of systematic reviews*, no. 1, p. CD000422, 2013, doi: 10.1002/14651858.CD000422.pub3.

[13] I. Luksic *et al.*, "Effectiveness of seasonal influenza vaccines in children -- a systematic review and meta-analysis," *Croatian medical journal*, vol. 54, no. 2, pp. 135–45, 2013, [Online]. Available: http://ovidsp.ovid.com/ovidweb.cgi?T=JS&PAGE=reference&D=med10&NEWS=N&AN=23630141

[14] C. Jackson, A. Mann, P. Mangtani, and P. Fine, "Effectiveness of Haemophilus influenzae type b vaccines administered according to various schedules: systematic review and meta-analysis of observational data," *The Pediatric infectious disease journal*, vol. 32, no. 11, pp. 1261–9, 2013, doi: 10.1097/INF.0b013e3182a14e57.

[15] J. K. Breteler, J. S. Tam, M. Jit, J. C. F. Ket, and M. R. De Boer, "Efficacy and effectiveness of seasonal and pandemic A (H1N1) 2009 influenza vaccines in low and middle income countries: a systematic review and meta-analysis," *Vaccine*, vol. 31, no. 45, pp. 5168–77, 2013, doi: 10.1016/j.vaccine.2013.08.056.

[16] I. Abubakar *et al.*, "Systematic review and meta-analysis of the current evidence on the duration of protection by bacillus Calmette-Guerin vaccination against tuberculosis," *Health technology assessment (Winchester, England)*, vol. 17, no. 37, pp. 1–vi, 2013, doi: 10.3310/hta17370.

[17] N. Eliakim-Raz, I. Vinograd, A. Zalmanovici Trestioreanu, L. Leibovici, and M. Paul, "Influenza vaccines in immunosuppressed adults with cancer," *The Cochrane database of systematic reviews*, no. 10, p. CD008983, 2013, doi: 10.1002/14651858.CD008983.pub2.

[18] S. Davis, D. Feikin, and H. L. Johnson, "The effect of Haemophilus influenzae type B and pneumococcal conjugate vaccines on childhood meningitis mortality: a systematic review," *BMC public health*, vol. 13 Suppl 3, p. S21, 2013, doi: 10.1186/1471-2458-13-S3-S21.

[19] J. K. Das, A. Tripathi, A. Ali, A. Hassan, C. Dojosoeandy, and Z. A. Bhutta, "Vaccines for the prevention of diarrhea due to cholera, shigella, ETEC and rotavirus," *BMC public health*, vol. 13 Suppl 3, p. S11, 2013, doi: 10.1186/1471-2458-13-S3-S11.

[20] T. J. O'Neill, J. M. Sargeant, and Z. Poljak, "The effectiveness of Coxiella burnetii vaccines in occupationally exposed populations: a systematic review and meta-analysis," *Zoonoses and public health*, vol. 61, no. 2, pp. 81–96, 2014, doi: 10.1111/zph.12054.

[21] T.-C. Chan, I. Fan-Ngai Hung, J. Ka-Hay Luk, L.-W. Chu, and F. Hon-Wai Chan, "Effectiveness of influenza vaccination in institutionalized older adults: a systematic review," *Journal of the American Medical Directors Association*, vol. 15, no. 3, p. 226.e1-226.e6, 2014, doi: 10.1016/j.jamda.2013.10.008.

[22] M. Darvishian *et al.*, “After adjusting for bias in meta-analysis seasonal influenza vaccine remains effective in community-dwelling elderly,” *Journal of clinical epidemiology*, vol. 67, no. 7, pp. 734–44, 2014, doi: 10.1016/j.jclinepi.2014.02.009.

[23] C. Remschmidt, O. Wichmann, and T. Harder, “Influenza vaccination in HIV-infected individuals: systematic review and assessment of quality of evidence related to vaccine efficacy, effectiveness and safety,” *Vaccine*, vol. 32, no. 43, pp. 5585–92, 2014, doi: 10.1016/j.vaccine.2014.07.101.

[24] C. Remschmidt, O. Wichmann, and T. Harder, “Influenza vaccination in patients with end-stage renal disease: systematic review and assessment of quality of evidence related to vaccine efficacy, effectiveness, and safety,” *BMC medicine*, vol. 12, p. 244, 2014, doi: 10.1186/s12916-014-0244-9.

[25] M. Machaira, V. Papaevangelou, E. K. Vouloumanou, G. S. Tansarli, and M. E. Falagas, “Hepatitis B vaccine alone or with hepatitis B immunoglobulin in neonates of HBsAg+/HBeAg- mothers: a systematic review and meta-analysis,” *The Journal of antimicrobial chemotherapy*, vol. 70, no. 2, pp. 396–404, 2015, doi: 10.1093/jac/dku404.

[26] Z. Li, J. Chen, Y. Zhang, and W. Fu, “Partial protection against 2009 pandemic influenza A (H1N1) of seasonal influenza vaccination and related regional factors: Updated systematic review and meta-analyses,” *Human vaccines & immunotherapeutics*, vol. 11, no. 6, pp. 1337–44, 2015, doi: 10.4161/21645515.2014.985495.

[27] C. Remschmidt, O. Wichmann, and T. Harder, “Vaccines for the prevention of seasonal influenza in patients with diabetes: systematic review and meta-analysis,” *BMC medicine*, vol. 13, p. 53, 2015, doi: 10.1186/s12916-015-0295-6.

[28] L. H. de Oliveira, L. A. B. Camacho, E. S. F. Coutinho, C. Ruiz-Matus, and J. P. G. Leite, “Rotavirus vaccine effectiveness in Latin American and Caribbean countries: A systematic review and meta-analysis,” *Vaccine*, vol. 33 Suppl 1, pp. A248-54, 2015, doi: 10.1016/j.vaccine.2014.11.060.

[29] C. Remschmidt, O. Wichmann, and T. Harder, “Frequency and impact of confounding by indication and healthy vaccinee bias in observational studies assessing influenza vaccine effectiveness: a systematic review,” *BMC infectious diseases*, vol. 15, p. 429, 2015, doi: 10.1186/s12879-015-1154-y.

[30] S. Hirve, P. Lambach, J. Paget, K. Vandemaele, J. Fitzner, and W. Zhang, “Seasonal influenza vaccine policy, use and effectiveness in the tropics and subtropics - a systematic literature review,” *Influenza and other respiratory viruses*, vol. 10, no. 4, pp. 254–67, 2016, doi: 10.1111/irv.12374.

[31] H. Kraicer-Melamed, S. O’Donnell, and C. Quach, “The effectiveness of pneumococcal polysaccharide vaccine 23 (PPV23) in the general population of 50 years of age and older: A systematic review and meta-analysis,” *Vaccine*, vol. 34, no. 13, pp. 1540–1550, 2016, doi: 10.1016/j.vaccine.2016.02.024.

[32] T. R. Fulton, V. K. Phadke, W. A. Orenstein, A. R. Hinman, W. D. Johnson, and S. B. Omer, “Protective Effect of Contemporary Pertussis Vaccines: A Systematic Review and Meta-analysis,” *Clinical infectious diseases : an official publication of the Infectious Diseases Society of America*, vol. 62, no. 9, pp. 1100–1110, 2016, doi: 10.1093/cid/ciw051.

[33] S. M. Garland *et al.*, “Impact and Effectiveness of the Quadrivalent Human Papillomavirus Vaccine: A Systematic Review of 10 Years of Real-world Experience,” *Clinical infectious diseases : an official publication of the Infectious Diseases Society of America*, vol. 63, no. 4, pp. 519–27, 2016, doi: 10.1093/cid/ciw354.

[34] L. M. Lamberti, S. Ashraf, C. L. F. Walker, and R. E. Black, “A Systematic Review of the Effect of Rotavirus Vaccination on Diarrhea Outcomes Among Children Younger Than 5 Years,” *The Pediatric infectious disease journal*, vol. 35, no. 9, pp. 992–8, 2016, doi: 10.1097/INF.0000000000001232.

[35] L. Casanova, N. Gobin, P. Villani, and P. Verger, “Bias in the measure of the effectiveness of seasonal influenza vaccination among diabetics,” *Primary care diabetes*, vol. 10, no. 6, pp. 398–406, 2016, doi: 10.1016/j.pcd.2016.05.005.

[36] V. S. Santos, D. P. Marques, P. R. S. Martins-Filho, L. E. Cuevas, and R. Q. Gurgel, “Effectiveness of rotavirus vaccines against rotavirus infection and hospitalization in Latin America: systematic review and meta-analysis,” *Infectious diseases of poverty*, vol. 5, no. 1, p. 83, 2016, doi: 10.1186/s40249-016-0173-2.

[37] Y.-Y. Zhang, X.-F. Tang, C.-H. Du, B.-B. Wang, Z.-W. Bi, and B.-R. Dong, “Comparison of dual influenza and pneumococcal polysaccharide vaccination with influenza vaccination alone for preventing pneumonia and reducing mortality among the elderly: A meta-analysis,” *Human vaccines & immunotherapeutics*, vol. 12, no. 12, pp. 3056–3064, 2016, doi: 10.1080/21645515.2016.1221552.

[38] H. Caspard, R. M. Mallory, J. Yu, and C. S. Ambrose, “Live-Attenuated Influenza Vaccine Effectiveness in Children From 2009 to 2015-2016: A Systematic Review and Meta-Analysis,” *Open forum infectious diseases*, vol. 4, no. 3, p. ofx111, 2017, doi: 10.1093/ofid/ofx111.

[39] C. Willame *et al.*, “Effectiveness of the Oral Human Attenuated Rotavirus Vaccine: A Systematic Review and Meta-analysis-2006-2016,” *Open forum infectious diseases*, vol. 5, no. 11, p. ofy292, 2018, doi: 10.1093/ofid/ofy292.

[40] S. Poudel *et al.*, “The Effect of Influenza Vaccination on Mortality and Risk of Hospitalization in Patients With Heart Failure: A Systematic Review and Meta-analysis,” *Open forum infectious diseases*, vol. 6, no. 4, p. ofz159, 2019, doi: 10.1093/ofid/ofz159.

[41] A. Domnich, L. Arata, D. Amicizia, J. Puig-Barbera, R. Gasparini, and D. Panatto, “Effectiveness of MF59-adjuvanted seasonal influenza vaccine in the elderly: A systematic review and meta-analysis,” *Vaccine*, vol. 35, no. 4, pp. 513–520, 2017, doi: 10.1016/j.vaccine.2016.12.011.

[42] G. Falkenhorst, C. Remschmidt, T. Harder, E. Hummers-Pradier, O. Wichmann, and C. Bogdan, "Effectiveness of the 23-Valent Pneumococcal Polysaccharide Vaccine (PPV23) against Pneumococcal Disease in the Elderly: Systematic Review and Meta-Analysis," *PloS one*, vol. 12, no. 1, p. e0169368, 2017, doi: 10.1371/journal.pone.0169368.
[43] L. E. Lansbury *et al.*, "Effectiveness of 2009 pandemic influenza A(H1N1) vaccines: A systematic review and meta-analysis," *Vaccine*, vol. 35, no. 16, pp. 1996–2006, 2017, doi: 10.1016/j.vaccine.2017.02.059.
[44] R. Bekkat-Berkani *et al.*, "Seasonal influenza vaccination in patients with COPD: a systematic literature review," *BMC pulmonary medicine*, vol. 17, no. 1, p. 79, 2017, doi: 10.1186/s12890-017-0420-8.
[45] M. Tin Tin Htar *et al.*, "Effectiveness of pneumococcal vaccines in preventing pneumonia in adults, a systematic review and meta-analyses of observational studies," *PloS one*, vol. 12, no. 5, p. e0177985, 2017, doi: 10.1371/journal.pone.0177985.
[46] E. Vasileiou *et al.*, "Effectiveness of Influenza Vaccines in Asthma: A Systematic Review and Meta-Analysis," *Clinical infectious diseases : an official publication of the Infectious Diseases Society of America*, vol. 65, no. 8, pp. 1388–1395, 2017, doi: 10.1093/cid/cix524.
[47] Q. Bi *et al.*, "Protection against cholera from killed whole-cell oral cholera vaccines: a systematic review and meta-analysis," *The Lancet. Infectious diseases*, vol. 17, no. 10, pp. 1080–1088, 2017, doi: 10.1016/S1473-3099(17)30359-6.
[48] D. Hungerford *et al.*, "Population effectiveness of the pentavalent and monovalent rotavirus vaccines: a systematic review and meta-analysis of observational studies," *BMC infectious diseases*, vol. 17, no. 1, p. 569, 2017, doi: 10.1186/s12879-017-2613-4.
[49] M. Furuta, J. Sin, E. S. W. Ng, and K. Wang, "Efficacy and safety of pertussis vaccination for pregnant women - a systematic review of randomised controlled trials and observational studies," *BMC pregnancy and childbirth*, vol. 17, no. 1, p. 390, 2017, doi: 10.1186/s12884-017-1559-2.
[50] V. Restivo *et al.*, "Influenza vaccine effectiveness among high-risk groups: A systematic literature review and meta-analysis of case-control and cohort studies," *Human vaccines & immunotherapeutics*, vol. 14, no. 3, pp. 724–735, 2018, doi: 10.1080/21645515.2017.1321722.
[51] S. Zhu, F. Zeng, L. Xia, H. He, and J. Zhang, "Incidence rate of breakthrough varicella observed in healthy children after 1 or 2 doses of varicella vaccine: Results from a meta-analysis," *American journal of infection control*, vol. 46, no. 1, pp. e1–e7, 2018, doi: 10.1016/j.ajic.2017.07.029.
[52] B. Young, S. Sadarangani, L. Jiang, A. Wilder-Smith, and M. I. C. Chen, "Duration of Influenza Vaccine Effectiveness: A Systematic Review, Meta-analysis, and Meta-regression of Test-Negative Design Case-Control Studies," *The Journal of infectious diseases*, vol. 217, no. 5, pp. 731–741, 2018, doi: 10.1093/infdis/jix632.
[53] P. Schwerdtle, C.-K. Onekon, and K. Recoche, "A Quantitative Systematic Review and Meta-Analysis of the Effectiveness of Oral Cholera Vaccine as a Reactive Measure in Cholera Outbreaks," *Prehospital and disaster medicine*, vol. 33, no. 1, pp. 2–6, 2018, doi: 10.1017/S1049023X17007166.
[54] M. Yin, X. Xu, Y. Liang, and J. Ni, "Effectiveness, immunogenicity and safety of one vs. two-dose varicella vaccination:a meta-analysis," *Expert review of vaccines*, vol. 17, no. 4, pp. 351–362, 2018, doi: 10.1080/14760584.2018.1433999.
[55] R. Bitterman, N. Eliakim-Raz, I. Vinograd, A. Zalmanovici Trestioreanu, L. Leibovici, and M. Paul, "Influenza vaccines in immunosuppressed adults with cancer," *The Cochrane database of systematic reviews*, vol. 2, p. CD008983, 2018, doi: 10.1002/14651858.CD008983.pub3.
[56] G. Dos Santos, H. Tahrat, and R. Bekkat-Berkani, "Immunogenicity, safety, and effectiveness of seasonal influenza vaccination in patients with diabetes mellitus: A systematic review," *Human vaccines & immunotherapeutics*, vol. 14, no. 8, pp. 1853–1866, 2018, doi: 10.1080/21645515.2018.1446719.
[57] P. Zimmermann, A. Finn, and N. Curtis, "Does BCG Vaccination Protect Against Nontuberculous Mycobacterial Infection? A Systematic Review and Meta-Analysis," *The Journal of infectious diseases*, vol. 218, no. 5, pp. 679–687, 2018, doi: 10.1093/infdis/jiy207.
[58] J. K. H. Lee *et al.*, "Efficacy and effectiveness of high-dose versus standard-dose influenza vaccination for older adults: a systematic review and meta-analysis," *Expert review of vaccines*, vol. 17, no. 5, pp. 435–443, 2018, doi: 10.1080/14760584.2018.1471989.
[59] J. J. Bartoszko *et al.*, "Does consecutive influenza vaccination reduce protection against influenza: A systematic review and meta-analysis," *Vaccine*, vol. 36, no. 24, pp. 3434–3444, 2018, doi: 10.1016/j.vaccine.2018.04.049.
[60] L. E. Markowitz, M. Drolet, N. Perez, M. Jit, and M. Brisson, "Human papillomavirus vaccine effectiveness by number of doses: Systematic review of data from national immunization programs," *Vaccine*, vol. 36, no. 32 Pt A, pp. 4806–4815, 2018, doi: 10.1016/j.vaccine.2018.01.057.
[61] A. Chit *et al.*, "Acellular pertussis vaccines effectiveness over time: A systematic review, meta-analysis and modeling study," *PloS one*, vol. 13, no. 6, p. e0197970, 2018, doi: 10.1371/journal.pone.0197970.
[62] M. Yin *et al.*, "Effectiveness and safety of dual influenza and pneumococcal vaccination versus separate administration or no vaccination in older adults: a meta-analysis," *Expert review of vaccines*, vol. 17, no. 7, pp. 653–663, 2018, doi: 10.1080/14760584.2018.1495077.
[63] T. Harder, O. Wichmann, S. J. Klug, M. A. B. van der Sande, and M. Wiese-Posselt, "Efficacy, effectiveness and safety of vaccination against human papillomavirus in males: a systematic review," *BMC medicine*, vol. 16, no. 1, p. 110, 2018, doi: 10.1186/s12916-018-1098-3.

[64] H. Campbell *et al.*, “Review of vaccination in pregnancy to prevent pertussis in early infancy,” *Journal of medical microbiology*, vol. 67, no. 10, pp. 1426–1456, 2018, doi: 10.1099/jmm.0.000829.
[65] A. C. Tricco *et al.*, “Efficacy, effectiveness, and safety of herpes zoster vaccines in adults aged 50 and older: systematic review and network meta-analysis,” *BMJ (Clinical research ed.)*, vol. 363, p. k4029, 2018, doi: 10.1136/bmj.k4029.
[66] H. L. Sings *et al.*, “Effectiveness of 13-Valent Pneumococcal Conjugate Vaccine Against Invasive Disease Caused by Serotype 3 in Children: A Systematic Review and Meta-analysis of Observational Studies,” *Clinical infectious diseases : an official publication of the Infectious Diseases Society of America*, vol. 68, no. 12, pp. 2135–2143, 2019, doi: 10.1093/cid/ciy920.
[67] A. E. Yakely, L. Avni-Singer, C. R. Oliveira, and L. M. Niccolai, “Human Papillomavirus Vaccination and Anogenital Warts: A Systematic Review of Impact and Effectiveness in the United States,” *Sexually transmitted diseases*, vol. 46, no. 4, pp. 213–220, 2019, doi: 10.1097/OLQ.0000000000000948.
[68] J. S. Ngocho *et al.*, “Effectiveness of pneumococcal conjugate vaccines against invasive pneumococcal disease among children under five years of age in Africa: A systematic review,” *PloS one*, vol. 14, no. 2, p. e0212295, 2019, doi: 10.1371/journal.pone.0212295.
[69] H. Senderovich, J. Grewal, and M. Mujtaba, “Herpes zoster vaccination efficacy in the long-term care facility population: a qualitative systematic review,” *Current medical research and opinion*, vol. 35, no. 8, pp. 1451–1462, 2019, doi: 10.1080/03007995.2019.1600482.
[70] O. O. Adetokunboh, D. Ndwandwe, A. Awotiwon, O. A. Uthman, and C. S. Wiysonge, “Vaccination among HIV-infected, HIV-exposed uninfected and HIV-uninfected children: a systematic review and meta-analysis of evidence related to vaccine efficacy and effectiveness,” *Human vaccines & immunotherapeutics*, vol. 15, no. 11, pp. 2578–2589, 2019, doi: 10.1080/21645515.2019.1599677.
[71] P. Doyon-Plourde, I. Fakih, F. Tadount, E. Fortin, and C. Quach, “Impact of influenza vaccination on healthcare utilization - A systematic review,” *Vaccine*, vol. 37, no. 24, pp. 3179–3189, 2019, doi: 10.1016/j.vaccine.2019.04.051.
[72] H. M. Vardanjani, H. Borna, and A. Ahmadi, “Effectiveness of pneumococcal conjugate vaccination against invasive pneumococcal disease among children with and those without HIV infection: a systematic review and meta-analysis,” *BMC infectious diseases*, vol. 19, no. 1, p. 685, 2019, doi: 10.1186/s12879-019-4325-4.
[73] S. Harmala, C. A. Parisinos, L. Shallcross, A. O’Brien, and A. Hayward, “Effectiveness of influenza vaccines in adults with chronic liver disease: a systematic review and meta-analysis,” *BMJ open*, vol. 9, no. 9, p. e031070, 2019, doi: 10.1136/bmjopen-2019-031070.
[74] L. M. Nic Lochlainn *et al.*, “Immunogenicity, effectiveness, and safety of measles vaccination in infants younger than 9 months: a systematic review and meta-analysis,” *The Lancet. Infectious diseases*, vol. 19, no. 11, pp. 1235–1245, 2019, doi: 10.1016/S1473-3099(19)30395-0.
[75] L. M. Nic Lochlainn *et al.*, “Effect of measles vaccination in infants younger than 9 months on the immune response to subsequent measles vaccine doses: a systematic review and meta-analysis,” *The Lancet. Infectious diseases*, vol. 19, no. 11, pp. 1246–1254, 2019, doi: 10.1016/S1473-3099(19)30396-2.
[76] L. Friedman *et al.*, “Exploring indirect protection associated with influenza immunization - A systematic review of the literature,” *Vaccine*, vol. 37, no. 49, pp. 7213–7232, 2019, doi: 10.1016/j.vaccine.2019.09.086.
[77] M. Drolet, E. Benard, N. Perez, and M. Brisson, “Population-level impact and herd effects following the introduction of human papillomavirus vaccination programmes: updated systematic review and meta-analysis,” *Lancet (London, England)*, vol. 394, no. 10197, pp. 497–509, 2019, doi: 10.1016/S0140-6736(19)30298-3.
[78] B. B. Lindsey, E. P. Armitage, B. Kampmann, and T. I. de Silva, “The efficacy, effectiveness, and immunogenicity of influenza vaccines in Africa: a systematic review,” *The Lancet. Infectious diseases*, vol. 19, no. 4, pp. e110–e119, 2019, doi: 10.1016/S1473-3099(18)30490-0.
[79] F. Tadount, P. Doyon-Plourde, E. Rafferty, S. MacDonald, M. Sadarangani, and C. Quach, “Is there a difference in the immune response, efficacy, effectiveness and safety of seasonal influenza vaccine in males and females? - A systematic review,” *Vaccine*, vol. 38, no. 3, pp. 444–459, 2020, doi: 10.1016/j.vaccine.2019.10.091.
[80] S. L. Hughes *et al.*, “The effect of time since measles vaccination and age at first dose on measles vaccine effectiveness - A systematic review,” *Vaccine*, vol. 38, no. 3, pp. 460–469, 2020, doi: 10.1016/j.vaccine.2019.10.090.
[81] T. H. T. Quach, N. A. Mallis, and J. F. Cordero, “Influenza Vaccine Efficacy and Effectiveness in Pregnant Women: Systematic Review and Meta-analysis,” *Maternal and child health journal*, vol. 24, no. 2, pp. 229–240, 2020, doi: 10.1007/s10995-019-02844-y.
[82] J. R. Jarvis, R. B. Dorey, F. D. M. Warricker, N. A. Alwan, and C. E. Jones, “The effectiveness of influenza vaccination in pregnancy in relation to child health outcomes: Systematic review and meta-analysis,” *Vaccine*, vol. 38, no. 7, pp. 1601–1613, 2020, doi: 10.1016/j.vaccine.2019.12.056.
[83] S. Vygen-Bonnet *et al.*, “Safety and effectiveness of acellular pertussis vaccination during pregnancy: a systematic review,” *BMC infectious diseases*, vol. 20, no. 1, p. 136, 2020, doi: 10.1186/s12879-020-4824-3.
[84] M. Kalligeros *et al.*, “Influenza vaccine effectiveness against influenza-associated hospitalization in children: A systematic review and meta-analysis,” *Vaccine*, vol. 38, no. 14, pp. 2893–2903, 2020, doi: 10.1016/j.vaccine.2020.02.049.

[85] M. Berman-Rosa, S. O'Donnell, M. Barker, and C. Quach, "Efficacy and Effectiveness of the PCV-10 and PCV-13 Vaccines Against Invasive Pneumococcal Disease," *Pediatrics*, vol. 145, no. 4, 2020, doi: 10.1542/peds.2019-0377.
[86] C. Di Pietrantonj, A. Rivetti, P. Marchione, M. G. Debalini, and V. Demicheli, "Vaccines for measles, mumps, rubella, and varicella in children," *The Cochrane database of systematic reviews*, vol. 4, p. CD004407, 2020, doi: 10.1002/14651858.CD004407.pub4.
[87] W. Kandeil, C. van den Ende, E. M. Bunge, V. A. Jenkins, M. A. Ceregido, and A. Guignard, "A systematic review of the burden of pertussis disease in infants and the effectiveness of maternal immunization against pertussis," *Expert review of vaccines*, vol. 19, no. 7, pp. 621–638, 2020, doi: 10.1080/14760584.2020.1791092.
[88] K. Yitbarek, G. Abraham, T. Girma, T. Tilahun, and M. Woldie, "The effect of Bacillus Calmette-Guerin (BCG) vaccination in preventing severe infectious respiratory diseases other than TB: Implications for the COVID-19 pandemic," *Vaccine*, vol. 38, no. 41, pp. 6374–6380, 2020, doi: 10.1016/j.vaccine.2020.08.018.
[89] Y. Mo *et al.*, "Effectiveness and safety of pneumococcal vaccines used alone or combined with influenza vaccination in dialysis patients: A systematic review and meta-analysis," *Vaccine*, vol. 38, no. 47, pp. 7422–7432, 2020, doi: 10.1016/j.vaccine.2020.09.080.
[90] M. Zangiabadian *et al.*, "Protective effect of influenza vaccination on cardiovascular diseases: a systematic review and meta-analysis," *Scientific reports*, vol. 10, no. 1, p. 20656, 2020, doi: 10.1038/s41598-020-77679-7.
[91] M. McMillan *et al.*, "Effectiveness of Meningococcal Vaccines at Reducing Invasive Meningococcal Disease and Pharyngeal Neisseria meningitidis Carriage: A Systematic Review and Meta-analysis," *Clinical infectious diseases : an official publication of the Infectious Diseases Society of America*, vol. 73, no. 3, pp. e609–e619, 2021, doi: 10.1093/cid/ciaa1733.
[92] J. K. H. Lee, G. K. L. Lam, T. Shin, S. I. Samson, D. P. Greenberg, and A. Chit, "Efficacy and effectiveness of high-dose influenza vaccine in older adults by circulating strain and antigenic match: An updated systematic review and meta-analysis," *Vaccine*, vol. 39 Suppl 1, pp. A24–A35, 2021, doi: 10.1016/j.vaccine.2020.09.004.
[93] J. van den Boogaard *et al.*, "Immunogenicity, duration of protection, effectiveness and safety of rubella containing vaccines: A systematic literature review and meta-analysis," *Vaccine*, vol. 39, no. 6, pp. 889–900, 2021, doi: 10.1016/j.vaccine.2020.12.079.
[94] D. R. Brown *et al.*, "Systematic literature review of cross-protective effect of HPV vaccines based on data from randomized clinical trials and real-world evidence," *Vaccine*, vol. 39, no. 16, pp. 2224–2236, 2021, doi: 10.1016/j.vaccine.2020.11.076.
[95] N. L. Boddington, I. Pearson, H. Whitaker, P. Mangtani, and R. G. Pebody, "Effectiveness of Influenza Vaccination in Preventing Hospitalization Due to Influenza in Children: A Systematic Review and Meta-analysis," *Clinical infectious diseases : an official publication of the Infectious Diseases Society of America*, vol. 73, no. 9, pp. 1722–1732, 2021, doi: 10.1093/cid/ciab270.
[96] J. Xu, P. Doyon-Plourde, M. Tunis, and C. Quach, "Effect of early measles vaccination on long-term protection: A systematic review," *Vaccine*, vol. 39, no. 22, pp. 2929–2937, 2021, doi: 10.1016/j.vaccine.2021.04.012.
[97] D. J. Wall, M. M. Patel, J. R. Chung, B. Lee, and F. S. Dawood, "Antibody Response and Protection After Receipt of Inactivated Influenza Vaccine: A Systematic Review," *Pediatrics*, vol. 147, no. 6, 2021, doi: 10.1542/peds.2020-019901.
[98] B. L. Coleman, R. Sanderson, M. D. M. Haag, and I. McGovern, "Effectiveness of the MF59-adjuvanted trivalent or quadrivalent seasonal influenza vaccine among adults 65 years of age or older, a systematic review and meta-analysis," *Influenza and other respiratory viruses*, vol. 15, no. 6, pp. 813–823, 2021, doi: 10.1111/irv.12871.
[99] Z. Zhang, L. Suo, J. Pan, D. Zhao, and L. Lu, "Two-dose varicella vaccine effectiveness in China: a meta-analysis and evidence quality assessment," *BMC infectious diseases*, vol. 21, no. 1, p. 543, 2021, doi: 10.1186/s12879-021-06217-1.
[100] A. Tsentemeidou *et al.*, "Human Papillomavirus Vaccine to End Oropharyngeal Cancer. A Systematic Review and Meta-Analysis," *Sexually transmitted diseases*, vol. 48, no. 9, pp. 700–707, 2021, doi: 10.1097/OLQ.0000000000001405.
[101] T. Harder *et al.*, "Efficacy and effectiveness of COVID-19 vaccines against SARS-CoV-2 infection: interim results of a living systematic review, 1 January to 14 May 2021," *Euro surveillance : bulletin Europeen sur les maladies transmissibles = European communicable disease bulletin*, vol. 26, no. 28, 2021, doi: 10.2807/1560-7917.ES.2021.26.28.2100563.
[102] K. J. Nielsen, K. K. Jakobsen, J. S. Jensen, C. Gronhoj, and C. Von Buchwald, "The Effect of Prophylactic HPV Vaccines on Oral and Oropharyngeal HPV Infection-A Systematic Review," *Viruses*, vol. 13, no. 7, 2021, doi: 10.3390/v13071339.
[103] T. Harder *et al.*, "Effectiveness of COVID-19 vaccines against SARS-CoV-2 infection with the Delta (B.1.617.2) variant: second interim results of a living systematic review and meta-analysis, 1 January to 25 August 2021," *Euro surveillance : bulletin Europeen sur les maladies transmissibles = European communicable disease bulletin*, vol. 26, no. 41, 2021, doi: 10.2807/1560-7917.ES.2021.26.41.2100920.
[104] N. Sharif, K. J. Alzahrani, S. N. Ahmed, and S. K. Dey, "Efficacy, Immunogenicity and Safety of COVID-19 Vaccines: A Systematic Review and Meta-Analysis," *Frontiers in immunology*, vol. 12, p. 714170, 2021, doi: 10.3389/fimmu.2021.714170.

[105] Q. Liu, C. Qin, M. Liu, and J. Liu, "Effectiveness and safety of SARS-CoV-2 vaccine in real-world studies: a systematic review and meta-analysis," *Infectious diseases of poverty*, vol. 10, no. 1, p. 132, 2021, doi: 10.1186/s40249-021-00915-3.

[106] D. Chen, Y. Li, and Q. Wu, "Effectiveness of varicella vaccine as post-exposure prophylaxis: a meta-analysis," *Human vaccines & immunotherapeutics*, vol. 17, no. 12, pp. 5316–5324, 2021, doi: 10.1080/21645515.2021.2009729.

[107] C. S. Kow and S. S. Hasan, "Real-world effectiveness of BNT162b2 mRNA vaccine: a meta-analysis of large observational studies," *Inflammopharmacology*, vol. 29, no. 4, pp. 1075–1090, 2021, doi: 10.1007/s10787-021-00839-2.

[108] A. Andani, P. van Damme, E. M. Bunge, F. Salgado, R. C. van Hoorn, and B. Hoet, "One or two doses of hepatitis A vaccine in universal vaccination programs in children in 2020: A systematic review," *Vaccine*, vol. 40, no. 2, pp. 196–205, 2022, doi: 10.1016/j.vaccine.2021.01.038.

[109] L. Almasri and B. J. Holtzclaw, "Assessing Vaccine Protection for Older Adults with Diabetes: A Systematic Review," *Western journal of nursing research*, vol. 44, no. 6, pp. 582–597, 2022, doi: 10.1177/01939459211005710.

[110] J. J. L. Sim and C. C. Lim, "Influenza Vaccination in Systemic Lupus Erythematosus: Efficacy, Effectiveness, Safety, Utilization, and Barriers," *The American journal of medicine*, vol. 135, no. 3, pp. 286-296.e9, 2022, doi: 10.1016/j.amjmed.2021.08.038.

[111] W. Fu *et al.*, "Systematic review of the safety, immunogenicity, and effectiveness of COVID-19 vaccines in pregnant and lactating individuals and their infants," *International journal of gynaecology and obstetrics: the official organ of the International Federation of Gynaecology and Obstetrics*, vol. 156, no. 3, pp. 406–417, 2022, doi: 10.1002/ijgo.14008.

[112] W. Wang *et al.*, "Real-world impact and effectiveness assessment of the quadrivalent HPV vaccine: a systematic review of study designs and data sources," *Expert review of vaccines*, vol. 21, no. 2, pp. 227–240, 2022, doi: 10.1080/14760584.2022.2008243.

[113] K.-Y. Sung *et al.*, "SARS-CoV-2 vaccination in patients with inflammatory bowel disease: A systemic review and meta-analysis," *Journal of the Chinese Medical Association : JCMA*, vol. 85, no. 4, pp. 421–430, 2022, doi: 10.1097/JCMA.0000000000000682.

[114] C. S. Kow, D. S. Ramachandram, and S. S. Hasan, "The effectiveness of mRNA-1273 vaccine against COVID-19 caused by Delta variant: A systematic review and meta-analysis," *Journal of medical virology*, vol. 94, no. 5, pp. 2269–2274, 2022, doi: 10.1002/jmv.27568.

[115] P. Izurieta, M. Scherbakov, J. Nieto Guevara, V. Vetter, and L. Soumahoro, "Systematic review of the efficacy, effectiveness and impact of high-valency pneumococcal conjugate vaccines on otitis media," *Human vaccines & immunotherapeutics*, vol. 18, no. 1, p. 2013693, 2022, doi: 10.1080/21645515.2021.2013693.

[116] J. Puig-Barbera, S. Tamames-Gomez, P. Plans-Rubio, and J. M. Eiros-Bouza, "Relative Effectiveness of Cell-Cultured versus Egg-Based Seasonal Influenza Vaccines in Preventing Influenza-Related Outcomes in Subjects 18 Years Old or Older: A Systematic Review and Meta-Analysis," *International journal of environmental research and public health*, vol. 19, no. 2, 2022, doi: 10.3390/ijerph19020818.

[117] C. S. C. Kow, D. S. Ramachandram, and S. S. Hasan, "The effectiveness of BNT162b2 mRNA vaccine against COVID-19 caused by Delta variant of SARS-CoV-2: a systematic review and meta-analysis," *Inflammopharmacology*, vol. 30, no. 1, pp. 149–157, 2022, doi: 10.1007/s10787-021-00915-7.

[118] Y. Z. Huang and C. C. Kuan, "Vaccination to reduce severe COVID-19 and mortality in COVID-19 patients: a systematic review and meta-analysis," *European review for medical and pharmacological sciences*, vol. 26, no. 5, pp. 1770–1776, 2022, doi: 10.26355/eurrev_202203_28248.

[119] A. Bhurwal *et al.*, "Effectiveness and safety of SARS-CoV-2 vaccine in Inflammatory Bowel Disease patients: a systematic review, meta-analysis and meta-regression," *Alimentary pharmacology & therapeutics*, vol. 55, no. 10, pp. 1244–1264, 2022, doi: 10.1111/apt.16913.

[120] C. Gertosio, A. Licari, A. De Silvestri, C. Rebuffi, E. Chiappini, and G. L. Marseglia, "Efficacy, immunogenicity, and safety of available vaccines in children on biologics: A systematic review and meta-analysis," *Vaccine*, vol. 40, no. 19, pp. 2679–2695, 2022, doi: 10.1016/j.vaccine.2022.03.041.

[121] C. Zheng, W. Shao, X. Chen, B. Zhang, G. Wang, and W. Zhang, "Real-world effectiveness of COVID-19 vaccines: a literature review and meta-analysis," *International journal of infectious diseases : IJID : official publication of the International Society for Infectious Diseases*, vol. 114, pp. 252–260, 2022, doi: 10.1016/j.ijid.2021.11.009.

[122] S. Galmiche *et al.*, "Immunological and clinical efficacy of COVID-19 vaccines in immunocompromised populations: a systematic review," *Clinical microbiology and infection : the official publication of the European Society of Clinical Microbiology and Infectious Diseases*, vol. 28, no. 2, pp. 163–177, 2022, doi: 10.1016/j.cmi.2021.09.036.

[123] A. R. Marra *et al.*, "Short-term effectiveness of COVID-19 vaccines in immunocompromised patients: A systematic literature review and meta-analysis," *The Journal of infection*, vol. 84, no. 3, pp. 297–310, 2022, doi: 10.1016/j.jinf.2021.12.035.

[124] B. C. Gartner *et al.*, "Importance and value of adjuvanted influenza vaccine in the care of older adults from a European perspective - A systematic review of recently published literature on real-world data," *Vaccine*, vol. 40, no. 22, pp. 2999–3008, 2022, doi: 10.1016/j.vaccine.2022.04.019.

[125] P. Ssentongo *et al.*, "SARS-CoV-2 vaccine effectiveness against infection, symptomatic and severe COVID-19: a systematic review and meta-analysis," *BMC infectious diseases*, vol. 22, no. 1, p. 439, 2022, doi: 10.1186/s12879-022-07418-y.

[126] S. Prasad *et al.*, "Systematic review and meta-analysis of the effectiveness and perinatal outcomes of COVID-19 vaccination in pregnancy," *Nature communications*, vol. 13, no. 1, p. 2414, 2022, doi: 10.1038/s41467-022-30052-w.

[127] C. Gupta, A. Sachdeva, J. Khamar, C. Bu, J. Bartoszko, and M. Loeb, "Effectiveness of the influenza vaccine at reducing adverse events in patients with heart failure: A systematic review and meta-analysis," *Vaccine*, vol. 40, no. 25, pp. 3433–3443, 2022, doi: 10.1016/j.vaccine.2022.04.039.

[128] B. Zeng, L. Gao, Q. Zhou, K. Yu, and F. Sun, "Effectiveness of COVID-19 vaccines against SARS-CoV-2 variants of concern: a systematic review and meta-analysis," *BMC medicine*, vol. 20, no. 1, p. 200, 2022, doi: 10.1186/s12916-022-02397-y.

[129] K. Tsiakos, K. G. Kyriakoulis, A. Kollias, I. G. Kyriakoulis, G. Poulakou, and K. Syrigos, "Influenza Vaccination in Cancer Patients Treated With Immune Checkpoint Inhibitors: A Systematic Review and Meta-analysis," *Journal of immunotherapy (Hagerstown, Md. : 1997)*, vol. 45, no. 6, pp. 291–298, 2022, doi: 10.1097/CJI.0000000000000424.

[130] V. Piechotta *et al.*, "Effectiveness, immunogenicity, and safety of COVID-19 vaccines for individuals with hematological malignancies: a systematic review," *Blood cancer journal*, vol. 12, no. 5, p. 86, 2022, doi: 10.1038/s41408-022-00684-8.

[131] L. P. Marra, A. L. Sartori, M. S. Martinez-Silveira, C. M. Toscano, and A. L. Andrade, "Effectiveness of Pneumococcal Vaccines on Otitis Media in Children: A Systematic Review," *Value in health : the journal of the International Society for Pharmacoeconomics and Outcomes Research*, vol. 25, no. 6, pp. 1042–1056, 2022, doi: 10.1016/j.jval.2021.12.012.

[132] H. R. Baradaran *et al.*, "The effect of COVID-19 mRNA vaccines against postvaccination laboratory-confirmed SARS-CoV-2 infection, symptomatic COVID-19 infection, hospitalization, and mortality rate: a systematic review and meta-analysis," *Expert review of vaccines*, vol. 21, no. 10, pp. 1455–1464, 2022, doi: 10.1080/14760584.2022.2102001.

[133] A. Domnich and C. de Waure, "Comparative effectiveness of adjuvanted versus high-dose seasonal influenza vaccines for older adults: a systematic review and meta-analysis," *International journal of infectious diseases : IJID : official publication of the International Society for Infectious Diseases*, vol. 122, pp. 855–863, 2022, doi: 10.1016/j.ijid.2022.07.048.

[134] Y. Zou, D. Huang, Q. Jiang, Y. Guo, and C. Chen, "The Vaccine Efficacy Against the SARS-CoV-2 Omicron: A Systemic Review and Meta-Analysis," *Frontiers in public health*, vol. 10, p. 940956, 2022, doi: 10.3389/fpubh.2022.940956.

[135] K. S. Kechagias *et al.*, "Role of human papillomavirus (HPV) vaccination on HPV infection and recurrence of HPV related disease after local surgical treatment: systematic review and meta-analysis," *BMJ (Clinical research ed.)*, vol. 378, p. e070135, 2022, doi: 10.1136/bmj-2022-070135.

[136] W. Shao *et al.*, "Effectiveness of COVID-19 vaccines against SARS-CoV-2 variants of concern in real-world: a literature review and meta-analysis," *Emerging microbes & infections*, vol. 11, no. 1, pp. 2383–2392, 2022, doi: 10.1080/22221751.2022.2122582.

[137] W. Kulper-Schiek *et al.*, "Facing the Omicron variant-how well do vaccines protect against mild and severe COVID-19? Third interim analysis of a living systematic review," *Frontiers in immunology*, vol. 13, p. 940562, 2022, doi: 10.3389/fimmu.2022.940562.

[138] Y. Xia, X. Zhang, L. Zhang, and C. Fu, "Efficacy, effectiveness, and safety of herpes zoster vaccine in the immunocompetent and immunocompromised subjects: A systematic review and network meta-analysis," *Frontiers in immunology*, vol. 13, p. 978203, 2022, doi: 10.3389/fimmu.2022.978203.

[139] M. Petras *et al.*, "Risk factors affecting COVID-19 vaccine effectiveness identified from 290 cross-country observational studies until February 2022: a meta-analysis and meta-regression," *BMC medicine*, vol. 20, no. 1, p. 461, 2022, doi: 10.1186/s12916-022-02663-z.

[140] L. E. Markowitz *et al.*, "Human papillomavirus vaccine effectiveness by number of doses: Updated systematic review of data from national immunization programs," *Vaccine*, vol. 40, no. 37, pp. 5413–5432, 2022, doi: 10.1016/j.vaccine.2022.06.065.

[141] J. F. Mbinta, B. P. Nguyen, P. M. A. Awuni, J. Paynter, and C. R. Simpson, "Post-licensure zoster vaccine effectiveness against herpes zoster and postherpetic neuralgia in older adults: a systematic review and meta-analysis," *The Lancet. Healthy longevity*, vol. 3, no. 4, pp. e263–e275, 2022, doi: 10.1016/S2666-7568(22)00039-3.

[142] W. Y. Au and P. P.-H. Cheung, "Effectiveness of heterologous and homologous covid-19 vaccine regimens: living systematic review with network meta-analysis," *BMJ (Clinical research ed.)*, vol. 377, p. e069989, 2022, doi: 10.1136/bmj-2022-069989.

[143] J. Deng, Y. Ma, Q. Liu, M. Du, M. Liu, and J. Liu, "Comparison of the Effectiveness and Safety of Heterologous Booster Doses with Homologous Booster Doses for SARS-CoV-2 Vaccines: A Systematic Review and Meta-Analysis," *International journal of environmental research and public health*, vol. 19, no. 17, 2022, doi: 10.3390/ijerph191710752.

[144] P. Gao, J. Liu, and M. Liu, "Effect of COVID-19 Vaccines on Reducing the Risk of Long COVID in the Real World: A Systematic Review and Meta-Analysis," *International journal of environmental research and public health*, vol. 19, no. 19, 2022, doi: 10.3390/ijerph191912422.

[145] K. Rahmani *et al.*, "The effectiveness of COVID-19 vaccines in reducing the incidence, hospitalization, and mortality from COVID-19: A systematic review and meta-analysis," *Frontiers in public health*, vol. 10, p. 873596, 2022, doi: 10.3389/fpubh.2022.873596.

[146] L. Martinez *et al.*, "Infant BCG vaccination and risk of pulmonary and extrapulmonary tuberculosis throughout the life course: a systematic review and individual participant data meta-analysis," *The Lancet. Global health*, vol. 10, no. 9, pp. e1307–e1316, 2022, doi: 10.1016/S2214-109X(22)00283-2.
[147] J. D. Berild *et al.*, "A Systematic Review of Studies Published between 2016 and 2019 on the Effectiveness and Efficacy of Pneumococcal Vaccination on Pneumonia and Invasive Pneumococcal Disease in an Elderly Population," *Pathogens (Basel, Switzerland)*, vol. 9, no. 4, 2020, doi: 10.3390/pathogens9040259.
[148] A. Bechini *et al.*, "Impact of Influenza Vaccination on All-Cause Mortality and Hospitalization for Pneumonia in Adults and the Elderly with Diabetes: A Meta-Analysis of Observational Studies," *Vaccines*, vol. 8, no. 2, 2020, doi: 10.3390/vaccines8020263.
[149] N. Murunga, G. P Otieno, M. Maia, and C. N Agoti, "Effectiveness of Rotarix R vaccine in Africa in the first decade of progressive introduction, 2009-2019: systematic review and meta-analysis," *Wellcome open research*, vol. 5, p. 187, 2020, doi: 10.12688/wellcomeopenres.16174.2.
[150] X. Yang *et al.*, "Influenza Vaccine Effectiveness in Mainland China: A Systematic Review and Meta-Analysis," *Vaccines*, vol. 9, no. 2, 2021, doi: 10.3390/vaccines9020079.
[151] G. N. Okoli *et al.*, "Decline in Seasonal Influenza Vaccine Effectiveness With Vaccination Program Maturation: A Systematic Review and Meta-analysis," *Open forum infectious diseases*, vol. 8, no. 3, p. ofab069, 2021, doi: 10.1093/ofid/ofab069.
[152] C. O. Iheanacho, U. I. H. Eze, and E. A. Adida, "A systematic review of effectiveness of BNT162b2 mRNA and ChAdOx1 adenoviral vector COVID-19 vaccines in the general population," *Bulletin of the National Research Centre*, vol. 45, no. 1, p. 150, 2021, doi: 10.1186/s42269-021-00607-w.
[153] Y.-J. Fan, K.-H. Chan, and I. F.-N. Hung, "Safety and Efficacy of COVID-19 Vaccines: A Systematic Review and Meta-Analysis of Different Vaccines at Phase 3," *Vaccines*, vol. 9, no. 9, 2021, doi: 10.3390/vaccines9090989.
[154] G. Perego *et al.*, "Safety and Efficacy of Spray Intranasal Live Attenuated Influenza Vaccine: Systematic Review and Meta-Analysis," *Vaccines*, vol. 9, no. 9, 2021, doi: 10.3390/vaccines9090998.
[155] T. Li *et al.*, "A Systematic Review and Meta-Analysis of Seasonal Influenza Vaccination of Health Workers," *Vaccines*, vol. 9, no. 10, 2021, doi: 10.3390/vaccines9101104.
[156] K. Hayawi, S. Shahriar, M. A. Serhani, H. Alashwal, and M. M. Masud, "Vaccine versus Variants (3Vs): Are the COVID-19 Vaccines Effective against the Variants? A Systematic Review," *Vaccines*, vol. 9, no. 11, 2021, doi: 10.3390/vaccines9111305.
[157] C.-J. Cheng *et al.*, "Effectiveness of the WHO-Authorized COVID-19 Vaccines: A Rapid Review of Global Reports till 30 June 2021," *Vaccines*, vol. 9, no. 12, 2021, doi: 10.3390/vaccines9121489.
[158] A. Pormohammad *et al.*, "Effectiveness of COVID-19 Vaccines against Delta (B.1.617.2) Variant: A Systematic Review and Meta-Analysis of Clinical Studies," *Vaccines*, vol. 10, no. 1, 2021, doi: 10.3390/vaccines10010023.
[159] A. R. Marra *et al.*, "The short-term effectiveness of coronavirus disease 2019 (COVID-19) vaccines among healthcare workers: a systematic literature review and meta-analysis," *Antimicrobial stewardship & healthcare epidemiology : ASHE*, vol. 1, no. 1, p. e33, 2021, doi: 10.1017/ash.2021.195.
[160] A. Meggiolaro, M. Sane Schepisi, G. F. Nikolaidis, D. Mipatrini, A. Siddu, and G. Rezza, "Effectiveness of Vaccination against SARS-CoV-2 Infection in the Pre-Delta Era: A Systematic Review and Meta-Analysis," *Vaccines*, vol. 10, no. 2, 2022, doi: 10.3390/vaccines10020157.
[161] Y. Ma, J. Deng, Q. Liu, M. Du, M. Liu, and J. Liu, "Effectiveness and Safety of COVID-19 Vaccine among Pregnant Women in Real-World Studies: A Systematic Review and Meta-Analysis," *Vaccines*, vol. 10, no. 2, 2022, doi: 10.3390/vaccines10020246.
[162] R. A. Mahumud, M. A. Ali, S. Kundu, M. A. Rahman, J. K. Kamara, and A. M. N. Renzaho, "Effectiveness of COVID-19 Vaccines against Delta Variant (B.1.617.2): A Meta-Analysis," *Vaccines*, vol. 10, no. 2, 2022, doi: 10.3390/vaccines10020277.
[163] R. M. Ghazy *et al.*, "Efficacy and Effectiveness of SARS-CoV-2 Vaccines: A Systematic Review and Meta-Analysis," *Vaccines*, vol. 10, no. 3, 2022, doi: 10.3390/vaccines10030350.
[164] S. Chang *et al.*, "Effectiveness of BNT162b2 and mRNA-1273 Vaccines against COVID-19 Infection: A Meta-Analysis of Test-Negative Design Studies," *Vaccines*, vol. 10, no. 3, 2022, doi: 10.3390/vaccines10030469.
[165] K. Wang *et al.*, "Real-Word Effectiveness of Global COVID-19 Vaccines Against SARS-CoV-2 Variants: A Systematic Review and Meta-Analysis," *Frontiers in medicine*, vol. 9, p. 820544, 2022, doi: 10.3389/fmed.2022.820544.
[166] S. Cai *et al.*, "Effectiveness and Safety of Therapeutic Vaccines for Precancerous Cervical Lesions: A Systematic Review and Meta-Analysis," *Frontiers in oncology*, vol. 12, p. 918331, 2022, doi: 10.3389/fonc.2022.918331.
[167] M. H. Jansen *et al.*, "Efficacy, Immunogenicity and Safety of Vaccination in Pediatric Patients With Autoimmune Inflammatory Rheumatic Diseases (pedAIIRD): A Systematic Literature Review for the 2021 Update of the EULAR/PRES Recommendations," *Frontiers in pediatrics*, vol. 10, p. 910026, 2022, doi: 10.3389/fped.2022.910026.

[168] Y. Zhu, S. Liu, and D. Zhang, “Effectiveness of COVID-19 Vaccine Booster Shot Compared with Non-Booster: A Meta-Analysis,” *Vaccines*, vol. 10, no. 9, 2022, doi: 10.3390/vaccines10091396.

[169] P. Foucambert *et al.*, “Efficacy of Dengue Vaccines in the Prevention of Severe Dengue in Children: A Systematic Review,” *Cureus*, vol. 14, no. 9, p. e28916, 2022, doi: 10.7759/cureus.28916.

[170] A. R. Marra *et al.*, “The long-term effectiveness of coronavirus disease 2019 (COVID-19) vaccines: A systematic literature review and meta-analysis,” *Antimicrobial stewardship & healthcare epidemiology : ASHE*, vol. 2, no. 1, p. e22, 2022, doi: 10.1017/ash.2021.261.

[171] M. Liu *et al.*, “Influenza vaccination is associated with a decreased risk of atrial fibrillation: A systematic review and meta-analysis,” *Frontiers in cardiovascular medicine*, vol. 9, p. 970533, 2022, doi: 10.3389/fcvm.2022.970533.

[172] J. M. Sabu, I. Zahid, N. Jacob, F. O. Alele, and B. S. Malau-Aduli, “Effectiveness of the BNT162b2 (Pfizer-BioNTech) Vaccine in Children and Adolescents: A Systematic Review and Meta-Analysis,” *Vaccines*, vol. 10, no. 11, 2022, doi: 10.3390/vaccines10111880.

[173] M. Wallace *et al.*, “Effectiveness of Pfizer-BioNTech COVID-19 vaccine as evidence for policy action: A rapid systematic review and meta-analysis of non-randomized studies,” *PloS one*, vol. 17, no. 12, p. e0278624, 2022, doi: 10.1371/journal.pone.0278624.

[174] N. Angkasekwinai *et al.*, “Binding and neutralizing antibody levels and vaccine efficacy/effectiveness compared between heterologous and homologous primary series COVID-19 vaccination: A systematic review and meta-analysis,” *Asian Pacific journal of allergy and immunology*, vol. 40, no. 4, pp. 321–336, 2022, doi: 10.12932/AP-121122-1501.

[175] J. I. Ruiz, M. A. Lopez-Olivo, Y. Geng, and M. E. Suarez-Almazor, “COVID-19 vaccination in patients with cancer receiving immune checkpoint inhibitors: a systematic review and meta-analysis,” *Journal for immunotherapy of cancer*, vol. 11, no. 2, 2023, doi: 10.1136/jitc-2022-006246.

[176] S. Wu *et al.*, “The dose- and time-dependent effectiveness and safety associated with COVID-19 vaccination during pregnancy: a systematic review and meta-analysis,” *International journal of infectious diseases : IJID : official publication of the International Society for Infectious Diseases*, vol. 128, pp. 335–346, 2023, doi: 10.1016/j.ijid.2023.01.018.

[177] P. Paul *et al.*, “Effectiveness of the pre-Omicron COVID-19 vaccines against Omicron in reducing infection, hospitalization, severity, and mortality compared to Delta and other variants: A systematic review,” *Human vaccines & immunotherapeutics*, vol. 19, no. 1, p. 2167410, 2023, doi: 10.1080/21645515.2023.2167410.

[178] E. Jones-Gray, E. J. Robinson, A. J. Kucharski, A. Fox, and S. G. Sullivan, “Does repeated influenza vaccination attenuate effectiveness? A systematic review and meta-analysis,” *The Lancet. Respiratory medicine*, vol. 11, no. 1, pp. 27–44, 2023, doi: 10.1016/S2213-2600(22)00266-1.

[179] I. Hameed *et al.*, “Is it safe and effective to administer COVID-19 vaccines during pregnancy? A systematic review and meta-analysis,” *American journal of infection control*, vol. 51, no. 5, pp. 582–593, 2023, doi: 10.1016/j.ajic.2022.08.014.

[180] S. Xu, J. Li, H. Wang, F. Wang, Z. Yin, and Z. Wang, “Real-world effectiveness and factors associated with effectiveness of inactivated SARS-CoV-2 vaccines: a systematic review and meta-regression analysis,” *BMC medicine*, vol. 21, no. 1, p. 160, 2023, doi: 10.1186/s12916-023-02861-3.

[181] S. Y. S. Tan, A. M. Yee, J. J. L. Sim, and C. C. Lim, “COVID-19 vaccination in systemic lupus erythematosus: a systematic review of its effectiveness, immunogenicity, flares and acceptance,” *Rheumatology (Oxford, England)*, vol. 62, no. 5, pp. 1757–1772, 2023, doi: 10.1093/rheumatology/keac604.

[182] F. Menegale *et al.*, “Evaluation of Waning of SARS-CoV-2 Vaccine-Induced Immunity: A Systematic Review and Meta-analysis,” *JAMA network open*, vol. 6, no. 5, p. e2310650, 2023, doi: 10.1001/jamanetworkopen.2023.10650.

[183] L. Comber *et al.*, “Systematic review of the efficacy, effectiveness and safety of high-dose seasonal influenza vaccines for the prevention of laboratory-confirmed influenza in individuals >=18 years of age,” *Reviews in medical virology*, vol. 33, no. 3, p. e2330, 2023, doi: 10.1002/rmv.2330.

[184] K. Jordan *et al.*, “Systematic review of the efficacy, effectiveness and safety of cell-based seasonal influenza vaccines for the prevention of laboratory-confirmed influenza in individuals >=18 years of age,” *Reviews in medical virology*, vol. 33, no. 3, p. e2332, 2023, doi: 10.1002/rmv.2332.

[185] E. O Murchu *et al.*, “Systematic review of the efficacy, effectiveness and safety of MF59 R adjuvanted seasonal influenza vaccines for the prevention of laboratory-confirmed influenza in individuals >=18 years of age,” *Reviews in medical virology*, vol. 33, no. 3, p. e2329, 2023, doi: 10.1002/rmv.2329.

[186] N. Wu *et al.*, “Long-term effectiveness of COVID-19 vaccines against infections, hospitalisations, and mortality in adults: findings from a rapid living systematic evidence synthesis and meta-analysis up to December, 2022,” *The Lancet. Respiratory medicine*, vol. 11, no. 5, pp. 439–452, 2023, doi: 10.1016/S2213-2600(23)00015-2.

[187] A. R. Marra *et al.*, “The effectiveness of coronavirus disease 2019 (COVID-19) vaccine in the prevention of post-COVID-19 conditions: A systematic literature review and meta-analysis,” *Antimicrobial stewardship & healthcare epidemiology : ASHE*, vol. 2, no. 1, p. e192, 2022, doi: 10.1017/ash.2022.336.

[188] M. A. Lopez-Olivo *et al.*, “Safety and Efficacy of Influenza Vaccination in Patients Receiving Immune Checkpoint Inhibitors. Systematic Review with Meta-Analysis,” *Vaccines*, vol. 10, no. 8, 2022, doi: 10.3390/vaccines10081195.

[189] J. M. van den Berg *et al.*, "Effectiveness of COVID-19 Vaccines in Adults with Diabetes Mellitus: A Systematic Review," *Vaccines*, vol. 11, no. 1, 2022, doi: 10.3390/vaccines11010024.
[190] M. G. Sikjaer, A. A. Pedersen, M. S. Wik, S. S. Stensholt, O. Hilberg, and A. Lokke, "Vaccine effectiveness of the pneumococcal polysaccharide and conjugated vaccines in elderly and high-risk populations in preventing invasive pneumococcal disease: a systematic search and meta-analysis," *European clinical respiratory journal*, vol. 10, no. 1, p. 2168354, 2023, doi: 10.1080/20018525.2023.2168354.
[191] H. Mohammed *et al.*, "A Systematic Review and Meta-Analysis on the Real-World Effectiveness of COVID-19 Vaccines against Infection, Symptomatic and Severe COVID-19 Disease Caused by the Omicron Variant (B.1.1.529)," *Vaccines*, vol. 11, no. 2, 2023, doi: 10.3390/vaccines11020224.
[192] A. Beran *et al.*, "Real-world effectiveness of COVID-19 vaccination in liver cirrhosis: a systematic review with meta-analysis of 51,834 patients," *Proceedings (Baylor University. Medical Center)*, vol. 36, no. 2, pp. 151–156, 2023, doi: 10.1080/08998280.2023.2165344.
[193] N. R. Pratama *et al.*, "Effectiveness of COVID-19 Vaccines against SARS-CoV-2 Omicron Variant (B.1.1.529): A Systematic Review with Meta-Analysis and Meta-Regression," *Vaccines*, vol. 10, no. 12, 2022, doi: 10.3390/vaccines10122180.
[194] P. Gao, S. Cai, Q. Liu, M. Du, J. Liu, and M. Liu, "Effectiveness and Safety of SARS-CoV-2 Vaccines among Children and Adolescents: A Systematic Review and Meta-Analysis," *Vaccines*, vol. 10, no. 3, 2022, doi: 10.3390/vaccines10030421.
[195] O. Byambasuren, P. Stehlik, J. Clark, K. Alcorn, and P. Glasziou, "Effect of covid-19 vaccination on long covid: systematic review," *BMJ medicine*, vol. 2, no. 1, p. e000385, 2023, doi: 10.1136/bmjmed-2022-000385.
[196] P. Macilwraith, E. Malsem, and S. Dushyanthen, "The effectiveness of HPV vaccination on the incidence of oropharyngeal cancers in men: a review," *Infectious agents and cancer*, vol. 18, no. 1, p. 24, 2023, doi: 10.1186/s13027-022-00479-3.
[197] I. Dicembrini *et al.*, "Influenza: Diabetes as a risk factor for severe related-outcomes and the effectiveness of vaccination in diabetic population. A meta-analysis of observational studies," *Nutrition, metabolism, and cardiovascular diseases : NMCD*, vol. 33, no. 6, pp. 1099–1110, 2023, doi: 10.1016/j.numecd.2023.03.016.
[198] P. Gao, L.-Y. Kang, J. Liu, and M. Liu, "Immunogenicity, effectiveness, and safety of COVID-19 vaccines among children and adolescents aged 2-18 years: an updated systematic review and meta-analysis," *World journal of pediatrics : WJP*, 2023, doi: 10.1007/s12519-022-00680-9.
[199] C.-G. Kontovazainitis, G. N. Katsaras, D. Gialamprinou, and G. Mitsiakos, "Covid-19 vaccination and pregnancy: a systematic review of maternal and neonatal outcomes," *Journal of perinatal medicine*, 2023, doi: 10.1515/jpm-2022-0463.
[200] K. Guo, P. Ni, S. Chang, Y. Jin, G. Duan, and R. Zhang, "Effectiveness of mRNA vaccine against Omicron-related infections in the real world: A systematic review and meta-analysis," *American journal of infection control*, 2023, doi: 10.1016/j.ajic.2023.02.005.
[201] V. Piechotta *et al.*, "Safety and effectiveness of vaccines against COVID-19 in children aged 5-11 years: a systematic review and meta-analysis," *The Lancet. Child & adolescent health*, 2023, doi: 10.1016/S2352-4642(23)00078-0.
[202] P. Dmc. Katoto *et al.*, "Effectiveness of COVID-19 Pfizer-BioNTech (BNT162b2) mRNA vaccination in adolescents aged 12–17 years: A systematic review and meta-analysis," *Human Vaccines & Immunotherapeutics*, vol. 19, no. 1, p. 2214495, Jan. 2023, doi: 10.1080/21645515.2023.2214495.
[203] E. J. D. A. Luna, V. L. Gattás, and S. R. D. S. L. D. C. Campos, "Efetividade da estratégia brasileira de vacinação contra influenza: uma revisão sistemática," *Epidemiol. Serv. Saúde*, vol. 23, no. 3, pp. 559–576, Sep. 2014, doi: 10.5123/S1679-49742014000300020.
[204] Wells GA *et al.*, "The Newcastle-Ottawa Scale (NOS) for assessing the quality of nonrandomised studies in meta-analyses," Ottawa Hospital Research Institute, Ottawa (ON), 2011.
[205] J. A. Sterne *et al.*, "ROBINS-I: a tool for assessing risk of bias in non-randomised studies of interventions," *BMJ*, p. i4919, Oct. 2016, doi: 10.1136/bmj.i4919.
[206] S. H. Downs and N. Black, "The feasibility of creating a checklist for the assessment of the methodological quality both of randomised and non-randomised studies of health care interventions.," *J Epidemiol Community Health*, vol. 52, no. 6, pp. 377–384, Jun. 1998, doi: 10.1136/jech.52.6.377.
[207] Joanna Briggs Institute, "Critical appraisal tools," Adelaide (AU), 2017. [Online]. Available: https://jbi.global/critical-appraisal-tools
[208] R. Sirriyeh, R. Lawton, P. Gardner, and G. Armitage, "Reviewing studies with diverse designs: the development and evaluation of a new tool," *Journal of Evaluation in Clinical Practice*, vol. 18, no. 4, pp. 746–752, 2012, doi: 10.1111/j.1365-2753.2011.01662.x.
[209] "Critical Appraisal Skills Programme (CASP)," Oxford. UK. [Online]. Available: https://casp-uk.net/casp-tools-checklists/
[210] R. P. Harris *et al.*, "Current methods of the U.S. Preventive Services Task Force," *American Journal of Preventive Medicine*, vol. 20, no. 3, pp. 21–35, Apr. 2001, doi: 10.1016/S0749-3797(01)00261-6.
[211] J. P. T. Higgins *et al.*, "The Cochrane Collaboration's tool for assessing risk of bias in randomised trials," *BMJ*, vol. 343, no. oct18 2, pp. d5928–d5928, Oct. 2011, doi: 10.1136/bmj.d5928.

[212] Bacon SL, Wu N, Joyal-Desmarais K, Vieira AM, Sanuade C, Jagwani M, Paquet L, Ribeiro PAB, Yip D, Stojanovic J. COVID19 living evidence synthesis #10 (version 10.16): What is the long-term effectiveness of available COVID-19 vaccines for adults, including for variants of concern and over time frames beyond 112 days in those with a primary series and beyond 84 days in those with a primary series and additional doses? The Montreal Behavioural Medicine Centre, META group, 29 March 2023

[213] National Collaborating Centre for Methods and Tools. (2021, October 15). *Rapid Review Update 1: What is the effectiveness, immunogenicity, and safety of COVID-19 vaccines in persons who have had a prior, confirmed COVID-19 infection?* https://www.nccmt.ca/covid-19/covid-19-rapid-evidence-service/36

[214] Egunsola O, Mastikhina L, Dowsett LE, Clement FM on behalf of the University of Calgary Health Technology Assessment Unit. Transmissibility of COVID-19 among Vaccinated Individuals: Targeted Literature Search. March 2, 2021.

[215] Public Health Agency of Canada, Canada National Advisory Committee on Immunization, 'Recommendation on Repeated Seasonal Influenza Vaccination,' Public Health Agency of Canada = Agence de la santé publique du Canada, 2023. [Online]. Available: http://central.bac-lac.gc.ca/.redirect?app=damspub&id=e1b43afe-6bc0-4738-9b10-19c6d1c46ffe.".

[216] Public Health Agency of Canada Infectious Disease Prevention and Control Branch, 'Updated Recommendations on the Use of Herpes Zoster Vaccines,' Public Health Agency of Canada, 2018. [Online]. Available: http://epe.lac-bac.gc.ca/100/201/301/weekly_acquisitions_list-ef/2018/18-44/publications.gc.ca/collections/collection_2018/aspc-phac/HP40-212-2018-eng.pdf.

[217] European Centre for Disease Prevention and Control., *Systematic review of the efficacy, effectiveness and safety of newer and enhanced seasonal influenza vaccines for the prevention of laboratory-confirmed influenza in individuals aged 18 years and over.* LU: Publications Office, 2020. Accessed: Jan. 15, 2025. [Online]. Available: https://data.europa.eu/doi/10.2900/751620.
