# Supplementary material for: Risk-of-bias assessment of vaccine effectiveness studies: a scoping review of systematic reviews
Source: Epidemiol Infect. 2026 Jun 19;154:e95. doi: 10.1017/S0950268826101794 (PMC13366358; doi:10.1017/S0950268826101794)
Supplement: Davoodi et al. supplementary material [file S0950268826101794sup001.zip › S0950268826101794sup007.pdf]

APPENDIX G: EXCLUDED STUDIES

Exclusion reason: Wrong outcome (n=46)

[1] Ahmad N, Mohammed Nawi A, Jamhari MN, Nurumal SR, Mansor J, Ahmad Zamzuri M 'Ammar I, et al. Post-Exposure Prophylactic Vaccination against Rabies: A Systematic Review. Ijph 2022. https://doi.org/10.18502/ijph.v51i5.9412.

[2] Al-Ali D, Elshafeey A, Mushannen M, Kawas H, Shafiq A, Mhaimeed N, et al. Cardiovascular and haematological events post COVID-19 vaccination: A systematic review. J Cellular Molecular Medi 2022;26:636–53. https://doi.org/10.1111/jcmm.17137.

[3] Alizadeh, L.; Ghahremani, F.; Khalili, S. M.; Havaei, M.; Hajirafiei, E.; Riazi, H.; Keshavarz, Z. The association of human papillomavirus vaccine with premature ovarian failure: A systematic review. IJOGI 2023;25. https://doi.org/10.22038/ijogi.2023.21798.

[4] Angeli F, Reboldi G, Trapasso M, Santilli G, Zappa M, Verdecchia P. Blood Pressure Increase following COVID-19 Vaccination: A Systematic Overview and Meta-Analysis. JCDD 2022;9:150. https://doi.org/10.3390/jcdd9050150.

[5] Barte H, Horvath TH, Rutherford GW. Yellow fever vaccine for patients with HIV infection. Cochrane Database of Systematic Reviews 2014;2014. https://doi.org/10.1002/14651858.CD010929.pub2.

[6] Bobrovitz N, Ware H, Ma X, Li Z, Hosseini R, Cao C, et al. Protective effectiveness of previous SARS-CoV-2 infection and hybrid immunity against the omicron variant and severe disease: a systematic review and meta-regression. The Lancet Infectious Diseases 2023;23:556–67. https://doi.org/10.1016/S1473-3099(22)00801-5.

[7] Burnett E, Parashar UD, Tate JE. Real-world effectiveness of rotavirus vaccines, 2006–19: a literature review and meta-analysis. The Lancet Global Health 2020;8:e1195–202. https://doi.org/10.1016/S2214-109X(20)30262-X.

[8] Choi M, Yu S-Y, Cheong C, Choe YJ, Choi S-H. Efficacy and Safety of COVID-19 Vaccines in Children Aged 5 to 11 Years: A Systematic Review. Pediatr Infect Vaccine 2022;29:28. https://doi.org/10.14776/piv.2022.29.e4.

[9] Cunninghame J, Wen S, Dufficy M, Ullman A, Takashima M, Cann M, et al. Immunogenicity and safety of vaccination in children with paediatric rheumatic diseases: a scoping review. Therapeutic Advances in Vaccines and Immunotherapy 2023;11:25151355231167116. https://doi.org/10.1177/25151355231167116.

[10] Etemadifar M, Nouri H, Pitzalis M, Idda ML, Salari M, Baratian M, et al. Multiple sclerosis disease-modifying therapies and COVID-19 vaccines: a practical review and meta-analysis. J Neurol Neurosurg Psychiatry 2022;93:986–94. https://doi.org/10.1136/jnnp-2022-329123.

[11] Ferahta N, Achek I, Dubourg J, Lang P-O. Les vaccins contre le zona : efficacité, sécurité, et rapport coût/bénéfices. La Presse Médicale 2016;45:162–76. https://doi.org/10.1016/j.lpm.2015.10.015.

[12] Iheanacho CO, Eze UIH. Immunogenicity and clinical features relating to BNT162b2 messenger RNA COVID-19 vaccine, Ad26.COV2.S and ChAdOx1 adenoviral vector COVID-19 vaccines: a

systematic review of non-interventional studies. Futur J Pharm Sci 2022;8:20. https://doi.org/10.1186/s43094-022-00409-5.

[13] Jena A, James D, Singh AK, Dutta U, Sebastian S, Sharma V. Effectiveness and Durability of COVID-19 Vaccination in 9447 Patients With IBD: A Systematic Review and Meta-Analysis. Clinical Gastroenterology and Hepatology 2022;20:1456-1479.e18. https://doi.org/10.1016/j.cgh.2022.02.030.

[14] Jiang H, Shi Y, Zhang X, Pan L, Xie Y, Jiang C, et al. Human papillomavirus vaccination and the risk of autoimmune disorders: A systematic review and meta-analysis. Vaccine 2019;37:3031–9. https://doi.org/10.1016/j.vaccine.2019.04.049.

[15] Katoto PDMC, Brand AS, Byamungu LN, Tamuzi JL, Mahwire TC, Kitenge MK, et al. Safety of COVID-19 Pfizer-BioNtech (BNT162b2) mRNA vaccination in adolescents aged 12–17 years: A systematic review and meta-analysis. Human Vaccines & Immunotherapeutics 2022;18:2144039. https://doi.org/10.1080/21645515.2022.2144039.

[16] Katoto PD, Kakubu MA, Tamuzi JL, Brand AS, Ayuk A, Byamungu LN, et al. Immunogenicity and reactogenicity of COVID-19 Pfizer-BioNTech (Bnt162b2) mRNA vaccination in immunocompromised adolescents and young adults: a systematic review and meta-analyses. Expert Review of Vaccines 2023;22:378–92. https://doi.org/10.1080/14760584.2023.2204154.

[17] La Torre G. Influenza and pneumococcal vaccination in hematological malignancies: a systematic review of efficacy, effectiveness and safety. Mediterr J Hematol Infect Dis 2016;8:e2016044. https://doi.org/10.4084/mjhid.2016.044.

[18] Lee ARYB, Wong SY, Chai LYA, Lee SC, Lee MX, Muthiah MD, et al. Efficacy of covid-19 vaccines in immunocompromised patients: systematic review and meta-analysis. BMJ 2022:e068632. https://doi.org/10.1136/bmj-2021-068632.

[19] Mangtani P, Evans SJW, Lange B, Oberle D, Smith J, Drechsel-Baeuerle U, et al. Safety profile of rubella vaccine administered to pregnant women: A systematic review of pregnancy related adverse events following immunisation, including congenital rubella syndrome and congenital rubella infection in the foetus or infant. Vaccine 2020;38:963–78. https://doi.org/10.1016/j.vaccine.2019.11.070.

[20] Mani A, Ojha V. Thromboembolism after COVID-19 Vaccination: A Systematic Review of Such Events in 286 Patients. Annals of Vascular Surgery 2022;84:12-20.e1. https://doi.org/10.1016/j.avsg.2022.05.001.

[21] McLaughlin JM, Jiang Q, Gessner BD, Swerdlow DL, Sings HL, Isturiz RE, et al. Pneumococcal conjugate vaccine against serotype 3 pneumococcal pneumonia in adults: A systematic review and pooled analysis. Vaccine 2019;37:6310–6. https://doi.org/10.1016/j.vaccine.2019.08.059.

[22] McMenamin ME, Bond HS, Sullivan SG, Cowling BJ. Estimation of Relative Vaccine Effectiveness in Influenza: A Systematic Review of Methodology. Epidemiology 2022;33:334–45. https://doi.org/10.1097/EDE.0000000000001473.

[23] McMenamin ME, Bond HS, Sullivan SG, Cowling BJ. Estimation of Relative Vaccine Effectiveness in Influenza: A Systematic Review of Methodology. Epidemiology 2022;33:334–45. https://doi.org/10.1097/EDE.0000000000001473.

[24] Mellone NG, Silva MT, Paglia MDG, Lopes LC, Barberato-Filho S, Del Fiol FDS, et al. Kawasaki Disease and the Use of the Rotavirus Vaccine in Children: A Systematic Review and Meta-Analysis. Front Pharmacol 2019;10:1075. https://doi.org/10.3389/fphar.2019.01075.

[25] Mohamed Hussein AAR, Ibrahim IH, Mahmoud IA, Amary M, Sayad R. To what extent AstraZeneca ChAdOx1 nCoV-19 vaccine is safe and effective? Rapid systematic review. Egypt J Bronchol 2022;16:6. https://doi.org/10.1186/s43168-021-00109-3.

[26] Najafi F, Sayehmiri K, Najafi R. Efficacy of Hepatitis B Vaccination in Under Five-Year-Old Children in Iran: A Systematic Review and Meta-Analysis Study. Hepat Mon 2018;18. https://doi.org/10.5812/hepatmon.65385.

[27] Nguyen H-T, Minar P, Jackson K, Fulkerson PC. Vaccinations in immunosuppressive-dependent pediatric inflammatory bowel disease. WJG 2017;23:7644–52. https://doi.org/10.3748/wjg.v23.i42.7644.

[28] Oueijan RI, Hill OR, Ahiawodzi PD, Fasinu PS, Thompson DK. Rare Heterogeneous Adverse Events Associated with mRNA-Based COVID-19 Vaccines: A Systematic Review. Medicines 2022;9:43. https://doi.org/10.3390/medicines9080043.

[29] Pollard SL, Malpica-Llanos T, Friberg IK, Fischer-Walker C, Ashraf S, Walker N. Estimating the herd immunity effect of rotavirus vaccine. Vaccine 2015;33:3795–800. https://doi.org/10.1016/j.vaccine.2015.06.064.

[30] Rawal S, Tackett RL, Stone RH, Young HN. COVID-19 vaccination among pregnant people in the United States: a systematic review. American Journal of Obstetrics & Gynecology MFM 2022;4:100616. https://doi.org/10.1016/j.ajogmf.2022.100616.

[31] Rimmer MP, Teh JJ, Mackenzie SC, Al Wattar BH. The risk of miscarriage following COVID-19 vaccination: a systematic review and meta-analysis. Human Reproduction 2023;38:840–52. https://doi.org/10.1093/humrep/dead036.

[32] Rondaan C, Furer V, Heijstek MW, Agmon-Levin N, Bijl M, Breedveld FC, et al. Efficacy, immunogenicity and safety of vaccination in adult patients with autoimmune inflammatory rheumatic diseases: a systematic literature review for the 2019 update of EULAR recommendations. RMD Open 2019;5:e001035. https://doi.org/10.1136/rmdopen-2019-001035.

[33] Schönberger K, Kirchgässner K, Riedel C, Von Kries R. Effectiveness of 2+1 PCV7 vaccination schedules in children under 2 years: A meta-analysis of impact studies. Vaccine 2013;31:5948–52. https://doi.org/10.1016/j.vaccine.2013.10.042.

[34] Shafie'ei M, Jamali M, Akbari Z, Sarvipour N, Ahmadzade M, Ahramiyanpour N. Cutaneous adverse reactions following COVID-19 vaccinations: A systematic review and meta-analysis. J of Cosmetic Dermatology 2022;21:3636–50. https://doi.org/10.1111/jocd.15261.

[35] Sousa S, Duarte AC, Cordeiro I, Ferreira J, Gonçalves MJ, Meirinhos T, et al. Efficacy and Safety of Vaccination in Pediatric Patients with Systemic Inflammatory Rheumatic Diseases: a systematic review of the literature. Acta Reumatol Port 2017;42:8–16.

[36] Teerawattananon Y, Anothaisintawee T, Pheerapanyawaranun C, Botwright S, Akksilp K, Sirichumroonwit N, et al. A systematic review of methodological approaches for evaluating real-

world effectiveness of COVID-19 vaccines: Advising resource-constrained settings. PLoS ONE 2022;17:e0261930. https://doi.org/10.1371/journal.pone.0261930.

[37] Tin Tin Htar M, Jackson S, Balmer P, Serra LC, Vyse A, Slack M, et al. Systematic literature review of the impact and effectiveness of monovalent meningococcal C conjugated vaccines when used in routine immunization programs. BMC Public Health 2020;20:1890. https://doi.org/10.1186/s12889-020-09946-1.

[38] Tzenios N, Tazanios ME, Chahine M. Combining Influenza and COVID-19 Booster Vaccination Strategy to Improve Vaccination Uptake Necessary for Managing the Health Pandemic: A Systematic Review and Meta-Analysis. Vaccines 2022;11:16. https://doi.org/10.3390/vaccines11010016.

[39] Wang J, Tong Y, Li D, Li J, Li Y. The Impact of Age Difference on the Efficacy and Safety of COVID-19 Vaccines: A Systematic Review and Meta-Analysis. Front Immunol 2021;12:758294. https://doi.org/10.3389/fimmu.2021.758294.

[40] Wang LM, Cravo Oliveira Hashiguchi T, Cecchini M. Impact of vaccination on carriage of and infection by antibiotic-resistant bacteria: a systematic review and meta-analysis. Clin Exp Vaccine Res 2021;10:81. https://doi.org/10.7774/cevr.2021.10.2.81.

[41] Wigg De Araújo Lagos L, De Jesus Lopes De Abreu A, Caetano R, Braga JU. Yellow fever vaccine safety in immunocompromised individuals: a systematic review and meta-analysis. Journal of Travel Medicine 2023;30:taac095. https://doi.org/10.1093/jtm/taac095.

[42] Wu X, Wang L, Shen L, Tang K. Response of COVID-19 vaccination in multiple sclerosis patients following disease-modifying therapies: A meta-analysis. eBioMedicine 2022;81:104102. https://doi.org/10.1016/j.ebiom.2022.104102.

[43] Xu W, Tang J, Chen C, Wang C, Wen W, Cheng Y, et al. Safety and efficacy of the COVID-19 vaccine in children and/or adolescents:A meta-analysis. Journal of Infection 2022;84:722–46. https://doi.org/10.1016/j.jinf.2022.01.032.

[44] Zhan Y, Liu X, Feng Y, Wu S, Jiang Y. Safety and efficacy of human papillomavirus vaccination for people living with HIV: A systematic review and meta-analysis. Int J STD AIDS 2019;30:1105–15. https://doi.org/10.1177/0956462419852224.

[45] Zhang X, Xu X, Jin J. Rotavirus vaccination and the risk of type 1 diabetes and celiac disease: A systematic review and meta-analysis. Front Pediatr 2022;10:951127. https://doi.org/10.3389/fped.2022.951127.

[46] National Advisory Committee on Immunization. Public health level recommendations on the use of pneumococcal vaccines in adults, including the use of 15-valent and 20-valent conjugate vaccines. Ottawa: Public Health Agency of Canada; 2023 Feb. Available from: https://www.canada.ca/content/dam/phac-spc/documents/services/immunization/national-advisory-committee-on-immunization-naci/public-health-level-recommendations-use-pneumococcal-vaccines-adults-including-use-15-valent-20-valent-conjugate-vaccines/recommendations-use-pneumococcal-vaccines-adults-15-20-valent-conjugate.pdf.

Exclusion reason: No risk-of-bias assessment performed (n=38)

[1] Bansal A, Trieu M-C, Mohn KGI, Cox RJ. Safety, Immunogenicity, Efficacy and Effectiveness of Inactivated Influenza Vaccines in Healthy Pregnant Women and Children Under 5 Years: An Evidence-Based Clinical Review. Front Immunol 2021;12:744774. https://doi.org/10.3389/fimmu.2021.744774.

[2] Chaubey I, Vijay H, Govindaraj S, Babu H, Cheedarla N, Shankar EM, et al. Impact of COVID-19 Vaccination on Pregnant Women. Pathogens 2023;12:431. https://doi.org/10.3390/pathogens12030431.

[3] Chenchula S, Karunakaran P, Sharma S, Chavan M. Current evidence on efficacy of COVID-19 booster dose vaccination against the Omicron variant: A systematic review. Journal of Medical Virology 2022;94:2969–76. https://doi.org/10.1002/jmv.27697.

[4] Deleré Y, Wichmann O, Klug SJ, Sande MVD, Terhardt M, Zepp F, et al. The Efficacy and Duration of Vaccine Protection Against Human Papillomavirus. Deutsches Ärzteblatt International 2014. https://doi.org/10.3238/arztebl.2014.0584.

[5] Di Fusco M, Lin J, Vaghela S, Lingohr-Smith M, Nguyen JL, Scassellati Sforzolini T, et al. COVID-19 vaccine effectiveness among immunocompromised populations: a targeted literature review of real-world studies. Expert Review of Vaccines 2022;21:435–51. https://doi.org/10.1080/14760584.2022.2035222.

[6] Dr Amandeep Kaur, Dr Navdeep Kaur, Dr Preeti Singh Dhoat, Dr Mohit Madhukar. Systematic Review On Covid Vaccination In Children- Assessment Of Safety, Immunogenicity, Efficacy And Adverse Effects. Journal of Pharmaceutical Negative Results 2022:456–9. https://doi.org/10.47750/pnr.2022.13.S05.73.

[7] Feng S, Cowling BJ, Kelly H, Sullivan SG. Estimating Influenza Vaccine Effectiveness With the Test-Negative Design Using Alternative Control Groups: A Systematic Review and Meta-Analysis. American Journal of Epidemiology 2018;187:389–97. https://doi.org/10.1093/aje/kwx251.

[8] Fu Y, Zhao J, Wei X, Han P, Yang L, Ren T, et al. Effectiveness and Cost-Effectiveness of Inactivated Vaccine to Address COVID-19 Pandemic in China: Evidence From Randomized Control Trials and Real-World Studies. Front Public Health 2022;10:917732. https://doi.org/10.3389/fpubh.2022.917732.

[9] Gkentzi D, Katsakiori P, Marangos M, Hsia Y, Amirthalingam G, Heath PT, et al. Maternal vaccination against pertussis: a systematic review of the recent literature. Arch Dis Child Fetal Neonatal Ed 2017;102:F456–63. https://doi.org/10.1136/archdischild-2016-312341.

[10] Helena De Oliveira L, Jauregui B, Carvalho AF, Giglio N. Impact and effectiveness of meningococcal vaccines: a review. Revista Panamericana de Salud Pública 2017;41:1. https://doi.org/10.26633/RPSP.2017.158.

[11] Higdon MM, Wahl B, Jones CB, Rosen JG, Truelove SA, Baidya A, et al. A Systematic Review of Coronavirus Disease 2019 Vaccine Efficacy and Effectiveness Against Severe Acute Respiratory Syndrome Coronavirus 2 Infection and Disease. Open Forum Infectious Diseases 2022;9:ofac138. https://doi.org/10.1093/ofid/ofac138.

[12] Jonesteller CL, Burnett E, Yen C, Tate JE, Parashar UD. Effectiveness of Rotavirus Vaccination: A Systematic Review of the First Decade of Global Postlicensure Data, 2006–2016. Clinical Infectious Diseases 2017;65:840–50. https://doi.org/10.1093/cid/cix369.

[13] Karafillakis E, Hassounah S, Atchison C. Effectiveness and impact of rotavirus vaccines in Europe, 2006–2014. Vaccine 2015;33:2097–107. https://doi.org/10.1016/j.vaccine.2015.03.016.

[14] Khandker SS, Godman B, Jawad MdI, Meghla BA, Tisha TA, Khondoker MU, et al. A Systematic Review on COVID-19 Vaccine Strategies, Their Effectiveness, and Issues. Vaccines 2021;9:1387. https://doi.org/10.3390/vaccines9121387.

[15] Kyaw MH, Spinardi J, Zhang L, Oh HML, Srivastava A. Evidence synthesis and pooled analysis of vaccine effectiveness for COVID-19 mRNA vaccine BNT162b2 as a heterologous booster after inactivated SARS-CoV-2 virus vaccines. Human Vaccines & Immunotherapeutics 2023;19:2165856. https://doi.org/10.1080/21645515.2023.2165856.

[16] Lall D, Cason E, Pasquel FJ, Ali MK, Narayan KMV. Effectiveness of Influenza Vaccination for Individuals with Chronic Obstructive Pulmonary Disease (COPD) in Low- and Middle-Income Countries. COPD: Journal of Chronic Obstructive Pulmonary Disease 2016;13:93–9. https://doi.org/10.3109/15412555.2015.1043518.

[17] Lee CJ, Woo W, Kim AY, Yon DK, Lee SW, Koyanagi A, et al. Clinical manifestations of COVID-19 breakthrough infections: A systematic review and meta-analysis. Journal of Medical Virology 2022;94:4234–45. https://doi.org/10.1002/jmv.27871.

[18] Malik SA, Modarage K, Goggolidou P. A systematic review assessing the effectiveness of COVID-19 mRNA vaccines in chronic kidney disease (CKD) individuals. F1000Res 2022;11:909. https://doi.org/10.12688/f1000research.122820.1.

[19] Mallory RM, Bandell A, Ambrose CS, Yu J. A systematic review and meta-analysis of the effectiveness of LAIV4 and IIV in children aged 6 months to 17 years during the 2016–2017 season. Vaccine 2020;38:3405–10. https://doi.org/10.1016/j.vaccine.2019.12.015.

[20] Manske JM. Efficacy and Effectiveness of Maternal Influenza Vaccination During Pregnancy: A Review of the Evidence. Matern Child Health J 2014;18:1599–609. https://doi.org/10.1007/s10995-013-1399-2.

[21] Mariani L, Vici P, Suligoi B, Checcucci-Lisi G, Drury R. Early Direct and Indirect Impact of Quadrivalent HPV (4HPV) Vaccine on Genital Warts: a Systematic Review. Adv Ther 2015;32:10–30. https://doi.org/10.1007/s12325-015-0178-4.

[22] Marin M, Marti M, Kambhampati A, Jeram SM, Seward JF. Global Varicella Vaccine Effectiveness: A Meta-analysis. Pediatrics 2016;137:e20153741. https://doi.org/10.1542/peds.2015-3741.

[23] Middleton BF, Danchin M, Fathima P, Bines JE, Macartney K, Snelling TL. Review of the health impact of the oral rotavirus vaccine program in children under 5 years in Australia: 2006 – 2021. Vaccine 2023;41:636–48. https://doi.org/10.1016/j.vaccine.2022.12.008.

[24] Mohammed I, Nauman A, Paul P, Ganesan S, Chen K-H, Jalil SMS, et al. The efficacy and effectiveness of the COVID-19 vaccines in reducing infection, severity, hospitalization, and

mortality: a systematic review. Human Vaccines & Immunotherapeutics 2022;18:2027160. https://doi.org/10.1080/21645515.2022.2027160.

[25] Okoli GN, Racovitan F, Righolt CH, Mahmud SM. Variations in Seasonal Influenza Vaccine Effectiveness due to Study Characteristics: A Systematic Review and Meta-analysis of Test-Negative Design Studies. Open Forum Infectious Diseases 2020;7:ofaa177. https://doi.org/10.1093/ofid/ofaa177.

[26] Paybast S, Emami A, Baghalha F, Naser Moghadasi A. Watch out for neuromyelitis optica spectrum disorder onset or clinical relapse after COVID-19 vaccination: What neurologists need to know? Multiple Sclerosis and Related Disorders 2022;65:103960. https://doi.org/10.1016/j.msard.2022.103960.

[27] Rondy M, El Omeiri N, Thompson MG, Levêque A, Moren A, Sullivan SG. Effectiveness of influenza vaccines in preventing severe influenza illness among adults: A systematic review and meta-analysis of test-negative design case-control studies. Journal of Infection 2017;75:381–94. https://doi.org/10.1016/j.jinf.2017.09.010.

[28] Sullivan SG, Feng S, Cowling BJ. Potential of the test-negative design for measuring influenza vaccine effectiveness: a systematic review. Expert Review of Vaccines 2014;13:1571–91. https://doi.org/10.1586/14760584.2014.966695.

[29] Switzer C, D'Heilly C, Macina D. Immunological and Clinical Benefits of Maternal Immunization Against Pertussis: A Systematic Review. Infect Dis Ther 2019;8:499–541. https://doi.org/10.1007/s40121-019-00264-7.

[30] Tang K-T, Hsu B-C, Chen D-Y. Immunogenicity, Effectiveness, and Safety of COVID-19 Vaccines in Rheumatic Patients: An Updated Systematic Review and Meta-Analysis. Biomedicines 2022;10:834. https://doi.org/10.3390/biomedicines10040834.

[31] Velázquez RF, Linhares AC, Muñoz S, Seron P, Lorca P, DeAntonio R, et al. Efficacy, safety and effectiveness of licensed rotavirus vaccines: a systematic review and meta-analysis for Latin America and the Caribbean. BMC Pediatr 2017;17:14. https://doi.org/10.1186/s12887-016-0771-y.

[32] Wang W (Vivian), Kothari S, Skufca J, Giuliano AR, Sundström K, Nygård M, et al. Real-world impact and effectiveness of the quadrivalent HPV vaccine: an updated systematic literature review. Expert Review of Vaccines 2022;21:1799–817. https://doi.org/10.1080/14760584.2022.2129615.

[33] Waure CD. Effectiveness of 7-valent pneumococcal conjugate vaccine: A meta-analysis of post-marketing studies. WJMA 2015;3:151. https://doi.org/10.13105/wjma.v3.i3.151.

[34] Wilkinson K, Righolt CH, Elliott LJ, Fanella S, Mahmud SM. Pertussis vaccine effectiveness and duration of protection – A systematic review and meta-analysis. Vaccine 2021;39:3120–30. https://doi.org/10.1016/j.vaccine.2021.04.032.

[35] Xu M, Liu C, Du Z, Bai Y, Wang Z, Gao C. Real-world effectiveness of monkeypox vaccines: a systematic review. Journal of Travel Medicine 2023;30:taad048. https://doi.org/10.1093/jtm/taad048.

[36] Zhang J, Yang W, Huang F, Zhang K. Effectiveness of mRNA and viral-vector vaccines in epidemic period led by different SARS-CoV-2 variants: A systematic review and meta-analysis. Journal of Medical Virology 2023;95:e28623. https://doi.org/10.1002/jmv.28623.

[37] Public Health Agency of Canada. Public health level recommendations on the use of pneumococcal vaccines in adults, including the use of 15-valent and 20-valent conjugate vaccines [Internet]. Ottawa (ON): Government of Canada; 2023 Feb. Available from: https://publications.gc.ca/collections/collection_2023/aspc-phac/HP5-153-1-2023-eng.pdf n.d.

[38] Towards the elimination of cervical cancer: HPV epidemiology, real-world experiences and the potential impact of the 9-valent HPV vaccine. EJGO 2021;42:1068. https://doi.org/10.31083/j.ejgo4205156.

Exclusion reason: Conference abstract (n=36)

[1] Childs L, Kobayashi M, Farrar JL, Pilishvili T. 13. The Efficacy and Effectiveness of Pneumococcal Vaccines against Pneumococcal Pneumonia among Adults: A Systematic Review and Meta-Analysis. Open Forum Infectious Diseases 2021;8:S130–1. https://doi.org/10.1093/ofid/ofab466.215.

[2] Chung. Chung G, Doan T, Hutton DW. A systematic review of effectiveness and cost-effectiveness studies of vaccination for respiratory syncytial virus. Med Decis Mak. 2020;40(5):E408-E409. n.d.

[3] Covington. Covington D, Kaydo S, Veley K. Hepatitis B virus (HBV) vaccine in pregnancy and impact on pregnancy outcome. Value Health. 2018;21(Suppl 1):S151. n.d.

[4] Dooling K, Guo A, Leung J, Belongia E, Harpaz R. Performance of Zoster Vaccine Live (Zostavax): A Systematic Review of 12 years of Experimental and Observational Evidence. Open Forum Infectious Diseases 2017;4:S412–3. https://doi.org/10.1093/ofid/ofx163.1033.

[5] Farrar JL, Kobayashi M, Childs L, Pilishvili T. 21. Systematic Review and Meta-Analysis of Pneumococcal Vaccine Effectiveness against Invasive Pneumococcal Disease among Adults. Open Forum Infectious Diseases 2021;8:S134–5. https://doi.org/10.1093/ofid/ofab466.223.

[6] Johnson. Johnson KD, Jiang Y, Weiss T, Graham J. Herpes zoster vaccine effectiveness and waning of effectiveness. Value Health. 2017;20(5):A326. n.d.

[7] Lee JKH, Lam GKL, Shin T, Samson SI, Greenberg DP, Chit A. 2745. Efficacy and Effectiveness of High-Dose Influenza Vaccine for Older Adults by Circulating Strain and Antigenic Match: A Systematic Review and Meta-Analysis. Open Forum Infectious Diseases 2019;6:S966–7. https://doi.org/10.1093/ofid/ofz360.2422.

[8] Lee JK, Lam GK, Vaisman R, Yin KJ, Seet BT, Loiacono MM, et al. 102. Efficacy and Effectiveness of High-Dose Influenza Vaccine in Older Adults by Age and Seasonal Characteristics: An Updated Systematic Review and Meta-Analysis. Open Forum Infectious Diseases 2022;9:ofac492.180. https://doi.org/10.1093/ofid/ofac492.180.

[9] Lee J, Lam G, Shin T, Kim J, Krishnan A, Seet B, et al. Efficacy and Effectiveness of High-Dose Influenza Vaccine for Older Adults: A Systematic Review and Meta-Analysis. Open Forum Infectious Diseases 2017;4:S456–S456. https://doi.org/10.1093/ofid/ofx163.1161.

[10] Li S, Kuter BJ, Schmidt E, Richardson E, Saldutti LP, Monika N, et al. 1390. Effectiveness of M-M-R® II in outbreaks - a systematic literature review of real-world observational studies. Open Forum Infectious Diseases 2020;7:S704–5. https://doi.org/10.1093/ofid/ofaa439.1572.

[11] Lim WW, Cowling BJ, Chan MCW. The determinants of influenza vaccination effectiveness in adults aged 65 years and older: a systematic review. International Journal of Infectious Diseases 2016;53:147. https://doi.org/10.1016/j.ijid.2016.11.360.

[12] Mathew JL, Singhi S. Current status of pneumococcal protein vaccines: A systematic review of literature. International Journal of Infectious Diseases 2014;21:433–4. https://doi.org/10.1016/j.ijid.2014.03.1314.

[13] Miltz A, Price H, Shahmanesh M, Copas A, Gilson R. P3.372 Systematic Review and Meta-Analysis of L1-VLP-based Human Papillomavirus Vaccine Efficacy Against Anogenital Pre-Cancer in Women with Evidence of Prior HPV Exposure. Sex Transm Infect 2013;89:A265.3-A266. https://doi.org/10.1136/sextrans-2013-051184.0825.

[14] Nowak. Nowak O, Boronea B, John T, Shang J, Parihar H. Effectiveness of influenza vaccination in patients with diabetes: A systematic review. J Manag Care Spec Pharm. 2018;24(4-A Suppl.):S39. n.d.

[15] Okoli G, Racovitan F, Righolt C, Mahmud S. Variable seasonal influenza vaccine effectiveness between regions, and across age groups: Evidence from test-negative design studies. International Journal of Infectious Diseases 2020;101:486. https://doi.org/10.1016/j.ijid.2020.09.1269.

[16] Perdrizet J, Pustulka I, Forbes CA, Horn E, Gessner BD, Hayford K. 586. Systematic Literature Review of the 13-valent Pneumococcal Conjugate Vaccine (PCV13) Effectiveness Against Invasive Pneumococcal Disease in Children Globally. Open Forum Infectious Diseases 2022;9:ofac492.638. https://doi.org/10.1093/ofid/ofac492.638.

[17] Saddier P, Marks MA, Calhoun S, Johnson K, Moride Y. 2480. Real-World Effectiveness of the Live Zoster Vaccine in Preventing Herpes Zoster: A Systematic Review. Open Forum Infectious Diseases 2018;5:S743–S743. https://doi.org/10.1093/ofid/ofy210.2133.

[18] Scharer S, Pollock A, Sevcikova P. PP26 Efficacy of HPV vaccines: a review of the evidence used by the WHO. Oral Presentations, BMJ Publishing Group Ltd; 2014, p. A57.2-A58. https://doi.org/10.1136/jech-2014-204726.122.

[19] Schmitz S, Usher C, Adams R, Kieran J, Barry M, Walsh C. Meta-analysis of BCG Vaccine Efficacy for Infants in Ireland. Value in Health 2013;16:A331. https://doi.org/10.1016/j.jval.2013.08.050.

[20] Sim. Sim JJ, Lim CC. Influenza vaccination in systemic lupus erythematosus (SLE): Effectiveness, efficacy, safety, utilization, and barriers. J Am Soc Nephrol. 2021;32:501. n.d.

[21] Steinberg. Steinberg A, Bendall A. Environmental substitutability reimagining registrar education—introducing the advanced trainee network. Nephrology. 2020;25(Suppl 3):40–82. Nephrology 2020;25:40–82. https://doi.org/10.1111/nep.13799.

[22] Tchimbakala. Tchimbakala D, Casanova F. Effectiveness of influenza vaccines among diabetes population: A systematic review. Exercer. 2015;26(121):38S-39S. n.d.

[23] Tejada. Tejada RA, Vargas KG, Benites-Zapata V, Mezones-Holgui E, Hernandez AV, Bolanos R. Efficacy of HPV vaccines against non-cancer lesions: Systematic review and meta-analysis. Int J Epidemiol. 2015;44:164-164. n.d.

[24] Vasileiou E, Sheikh A, Butler C, Ferkh KE, Simpson C. P133 Safety and effectiveness of influenza vaccines in people with asthma: a systematic review and meta-analysis. Thorax 2016;71:A155.1-A155. https://doi.org/10.1136/thoraxjnl-2016-209333.276.

[25] Vasileiou E, Simpson C, Sheikh A, Butler C. Seasonal influenza vaccine effectiveness in people with asthma: A systematic review. 5.3 Allergy and Immunology, European Respiratory Society; 2016, p. PA4205. https://doi.org/10.1183/13993003.congress-2016.PA4205.

[26] Meybodi MA, SeyedAlinaghi S, Mirzaei M, et al. Hepatitis B vaccination in chronic kidney disease patients: A systematic review and meta-analysis. J Hepatol. 2022;77(3):626-635. doi:10.1016/S0016-5085(22)06385-0. n.d.

[27] NicLochlainn M, McCarthy M, et al. Vaccine effectiveness against influenza in adults with comorbidities: A systematic review and meta-analysis. Clin Microbiol Infect. 2017;23(6):399-405. doi:10.1111/1365-3156. n.d.

[28] Bozat-Emre S, Ye X, Morrow A, Casaclang N, Mahmud SM. Effectiveness of the 2009 pandemic H1N1 influenza vaccines in preventing H1N1 infection: A meta-analysis. Pharmacoepidemiol Drug Saf. 2014;23(Suppl 1):161-162. doi:10.1002/pds.3701. Pharmacoepidemiology and Drug 2014;23:1–497. https://doi.org/10.1002/pds.3701.

[29] Okoli GN, Racovitan F, Righolt CH, Mahmud SM. Influence of comorbidity status on seasonal influenza vaccine effectiveness in Canada: A systematic review and meta-analysis of test-negative design studies. Pharmacoepidemiol Drug Saf. 2019;28(Suppl 2):387-388. doi:10.1002/pds.4864. Pharmacoepidemiology and Drug 2019;28:5–586. https://doi.org/10.1002/pds.4864.

[30] Okoli GN, Racovitan F, Righolt CH, Mahmud SM. Variations in seasonal influenza vaccine effectiveness: A systematic review and meta-analysis of test-negative design studies. Pharmacoepidemiol Drug Saf. 2019;28(Suppl 2):449. doi:10.1002/pds.4864. Pharmacoepidemiology and Drug 2019;28:5–586. https://doi.org/10.1002/pds.4864.

[31] Adetokunboh O, Ndwandwe D, Awotiwon A, Uthman OA, Wiysonge CS. Systematic review and meta-analysis of vaccine efficacy and effectiveness among HIV-infected, HIV-exposed uninfected, and HIV-uninfected children. Trans R Soc Trop Med Hyg. 2019;113(Suppl 1):S66. doi:10.1093/trstmh/trz094. Transactions of The Royal Society of Tropical Medicine and Hygiene 2019;113:S1–98. https://doi.org/10.1093/trstmh/trz094.

[32] Okoli G, Racovitan F, Righolt C, Mahmud S. Seasonal influenza vaccine effectiveness: Evidence from test-negative design studies. Pharmacoepidemiol Drug Saf. 2020;29(Suppl 3):631. doi:10.1002/pds.5114. Pharmacoepidemiology and Drug 2020;29:3–634. https://doi.org/10.1002/pds.5114.

[33] Hamad M, Allam H, Sulaiman A, Cheikh Hassan HI. Herpes zoster vaccine effectiveness in patients with chronic kidney disease: Systematic review of the literature and meta-analysis. Nephrology. 2020;25(Suppl 3):47. doi:10.1111/nep.13799. Nephrology 2020;25:40–82. https://doi.org/10.1111/nep.13799.

[34] Prasad S, Kalafat E, Blakeway H, Townsend R, O'Brien P, Morris E, Draycott T, Thangaratinam S, Le Doare K, Ladhani S, Von Dadelszen P, Magee LA, Heath P, Khalil A. Effectiveness and perinatal outcomes of COVID-19 vaccination in pregnancy: Systematic review and meta-analysis. BJOG. 2022;129(Suppl 1):144. doi:10.1111/1471-0528.18_17178. BJOG 2022;129:141–54. https://doi.org/10.1111/1471-0528.18_17178.

[35] Villavicencio A, Ebisu Y, Raja M, Sanchez-Covarrubias AP, Anjan S, Reynolds JM, Simkins J, Camargo J, Morris MI, Abbo L, Guerra G, Natori Y. Immunogenicity and safety of SARS-CoV-2 vaccines and breakthrough infections among solid organ transplant recipients: Systematic review and meta-analysis. Am J Transplant. 2022;22(Suppl 3):769-770. doi:10.1111/ajt.17073. American Journal of Transplantation 2022;22:607–1136. https://doi.org/10.1111/ajt.17073.

[36] Mhanna M, Abuhelwa Z, Sayeh W, Assaly R, Beran A. Effectiveness of SARS-CoV-2 vaccination in patients with liver cirrhosis: A systematic review and meta-analysis. Hepatology. 2022;76(Suppl 1):S548. doi:10.1002/hep.32697. Hepatology 2022;76:S1–1564. https://doi.org/10.1002/hep.32697.

Exclusion reason: Not a systematic review (n=23)

[1] Ahern S, Walsh KA, Paone S, Browne J, Carrigan M, Harrington P, et al. Clinical efficacy and effectiveness of alternative varicella vaccination strategies: An overview of reviews. Reviews in Medical Virology 2023;33:e2407. https://doi.org/10.1002/rmv.2407.

[2] Belongia EA, Simpson MD, King JP, Sundaram ME, Kelley NS, Osterholm MT, et al. Variable influenza vaccine effectiveness by subtype: a systematic review and meta-analysis of test-negative design studies. The Lancet Infectious Diseases 2016;16:942–51. https://doi.org/10.1016/S1473-3099(16)00129-8.

[3] Calabrò GE, Boccalini S, Panatto D, Rizzo C, Di Pietro ML, Abreha FM, et al. The New Quadrivalent Adjuvanted Influenza Vaccine for the Italian Elderly: A Health Technology Assessment. IJERPH 2022;19:4166. https://doi.org/10.3390/ijerph19074166.

[4] Demurtas J, Celotto S, Beaudart C, Sanchez-Rodriguez D, Balci C, Soysal P, et al. The efficacy and safety of influenza vaccination in older people: An umbrella review of evidence from meta-analyses of both observational and randomized controlled studies. Ageing Research Reviews 2020;62:101118. https://doi.org/10.1016/j.arr.2020.101118.

[5] Feng S, Cowling BJ, Sullivan SG. Influenza vaccine effectiveness by test-negative design – Comparison of inpatient and outpatient settings. Vaccine 2016;34:1672–9. https://doi.org/10.1016/j.vaccine.2016.02.039.

[6] Fridrich J. The Efficacy and Duration of Vaccine Protection Against Human Papillomavirus: A Systematic Review and Meta-analysis. Deutsches Ärzteblatt International 2015. https://doi.org/10.3238/arztebl.2015.0209a.

[7] Harder T, Remschmidt C, Haller S, Eckmanns T, Wichmann O. Use of existing systematic reviews for evidence assessments in infectious disease prevention: a comparative case study. Syst Rev 2016;5:171. https://doi.org/10.1186/s13643-016-0347-9.

[8] Ioannidis JPA. Factors influencing estimated effectiveness of COVID-19 vaccines in non-randomised studies. BMJ EBM 2022;27:324–9. https://doi.org/10.1136/bmjebm-2021-111901.

[9] Kraicer-Melamed H, O'Donnell S, Quach C. Corrigendum to "The effectiveness of pneumococcal polysaccharide vaccine 23 (PPV23) in the general population of 50 years of age and older: A systematic review and meta-analysis" [Vaccine 34 (2016) 1540–1550]. Vaccine 2016;34:4083–4. https://doi.org/10.1016/j.vaccine.2016.06.045.

[10] Mathew JL, Mitra M. Systematic review of effectiveness of varicella vaccines: A critical appraisal. Indian Pediatr 2016;53:418–22. https://doi.org/10.1007/s13312-016-0865-5.

[11] Meggiolaro A, Sane Schepisi M, Farina S, Castagna C, Mammone A, Siddu A, et al. Effectiveness of vaccination against SARS-CoV-2 Omicron variant infection, symptomatic disease, and hospitalization: a systematic review and meta-analysis. Expert Review of Vaccines 2022;21:1831–41. https://doi.org/10.1080/14760584.2022.2130773.

[12] Niederman MS, Folaranmi T, Buchwald UK, Musey L, Cripps AW, Johnson KD. Efficacy and effectiveness of a 23-valent polysaccharide vaccine against invasive and noninvasive pneumococcal disease and related outcomes: a review of available evidence. Expert Review of Vaccines 2021;20:243–56. https://doi.org/10.1080/14760584.2021.1880328.

[13] Okoli GN, Reddy VK, Lam OLT, Racovitan F, Al-Yousif Y, Askin N. Characteristics and methodological standards across systematic reviews with Meta-analysis of efficacy and/or effectiveness of influenza vaccines: an overview of reviews. Infectious Diseases 2022;54:861–80. https://doi.org/10.1080/23744235.2022.2114537.

[14] Ostropolets A, Hripcsak G. COVID-19 vaccination effectiveness rates by week and sources of bias: a retrospective cohort study. BMJ Open 2022;12:e061126. https://doi.org/10.1136/bmjopen-2022-061126.

[15] Remschmidt C, Wichmann O, Harder T. Methodological quality of systematic reviews on influenza vaccination. Vaccine 2014;32:1678–84. https://doi.org/10.1016/j.vaccine.2014.01.060.

[16] Sæterdal I, Couto E, Juvet L, Harboe I, Klemp M. Effect of Catch-Up HPV Vaccination of Young Women. Oslo, Norway: Knowledge Centre for the Health Services at The Norwegian Institute of Public Health (NIPH); 2014.

[17] Sudarsanam TD, Tharyan P. Vaccines for preventing pneumococcal infection in adults. Summary of the evidence and implications for public health programmes. Clinical Epidemiology and Global Health 2014;2:133–7. https://doi.org/10.1016/j.cegh.2014.03.003.

[18] Schroll JB. Human papilloma virus vaccination and its real-life efficacy. Acta Obstet Gynecol Scand 2020;99:1267–8. https://doi.org/10.1111/aogs.13964.

[19] Trucchi C, Paganino C, Orsi A, De Florentis D, Ansaldi F. Influenza vaccination in the elderly: why are the overall benefits still hotly debated? Journal of Preventive Medicine and Hygiene 2015;Vol 56:37-43 Pages. https://doi.org/10.15167/2421-4248/JPMH2015.56.1.474.

[20] Loeb M. Community-acquired pneumonia: pneumococcal vaccine. BMJ Clin Evid. 2015.

[21] Kolber MR, Lau D, Eurich D, Korownyk C. Effectiveness of the trivalent influenza vaccine. Can Fam Physician. 2014;60(1):50. n.d.

[22] Corrigendum to: Effectiveness of 13-Valent Pneumococcal Conjugate Vaccine Against Invasive Disease Caused by Serotype 3 in Children: A Systematic Review and Meta-analysis of Observational Studies. Clinical Infectious Diseases 2021;72:1684–5. https://doi.org/10.1093/cid/ciaa1767.

[23] Feng S, Cowling BJ, Sullivan SG. Influenza vaccine effectiveness by test-negative design – Comparison of inpatient and outpatient settings. Vaccine 2016;34:1672–9. https://doi.org/10.1016/j.vaccine.2016.02.039.

Exclusion reason: Not peer reviewed (n=19)

[1] Byambasuren O, Stehlik P, Clark J, Alcorn K, Glasziou P. Impact of COVID-19 vaccination on long COVID: a systematic review and meta-analysis 2022. https://doi.org/10.1101/2022.06.20.22276621.

[2] Farrar JL, Childs L, Ouattara M, Akhter F, Britton A, Pilishvili T, et al. Systematic Review and Meta-Analysis of the Efficacy and Effectiveness of Pneumococcal Vaccines in Adults 2022. https://doi.org/10.1101/2022.10.06.22280772.

[3] Ghazy RM, Ashmawy R, Hamdy NA, Elhadi YAM, Reyad OA, Almalawany D, et al. Efficacy and Effectiveness of SARS-CoV-2 vaccine: A systematic review and a meta-analysis. 2021. https://doi.org/10.21203/rs.3.rs-936074/v1.

[4] Katoto PD, Kakubu MA, Tamuzi JL, Brand AS, Ayuk A, Byamungu LN, et al. Immunogenicity, Safety and Effectiveness of COVID-19 Pfizer-BioNTech (BNT162b2) mRNA Vaccination in Immunocompromised Adolescents and Young Adults: A systematic Review and Meta-Analyses 2023. https://doi.org/10.1101/2023.01.20.23284812.

[5] Li Y, Liang H, Ding X, Cao Y, Yang D, Duan Y. Effectiveness of COVID-19 vaccine in children and adolescents with the Omicron variant: A systematic review and meta-analysis. Journal of Infection 2023;86:e64–6. https://doi.org/10.1016/j.jinf.2023.01.001.

[6] Meggiolaro A, Schepisi MS, Farina S, Castagna C, Mammone A, Siddu A, et al. Effectiveness of vaccination against SARS-CoV-2 Omicron variant infection, symptomatic disease, and hospitalisation: a systematic review and meta-analysis 2022. https://doi.org/10.1101/2022.06.23.22276809.

[7] Menegale F, Manica M, Zardini A, Guzzetta G, Marziano V, d'Andrea V, et al. Waning of SARS-CoV-2 vaccine-induced immunity: A systematic review and secondary data analysis 2022. https://doi.org/10.1101/2022.07.04.22277225.

[8] Ostropolets A, Hripcsak G. COVID-19 vaccination effectiveness rates by week and sources of bias 2021. https://doi.org/10.1101/2021.12.22.21268253.

[9] Rahmani K, Shavaleh R, Forouhi M, Disfani HF, Kamandi M, Zadegan Dezfuli AA, et al. Effectiveness of COVID-19 Vaccines and Post-vaccination SARS-COV 2 Infection, Hospitalization, and Mortality: a Systematic Review and Meta-analysis of Observational Studies 2021. https://doi.org/10.1101/2021.11.03.21265819.

[10] Schepisi MS. Early real world evidence on the relative SARS-COV-2 vaccine effectiveness of bivalent COVID-19 booster doses: a rapid review 2023. https://doi.org/10.1101/2023.03.28.23287762.

[11] Shi A, Tang X, Xia P, Hao M, Shu Y, Nakanishi H, et al. Cardiac Arrhythmia after COVID-19 Vaccination versus Non–COVID-19 Vaccination: A Systematic Review and Meta-Analysis 2022. https://doi.org/10.1101/2022.11.21.22282554.

[12] Song S, Madewell ZJ, Liu M, Longini IM, Yang Y. Effectiveness of SARS-CoV-2 Vaccines against Omicron Infection and Severe Events: A Systematic Review and Meta-Analysis of Test-Negative Design Studies 2023. https://doi.org/10.1101/2023.02.16.23286041.

[13] Tamuzi JL, Muyaya LM, Mitra A, Nyasulu PS. Systematic review and meta-analysis of COVID-19 vaccines safety, tolerability, and efficacy among HIV-infected patients 2022. https://doi.org/10.1101/2022.01.11.22269049.

[14] Tsang TK, Sullivan SG, Huang X, Wang C, Wang Y, Nealon J, et al. Prior infections and effectiveness of SARS-CoV-2 vaccine in test-negative study: A systematic review and meta-analysis 2022. https://doi.org/10.1101/2022.11.03.22281925.

[15] Van Werkhoven CH (Henri), De Gier B, McDonald S, De Melker HE, Hahné SJM, Van Den Hof S, et al. Information bias of vaccine effectiveness estimation due to informed consent for national registration of COVID-19 vaccination: estimation and correction using a data augmentation model 2023. https://doi.org/10.1101/2023.05.23.23290384.

[16] Rahman MO, Kamigaki T, Thandar MM, Haruyama R, Yan F, Shibamura-Fujiogi M, et al. Effectiveness of the booster doses of mRNA vaccine against SARS-CoV-2 Omicron infection and its severe outcomes: A systematic review and meta-analysis [Preprint]. SSRN. 2023. Available from: https://ssrn.com/abstract=4445180.

[17] World Health Organization. Global research on coronavirus disease (COVID-19) [Internet]. Geneva: WHO. Available from: https://www.who.int/emergencies/diseases/novel-coronavirus-2019/global-research-on-novel-coronavirus-2019-ncov n.d.

[18] Xu AY, Pang H. Meta-analysis of documents on vaccine effectiveness of live attenuated varicella vaccine in pupils and preschoolers. Chin J Biol. 2019;32(5):557-64. doi: 10.13200/j.cnki.cjb.002.

[19] Zhang ZJZ, Suo LD, Zhao D, Pan JB, Lu L. Systematic reviews and evidence quality assessment on effectiveness of 1 dose varicella attenuated live vaccine for healthy children aged 1-12 years in China. Zhonghua Liu Xing Bing Xue Za Zhi. 2020;41(7):1138-44. doi: 10.3760/cma.j.cn112338-20191025-00762.

Exclusion reason: Wrong study design (n=11)

[1]. World Health Organization. Global research on coronavirus disease (COVID-19). Available from: https://www.who.int/emergencies/diseases/novel-coronavirus-2019/global-research-on-novel-coronavirus-2019-ncov

[2]. European Centre for Disease Prevention and Control. COVID-19. Available from: https://www.ecdc.europa.eu/en/covid-19-pandemic

[3]. World Health Organization. Global research on coronavirus disease (COVID-19). Available from: https://www.who.int/emergencies/diseases/novel-coronavirus-2019/global-research-on-novel-coronavirus-2019-ncov

[4]. World Health Organization. Global research on coronavirus disease (COVID-19). Available from: https://www.who.int/emergencies/diseases/novel-coronavirus-2019/global-research-on-novel-coronavirus-2019-ncov

[5]. World Health Organization. Global research on coronavirus disease (COVID-19). Available from: https://www.who.int/emergencies/diseases/novel-coronavirus-2019/global-research-on-novel-coronavirus-2019-ncov

[6]. World Health Organization. Global research on coronavirus disease (COVID-19). Available from: https://www.who.int/emergencies/diseases/novel-coronavirus-2019/global-research-on-novel-coronavirus-2019-ncov

[7]. World Health Organization. Global research on coronavirus disease (COVID-19). Available from: https://www.who.int/emergencies/diseases/novel-coronavirus-2019/global-research-on-novel-coronavirus-2019-ncov

[8]. Health Technology Wales. Coronavirus (COVID-19). Available from: https://www.healthtechnologywales.org.uk/covid-19/

[9]. McMaster University. COVID-19 Rapid Evidence Reviews. Available from: https://www.mcmaster.ca/covid-19-rapid-evidence-reviews

[10]. World Health Organization. Global research on coronavirus disease (COVID-19). Available from: https://www.who.int/emergencies/diseases/novel-coronavirus-2019/global-research-on-novel-coronavirus-2019-ncov

[11]. National Center for Biotechnology Information, U.S. National Library of Medicine. LitCOVID. Available from: https://www.ncbi.nlm.nih.gov/research/coronavirus/

Lack of inclusion of observational study design (n=8)

[1] García-Perdomo HA, Osorio JC, Fernandez A, Zapata-Copete JA, Castillo A. The effectiveness of vaccination to prevent the papillomavirus infection: a systematic review and meta-analysis. Epidemiol Infect 2019;147:e156. https://doi.org/10.1017/S0950268818003679.

[2] Glenn DA, Hegde A, Kotzen E, Walter EB, Kshirsagar AV, Falk R, et al. Systematic Review of Safety and Efficacy of COVID-19 Vaccines in Patients With Kidney Disease. Kidney International Reports 2021;6:1407–10. https://doi.org/10.1016/j.ekir.2021.02.011.

[3] Haghshenas M, Mousavi T, Kheradmand M, Afshari M, Moosazadeh M. Efficacy of human papillomavirus l1 protein vaccines (cervarix and gardasil) in reducing the risk of cervical intraepithelial neoplasia: A meta-analysis. Int J Prev Med 2017;8:44. https://doi.org/10.4103/ijpvm.IJPVM_413_16.

[4] O Murchu E, Comber L, Jordan K, Hawkshaw S, Marshall L, O'Neill M, et al. Systematic review of the efficacy, effectiveness and safety of MF59® adjuvanted seasonal influenza vaccines for the prevention of laboratory-confirmed influenza in individuals ≥18 years of age. Reviews in Medical Virology 2023;33:e2329. https://doi.org/10.1002/rmv.2329.

[5] Sadeghalvad M, Mansourabadi AH, Noori M, Nejadghaderi SA, Masoomikarimi M, Alimohammadi M, et al. Recent developments in SARS-CoV-2 vaccines: A systematic review of the current studies. Reviews in Medical Virology 2023;33:e2359. https://doi.org/10.1002/rmv.2359.

[6] De Geus JL, Koch LF de Azevedo, Kintopp C, Spada PP, Baratto SP, Giovanini AF, et al. Are healthcare workers immunized after receiving hepatitis B vaccination according to recommended guidelines? A systematic review and meta-analysis. Int J Health Sci. 2021;15(1):35-42.

[7] Harris RC, Sumner T, Knight GM, White RG. Systematic review of mathematical models exploring the epidemiological impact of future TB vaccines. Hum Vaccin Immunother. 2016;12(11):2813-2832.

[8] Khobragade AW, Kadam DD. Efficacy of Tetravalent Dengue Vaccine: A Systematic Review and Meta-Analysis. Indian J Community Med. 2021;46(2):191-194. doi: 10.4103/ijcm.IJCM_608_20.

Wrong intervention (n=1)

[1] Young MK, Nimmo GR, Cripps AW, Jones MA. Post-exposure passive immunisation for preventing measles. Cochrane Database of Systematic Reviews 2014;2014. https://doi.org/10.1002/14651858.CD010056.pub2.
